# Comparing the Efficacy of Advanced Interleukin Inhibitors for Crohn’s Disease: A Systematic Review and Network Meta-Analysis

**DOI:** 10.3390/jcm15145593

**Published:** 2026-07-16

**Authors:** Qiuyue Zhang, Mengqi Luo, Yufang Wang

**Affiliations:** Department of Gastroenterology and Hepatology, West China Hospital of Sichuan University, Chengdu 610041, China; zhangqy730@163.com (Q.Z.); luomengqi7@stu.scu.edu.cn (M.L.)

**Keywords:** CD, RZB, GUS, MRK, UST, efficacy

## Abstract

**Background:** IL-23p19 inhibitors have recently gained approval for treating moderate-to-severe Crohn’s disease (CD). Nevertheless, their comparative efficacy relative to each other and to the IL-12/23 inhibitor ustekinumab (UST) is controversial. Therefore, this study evaluated risankizumab (RZB), mirikizumab (MRK), guselkumab (GUS), and UST for inducing and maintaining clinical and endoscopic remission in the moderate-to-severe CD population. **Methods:** PubMed, Embase, Web of Science, and Cochrane Library were searched until 4 November 2025. Risk ratios (RRs) with 95% confidence intervals (CIs) were calculated for dichotomous outcomes. The clinical and endoscopic remission/response during induction and maintenance phases was examined through a frequentist network meta-analysis (NMA). Subgroup analyses were carried out in the biologic-naïve population and those with prior biologic failure. **Results:** This NMA included 15 RCTs within 11 studies with 7747 patients. Relative to UST, IL-23p19 inhibitors demonstrated superior efficacy during induction and maintenance therapy. For clinical remission during the induction, GUS < 600 mg displayed significantly greater efficacy than UST 6 mg/kg (RR: 1.34 [95% CI: 1.06–1.70]). For endoscopic remission during the induction, both RZB > 600 mg (RR: 1.66 [95% CI: 1.14–2.41]) and RZB ≤ 600 mg (RR: 1.65 [95% CI: 1.21–2.25]) were superior to UST 6 mg/kg. During maintenance therapy, 200 mg GUS achieved a higher rate of endoscopic remission than UST (RR: 2.04 [95% CI: 1.09–3.49]). According to the SUCRA rankings, IL-23p19 inhibitors, particularly GUS and RZB, consistently ranked above UST across all evaluated outcomes. **Conclusions:** IL-23p19 inhibitors demonstrated superior efficacy in comparison to the IL-12/23 inhibitor UST in the moderate-to-severe CD population. Further high-quality, long-term, multicenter RCTs and real-world studies could confirm these findings.

## 1. Introduction

Crohn’s disease (CD), a chronic inflammatory bowel disease (IBD), features transmural inflammation and discontinuous ulcers, imposing a considerable global health burden because it is progressive and has debilitating complications such as fistulas, strictures, and malnutrition [1]. Over the past century, the incidence of IBD has risen sixfold globally. In early industrialized regions, the incidence of CD increased by 5–7% annually before stabilizing [2]. Therefore, effective therapies are urgently needed.

IL-23 binds to the IL-23 receptor expressed on T cells, particularly T-helper 17 (Th17) cells, thereby activating the downstream JAK-STAT signaling pathway and perpetuating the inflammatory response [3]. IL-23 consists of p40 and p19 subunits, among which the p19 subunit is the principal component responsible for initiating IL-23 signaling and driving inflammation in CD, whereas the p40 subunit is shared with IL-12 [4]. Ustekinumab (UST), a monoclonal antibody targeting the shared p40 subunit of IL-12 and IL-23, has demonstrated substantial clinical utility in real-world settings [5,6,7,8]. It has gained approval for treating moderate-to-severe CD as a second-line therapy following failure of TNF inhibitors or glucocorticoids [5]. IL-12 is critical in promoting Th1-cell differentiation and mediating immune responses against infectious pathogens [9]. However, inhibition of IL-12 may also lead to substantial adverse effects, including fever, flu-like symptoms, and hepatotoxicity [10]. Concerns related to the nonspecific blockade of IL-12 and IL-23 have promoted the development of next-generation IL-23p19 inhibitors, including risankizumab (RZB), mirikizumab (MRK), and guselkumab (GUS) [11,12,13,14,15,16,17,18,19]. They selectively target the p19 subunit of IL-23 without interfering with the IL-12 pathway or interferon gamma (IFN-γ) signaling, thereby effectively suppressing Th17-mediated inflammation while preserving the immune defense function of Th1 [20].

Both RZB and GUS are fully human IgG1 monoclonal antibodies targeting the p19 subunit of IL-23 [18,21]. RZB incorporates L234A and L235A mutations that reduce Fc receptor binding, thereby diminishing antibody-dependent cellular cytotoxicity and conferring a relatively long half-life of approximately 21–28 days [22]. RZB has demonstrated superior long-term efficacy in psoriasis and IBD [23,24]. In the SEQUENCE head-to-head trial, RZB outperforms UST in clinical and endoscopic outcomes in the bio-failure CD patients [25]. GUS retains intrinsic Fc receptor-binding capacity and has a half-life of around 17 days [26]. Clinically, GUS maintains high response rates over five years in psoriatic arthritis and significantly improved endoscopic outcomes in CD without increased risks of infection or malignancy [27,28,29]. MRK is a monoclonal IgG4 antibody targeting IL-23p19, with a comparatively shorter half-life of around 9.3 days, necessitating more frequent administration [30]. It has shown breakthrough results in phase III trials for CD, ulcerative colitis, and psoriasis [31,32]. The VIVID-1 trial demonstrates significantly higher endoscopic response rates at 52 weeks (68% vs. 49%) and sustained clinical remission in 93% of the CD population at two years than UST [16].

Despite these promising findings, direct comparative evidence between IL-23p19 inhibitors and UST remains limited. Given the subtle differences in molecular structure and pharmacological characteristics among IL-23p19 inhibitors, comparative evaluation both within this drug class and against UST is warranted. Therefore, the efficacy of RZB, MRK, GUS, and UST in achieving clinical and endoscopic remission in the moderate-to-severe CD population was compared via a network meta-analysis (NMA). Incorporating direct and indirect evidence, our study sought to optimize precise therapies and support evidence-based clinical decision-making.

## 2. Materials and Methods

This study followed PRISMA-NMA guides [33]. The PRISMA checklist was provided as Supplementary Material in Supplementary Table S1. The study protocol was registered in PROSPERO (CRD420251048653) on 11 May 2025.

### 2.1. Search Strategy and Study Selection

PubMed, Embase, Web of Science, and Cochrane Library were searched until 4 November 2025. Grey literature data were sourced from reference lists and Supplementary Materials of included studies. Only English-language studies were included, with no ethnicity or geographic restrictions. The search used MeSH, Emtree, and specific keywords: “CD”, “IL-23”, “RZB”, “MRK”, “GUS”, and “UST”. Two investigators independently performed the literature search and selection, and disputes were resolved through discussion until consensus was reached (Supplementary Table S2).

### 2.2. Selection Criteria

The eligibility criteria were determined according to the Population, Intervention, Comparator, Outcomes, and Study design (PICOS) framework. Inclusion criteria were: (1) randomized controlled trial (RCT); (2) moderate-to-severe CD adult patients (≥18) per established diagnostic criteria; and (3) evaluation of IL-23p19 inhibitors (RZB, MRK, or GUS) or the IL-12/23p40 inhibitor UST as the intervention. Exclusion criteria were: (1) paediatric or pregnant populations; (2) use of non-standard dosing regimens or incomplete outcome reporting; (3) animal or in vitro studies; (4) publication in languages other than English; (5) non-original articles, including case reports, commentaries, editorials, letters, reviews, clinical guidelines, or meta-analyses; or (6) non-standardized or non-extractable data.

### 2.3. Literature Selection Progress

The literature screening process was conducted in a standard and systematic manner. Two independent reviewers checked titles and abstracts from initially retrieved literature and subsequently evaluated full texts of the possibly eligible studies per the eligibility criteria. Disagreements were resolved via consensus between the two reviewers or by a third reviewer if needed.

### 2.4. Data Extraction

Two independent reviewers, employing a standard form, extracted baseline patient features, intervention details, outcome measures, and study characteristics. For efficacy, reviewers recorded the number of patients achieving clinical remission, clinical response, endoscopic remission, and endoscopic response as defined by each original trial. Outcomes were extracted at the end of induction (preferably weeks 8–16) and maintenance (preferably weeks 22–52). The specific criteria used to define each outcome, such as CDAI thresholds for clinical remission or SES-CD reduction for endoscopic response, were also documented. Dissents were addressed via discussion or by consulting a third reviewer.

### 2.5. Classification

Pre-specified dose pooling was carried out for the intervention arms of four IL-23p19 or IL-12/23 inhibitors during the induction and maintenance phases. The stratification strategy was based on the efficacy plateau at higher drug exposure levels, prioritization of approved dosing regimens, and the availability of sufficient treatment arms. The rationale for dose stratification is detailed in Supplementary Table S3.

### 2.6. Outcomes

The primary endpoint was the comparative efficacy of IL-12/23 and IL-23 inhibitors in achieving clinical and endoscopic remission during induction and maintenance phases, as defined in the original trials. Secondary endpoints included clinical and endoscopic response. When multiple assessment time points were reported, outcomes were preferentially evaluated at approximately 12 weeks (range: 8–16 weeks) for the induction phase and approximately 48 weeks (range: 22–52 weeks) for the maintenance phase. Subgroup analyses were conducted when data for the biologic-naïve population or those with prior biologic failure were available.

### 2.7. Quality Assessment

Study quality was independently rated by two authors employing the Cochrane Risk of Bias 2 (RoB 2) tool. Disagreements were settled via discussion with a third author. Evidence certainty for direct comparisons and network estimates was examined employing the CINeMA framework.

### 2.8. Statistical Analysis

Since studies available for direct head-to-head comparisons were limited, a frequentist NMA integrated direct and indirect evidence across the encompassed trials. Outcomes were reported as risk ratios (RRs) with 95% confidence intervals (CIs). The surface under the cumulative ranking curve (SUCRA) values and probability of being the best (P-best) rankings reflected the comparative efficacy of the interventions of interest and placebo (PBO). Publication bias was examined via comparison-adjusted funnel plots. Global inconsistency within the network was evaluated leveraging the design-by-treatment interaction model, whereas local inconsistency was assessed using loop-specific inconsistency tests and node-splitting analyses. Studies identified as major contributors to heterogeneity or inconsistency were iteratively excluded in sensitivity analyses. The final results were derived from the consistent network following this refinement process. All NMAs were enabled by Stata 18.0 (StataCorp LLC, College Station, TX, USA). Python 3.11.7 (Python Software Foundation, Beaverton, OR, USA) and R 4.4.3 (R Foundation for Statistical Computing, Vienna, Austria) were used for figure generation.

## 3. Results

### 3.1. Study Selection

**Figure 1:**
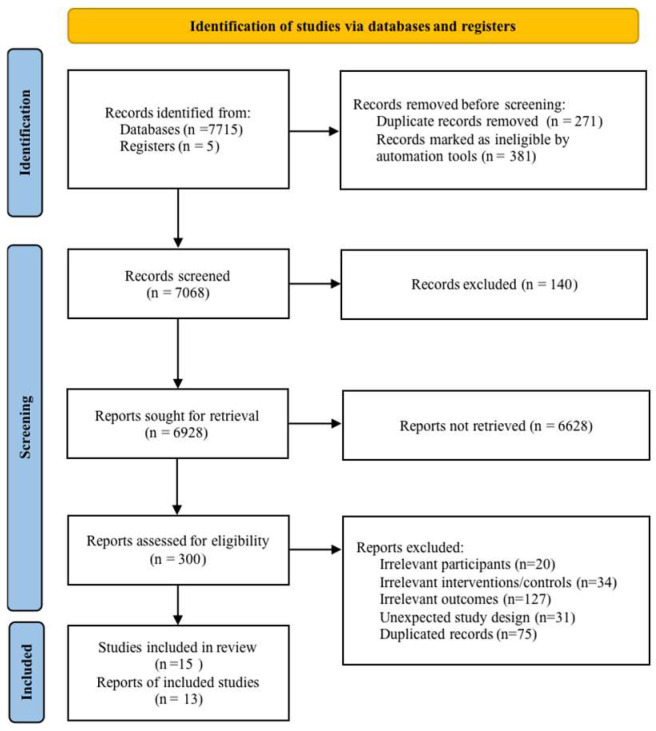
PRISMA flow diagram of study selection. For duplicates, 271 records were excluded manually, and 381 were excluded through the automation function in EndNote 21.5, which had been checked manually.

7715 studies were identified after searches until 4 November 2025. An additional five studies were identified through reference list screening and conference abstract searches. 652 duplicates were removed. 140 reports were excluded because their full-text versions could not be obtained. 6628 reports were not retrieved because their primary focus was deemed inconsistent with the PICOS framework of this network meta-analysis. The full texts of the remaining 300 articles were assessed. Ultimately, 15 RCTs reported across 13 primary publications [7,8,14,15,16,18,25,27,34,35,36,37] were encompassed (Figure 1).

### 3.2. Characteristics of Included Studies

Overall, 15 RCTs were encompassed. 11 were phase III trials. The rest were phase II trials. 7747 patients were encompassed, of whom 1692, 706, 997, and 2413 received RZB, MRK, GUS, and UST, respectively (Supplementary Table S4). Clinical remission and endoscopic remission were primarily assessed using the Crohn’s Disease Activity Index (CDAI) and the Simple Endoscopic Score for Crohn’s Disease (SES-CD) across the included studies. Detailed definitions and evaluation criteria for all outcomes are provided in Supplementary Table S5.

### 3.3. Quality Assessment

Study quality was rated via Cochrane RoB 2 (Supplementary Table S6 and Supplementary Figure S1) independently performed by two authors, with disagreements discussed with a third author. The certainty of evidence for individual pairwise comparisons and network estimates was evaluated employing the CINeMA framework (Supplementary Table S7).

### 3.4. Efficacy of Induction Therapy

**Figure 2:**
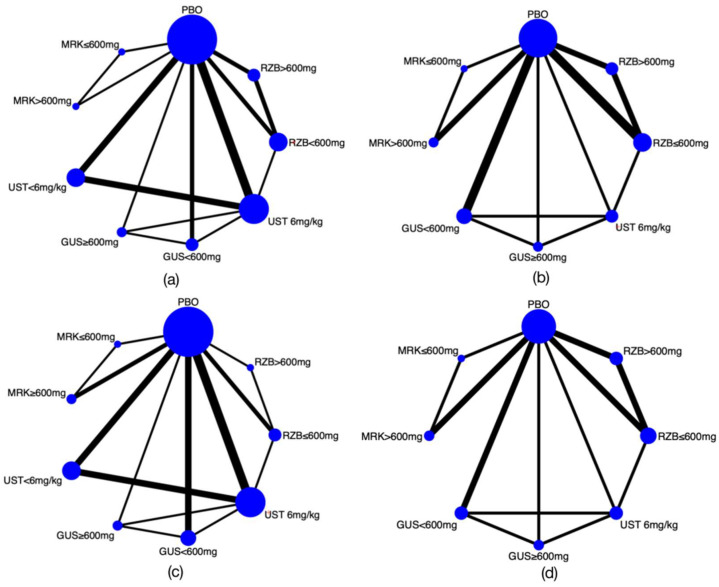
Network map of clinical and endoscopic outcomes during the induction phase. (Note: (**a**) clinical response; (**b**) endoscopic response; (**c**) clinical remission; (**d**) endoscopic remission. PBO, placebo; RZB, risankizumab; MRK, mirikizumab; GUS, guselkumab; UST, ustekinumab. The size of each circle (node) reflects the number of participants assigned to that intervention, while the thickness of the lines (connections) indicates how many studies have directly compared the linked interventions).

**Figure 3:**
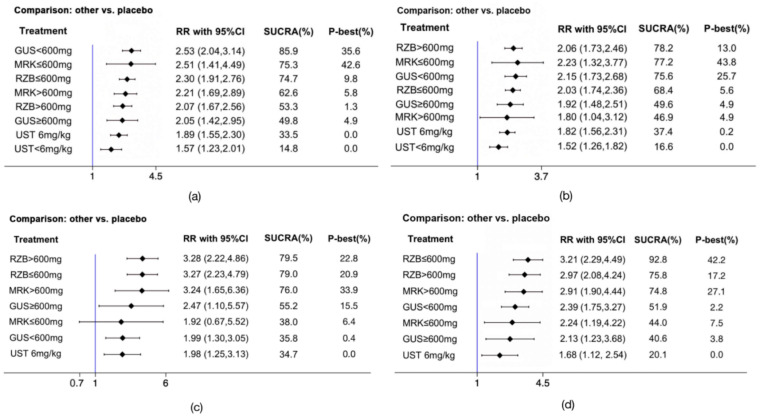
Forest plot of the intervention’s efficacy on inducing clinical outcomes with PBO as reference. (Note: (**a**) clinical remission, (**b**) clinical response, (**c**) endoscopic remission, (**d**) endoscopic response. Note: SUCRA provides a cumulative distribution of a treatment’s rankings, which quantifies the probability of it being among the most effective interventions. P-best is the probability of each intervention being ranked as best in the network. RR: risk ratio. Vertical blue line indicates the null effect (RR = 1)).

**Figure 4:**
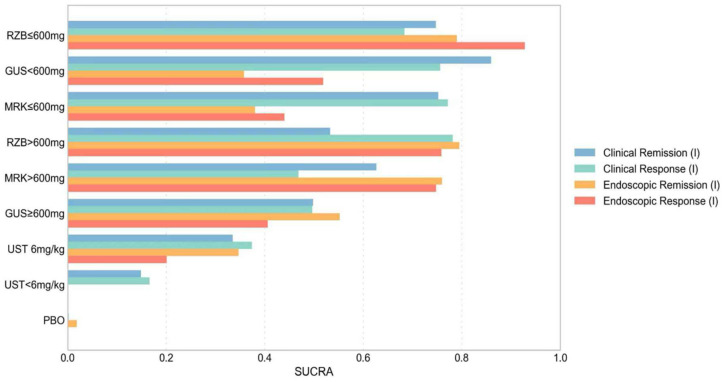
Bar plot of SUCRA values for outcomes during the induction phase across all treatments. (Note: Ordered by average of SUCRA values across outcomes. SUCRA values range from 0 to 1 and represent the percentage of efficacy that a treatment achieves relative to an ideal treatment. RZB, risankizumab; MRK, mirikizumab; GUS, guselkumab; UST, ustekinumab; PBO, placebo.).

Figure 2 shows the topological network relationships of different treatments during the induction period. Overall SUCRA rankings for the evaluated interventions are shown in Figure 3 and Figure 4. Pairwise comparisons using PBO as the reference for these outcomes are presented in Table 1 and Table 2.

#### 3.4.1. Clinical Outcomes

##### Clinical Remission

Twelve trials involving 6309 participants evaluated the efficacy of interventions for inducing clinical remission. The network heat plot had no red hotspots of inconsistency (Supplementary Figure S1). Following integration of direct and indirect evidence, RZB ≤ 600 mg demonstrated superior efficacy compared with UST < 6 mg/kg, whereas GUS < 600 mg was superior to both UST < 6 mg/kg and UST 6 mg/kg in inducing clinical remission (Table 1). According to SUCRA rankings, the top three treatment strategies were GUS < 600 mg (RR: 2.53, 95% CI: 2.04–3.14, SUCRA = 85.9), MRK ≤ 600 mg (RR: 2.51, 95% CI: 1.41–4.49, SUCRA = 75.3), and RZB ≤ 600 mg (RR: 2.30, 95% CI: 1.91–2.76, SUCRA = 74.7) (Figure 3a). Sensitivity analyses excluding RZB 200 mg q4w from the RZB ≤ 600 mg group yielded consistent findings for both clinical remission and clinical response during induction (Supplementary Figures S2 and S3). After exclusion of one trial during sensitivity analyses to eliminate global inconsistency, RZB ≤ 600 mg ranked first for inducing clinical remission (RR: 2.59, 95% CI: 2.12–3.18, SUCRA = 82.2), followed by GUS < 600 mg (RR: 2.57, 95% CI: 2.11–3.13, SUCRA = 80.3) and MRK ≤ 600 mg (RR: 2.50, 95% CI: 1.43–4.39, SUCRA = 69.6) (Supplementary Figure S4) [21].

##### Clinical Response

Eleven trials involving 5459 participants assessed the efficacy of interventions for inducing clinical response. The network heat plot had no red hotspots of inconsistency (Supplementary Figure S5). Combined direct and indirect comparisons indicated that UST < 6 mg/kg was inferior to RZB ≤ 600 mg, RZB > 600 mg, GUS < 600 mg, and UST 6 mg/kg (Table 1). Based on SUCRA rankings, the top three therapies were RZB > 600 mg (RR: 2.06, 95% CI: 1.73–2.46, SUCRA = 78.8), MRK ≤ 600 mg (RR: 2.23, 95% CI: 1.32–3.77, SUCRA = 77.2), and GUS < 600 mg (RR: 2.15, 95% CI: 1.73–2.68, SUCRA = 75.6) (Figure 3b). Following exclusion of one trial to ensure global consistency in sensitivity analyses [21], RZB ≤ 600 mg ranked first for inducing clinical response (RR: 2.29, 95% CI: 1.97–2.67, SUCRA = 81.6) (Supplementary Figure S6).

##### Biologics Experience or Failure

Among patients with prior biologic failure, 400 mg GUS q4w ranked first for both clinical remission (RR: 3.54, 95% CI: 1.92–6.55, SUCRA = 99.0) and clinical response (RR: 2.75, 95% CI: 1.77–4.27, SUCRA = 97.6) during induction (Supplementary Figure S7a,b). Among biologic-naïve participants, 200 mg GUS q4w ranked first for clinical remission (RR: 3.30, 95% CI: 1.85–5.88, SUCRA = 88.8), whereas MRK 1000 mg q4w ranked first for clinical response (RR: 16.08, 95% CI: 0.99–260.85, SUCRA = 85.9) (Supplementary Figure S8a). However, the 95% CIs for MRK at doses of 200 mg, 600 mg, and 1000 mg q4w crossed the null value. Except for MRK, 400 mg GUS q4w demonstrated the greatest efficacy for inducing clinical response among biologic-naïve patients (RR: 1.80, 95% CI: 1.25–2.59, SUCRA = 41.3).

#### 3.4.2. Endoscopic Outcomes

##### Endoscopic Remission

Seven trials involving 3505 participants evaluated the efficacy of interventions for inducing endoscopic remission. The inconsistency was not substantial (Supplementary Figure S9). Except for MRK ≤ 600 mg, all interventions significantly outperformed PBO in achieving endoscopic remission (Table 2). UST 6 mg/kg demonstrated significantly lower efficacy than both RZB ≤ 600 mg and RZB > 600 mg (Table 2). Relative to PBO, RZB > 600 mg (RR: 3.28, 95% CI: 2.22–4.86), RZB ≤ 600 mg (RR: 3.27, 95% CI: 2.23–4.79), and MRK > 600 mg (RR: 3.24, 95% CI: 1.65–6.36) achieved the three highest SUCRA values (79.5, 79.0, and 76.0, respectively) (Figure 3c).

##### Endoscopic Response

Eight trials involving 4294 patients assessed the efficacy of interventions for inducing endoscopic response. The inconsistency was not significant (Supplementary Figure S10). UST 6 mg/kg was significantly inferior to both RZB ≤ 600 mg and RZB > 600 mg in inducing an endoscopic response (Table 2). RZB ≤ 600 mg demonstrated the highest efficacy estimate (RR: 3.21, 95% CI: 2.29–4.49) and the highest SUCRA value (92.8). RZB > 600 mg (RR: 2.97, 95% CI: 2.08–4.24, SUCRA = 75.8) and MRK > 600 mg (RR: 2.91, 95% CI: 1.90–4.44, SUCRA = 74.8) ranked second and third, respectively (Figure 3d).

##### Biologics Experience or Failure

Among patients with prior biologic failure, 600 mg RZB q4w ranked first for inducing endoscopic remission (RR: 3.58, 95% CI: 1.45–8.84, SUCRA = 82.7) (Supplementary Figure S8c). 200 mg GUS q4w (RR: 3.72, 95% CI: 1.84–7.50, SUCRA = 98.9) and 600 mg MRK q4w (RR: 4.85, 95% CI: 1.14–20.52, SUCRA = 77.4) ranked first among biologic-failure and biologic-naïve patients, respectively (Supplementary Figures S7d and S8c).

### 3.5. Efficacy of Maintenance Therapy

**Figure 5:**
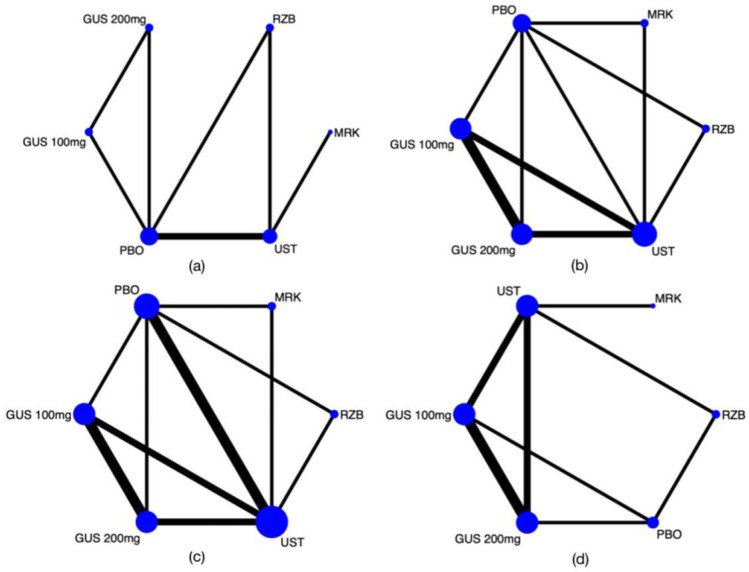
Network map of clinical and endoscopic outcomes during the maintenance phase. (Note: (**a**) clinical response; (**b**) endoscopic response; (**c**) clinical remission; (**d**) endoscopic remission. PBO, placebo; RZB, risankizumab; MRK, mirikizumab; GUS, guselkumab; UST, ustekinumab. The size of each circle (node) reflects the number of participants assigned to that intervention, while the thickness of the lines (connections) indicates how many studies have directly compared the linked interventions).

**Figure 6:**
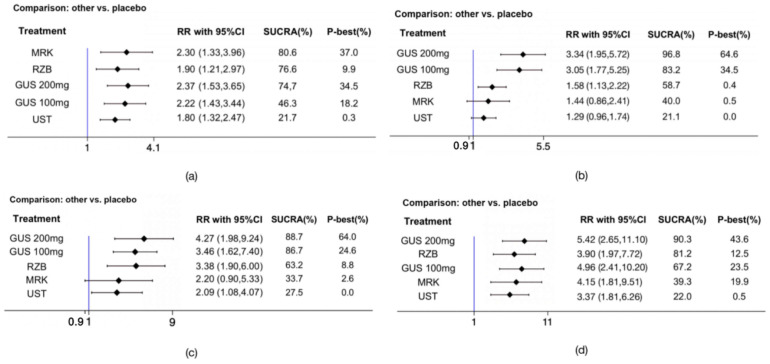
Forest plot of the intervention’s efficacy on maintaining clinical outcomes with PBO as reference. (Note: (**a**) clinical remission, (**b**) clinical response, (**c**) endoscopic remission, (**d**) endoscopic response. Note: SUCRA provides a cumulative distribution of a treatment’s rankings, which quantifies the probability of it being among the most effective interventions. P-best is the probability of each intervention being ranked as best in the network. RR: risk ratio. Vertical blue line indicates the null effect (RR = 1)).

**Figure 7:**
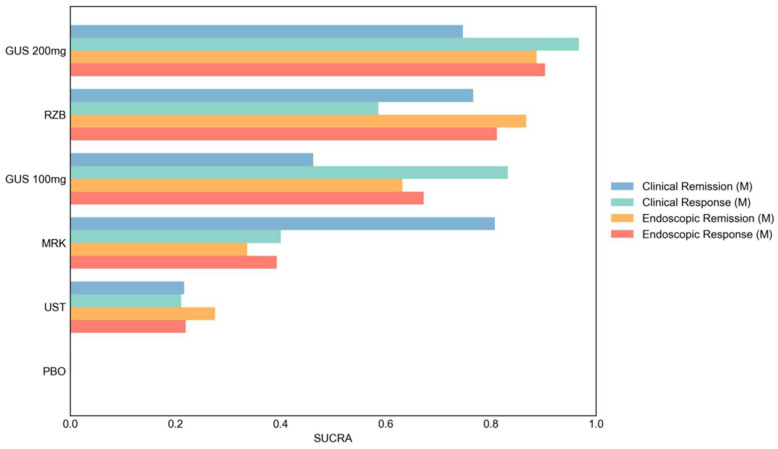
Bar plot of SUCRA values for outcomes during the maintenance phase across all treatments. (Note: Ordered by average of SUCRA values across outcomes. SUCRA values range from 0 to 1 and represent the percentage of efficacy that a treatment achieves relative to an ideal treatment. RZB, risankizumab; MRK, mirikizumab; GUS, guselkumab; UST, ustekinumab; PBO, placebo.).

Figure 5 illustrates the topological network relationships of different treatments during the maintenance period. Overall SUCRA rankings are presented in Figure 6 and Figure 7. Pairwise comparisons using PBO as the reference are summarized in Table 3 and Table 4.

#### 3.5.1. Clinical Outcomes

##### Clinical Remission

Eight RCTs involving 4048 patients evaluated the efficacy of interventions for maintaining clinical remission. Red and blue hotspots in the network heat plot indicated strong direct and indirect evidence within the network (Supplementary Figure S11). MRK achieved the highest SUCRA value (RR: 2.30, 95% CI: 1.33–3.96, SUCRA = 80.6), whereas 200 mg GUS yielded the highest point estimate (RR: 2.37, 95% CI: 1.53–3.65). UST demonstrated comparatively modest efficacy (RR: 1.80, 95% CI: 1.32–2.47, SUCRA = 21.7) (Figure 6a). After exclusion of two trials during sensitivity analyses to eliminate overall inconsistency, 200 mg GUS ranked first (RR: 2.75, 95% CI: 2.19–3.47, SUCRA = 90.1), followed by MRK (RR: 2.71, 95% CI: 2.16–3.40, SUCRA = 82.0). RZB demonstrated comparatively modest efficacy (RR: 1.32, 95% CI: 1.07–1.63, SUCRA = 19.9) (Supplementary Figure S12).

##### Clinical Response

Six trials involving 2738 participants assessed the efficacy of interventions for maintaining clinical response. The network heat plot revealed no substantial inconsistency (Supplementary Figure S13). UST and MRK showed a positive trend toward maintaining clinical response; however, the 95% CIs were wide and crossed the null value, indicating limited certainty of evidence (Table 3). 200 mg GUS (RR: 3.34, 95% CI: 1.95–5.72, SUCRA = 96.8) and 100 mg GUS (RR: 3.05, 95% CI: 1.77–5.25, SUCRA = 83.2) ranked first and second, respectively (Figure 6b). These findings remained stable after exclusion of one trial to eliminate global inconsistency in sensitivity analyses (Supplementary Figure S14).

##### Biologics Experience or Failure

200 mg GUS q4w ranked first for maintaining clinical remission among both the biologic-failure (RR: 4.80, 95% CI: 2.95–7.81, SUCRA = 87.5) and biologic-naïve populations (RR: 1.74, 95% CI: 1.29–2.34, SUCRA = 78.2) (Supplementary Figures S8d and S15a).

#### 3.5.2. Endoscopic Outcomes

##### Endoscopic Remission

Six trials involving 3316 participants evaluated the efficacy of interventions for maintaining endoscopic remission. The inconsistency was insignificant (Supplementary Figure S16). Based on integrated direct and indirect evidence, 200 mg GUS demonstrated significantly greater efficacy than UST (Table 4). 200 mg GUS ranked first (RR: 4.27, 95% CI: 1.98–9.24, SUCRA = 88.7), followed closely by 100 mg GUS (RR: 3.46, 95% CI: 1.62–7.40, SUCRA = 86.7). RZB ranked third (RR: 3.38, 95% CI: 1.90–6.00, SUCRA = 63.2) (Figure 6c). After exclusion of one trial to ensure global consistency, RZB emerged as the top-ranked therapy (RR: 7.57, 95% CI: 3.27–17.52, SUCRA = 95.7), followed by GUS 100 mg and 200 mg GUS (Supplementary Figure S17).

##### Endoscopic Response

Six trials involving 3516 participants assessed the efficacy of interventions for maintaining endoscopic response. The comparison-adjusted heat plot demonstrated the strongest direct evidence between PBO versus RZB and RZB versus UST, as indicated by prominent red shading (Supplementary Figure S18). 200 mg GUS ranked first (RR: 5.42, 95% CI: 2.65–11.10, SUCRA = 90.3), whereas RZB ranked second (RR: 3.90, 95% CI: 1.97–7.72, SUCRA = 81.2) (Figure 6d). Following exclusion of one trial during sensitivity analyses to eliminate overall inconsistency, RZB overwhelmingly ranked first (RR: 10.55, 95% CI: 6.62–16.80, SUCRA = 99.7) (Supplementary Figure S19).

##### Biologics Experience or Failure

Among patients with prior biologic failure, 360 mg RZB q8w ranked first for maintaining endoscopic remission (RR: 4.76, 95% CI: 1.46–15.56, SUCRA = 93.6) (Supplementary Figure S15b). Five trials reported maintenance endoscopic response among biologic-naïve participants, among whom 200 mg GUS q4w ranked first (RR: 11.71, 95% CI: 5.23–26.25, SUCRA = 98.3) (Supplementary Figure S15d).

### 3.6. Safety Outcomes

Safety data from the included trials indicated that IL-23p19 inhibitors had generally similar rates of adverse events, serious adverse events, and serious infections compared with ustekinumab and placebo, with no new safety signals identified. Safety event rates for each treatment are detailed in Supplementary Table S8 and the accompanying narrative summary.

### 3.7. Sensitivity Test

To assess robustness against the heterogeneity, two prespecified pairwise meta-analyses stratified by induction week and exact dosing regimens were performed. Effect estimates remained consistent across time points and doses, and the ranking superiority of IL-23p19 inhibitors over UST was stable, confirming that neither timing nor dose pooling materially biased our conclusions (Supplementary Figures S20–S34).

## 4. Discussion

Our systematic review and NMA provide a comprehensive comparison of the efficacy of RZB, MRK, GUS, and UST on clinical and endoscopic outcomes during induction and maintenance therapy in the CD population. By synthesizing evidence from 15 randomized controlled trials reported across 13 primary publications involving 7747 participants, this analysis provides important insights into the relative performance of these agents across multiple endpoints, thereby addressing the limited availability of direct head-to-head trials. Throughout this discussion, we emphasise the point estimates and 95% confidence intervals of the risk ratios for key comparisons, and report ranking metrics such as SUCRA only as exploratory descriptive statistics rather than as definitive evidence of superiority.

During the induction phase, lower-dose GUS regimens (200 mg IV q4w and 400 mg SC q4w) (RR: 2.53, 95% CI: 2.04–3.14) represented the most efficacious treatment strategies for achieving clinical remission in the overall population. Lower-dose MRK (200 mg and 600 mg IV q4w) (RR: 2.51, 95% CI: 1.41–4.49) and RZB (200 mg and 600 mg IV q4w) (RR: 2.30, 95% CI: 1.91–2.76) were also associated with substantial early clinical benefits in the moderate-to-severe CD population. The American Gastroenterological Association (AGA) guideline similarly reported that, among biologic-experienced patients, RZB and GUS were significantly superior to UST in inducing clinical remission, with moderate-to-high certainty evidence [38]. Furthermore, GUS, RZB, and MRK at lower doses demonstrated broadly comparable efficacy in inducing clinical response in our analysis. Another NMA has reported that, irrespective of prior biologic exposure, 600 mg RZB ranks first for induction of clinical remission, whereas 1200 mg RZB ranks first for clinical response during induction [39]. Regarding endoscopic outcomes, RZB, particularly at lower induction doses (RR: 3.21, 95% CI: 2.29–4.49), demonstrated superior performance in achieving endoscopic response in our analysis, suggesting a potentially stronger short-term effect on mucosal healing. RZB 1200 mg (RR: 3.28, 95% CI: 2.22–4.86) demonstrated the greatest efficacy for inducing endoscopic remission.

During the maintenance phase, 200 mg GUS emerged as the most efficacious regimen for achieving endoscopic response (RR: 5.42, 95% CI: 2.65–11.10) and endoscopic remission (RR: 4.27, 95% CI: 1.98–9.24). A recent NMA conducted by Disher et al. Has similarly identified 200 mg GUS as a leading therapy because of its consistently strong performance across one-year efficacy outcomes in the moderately to severely active CD population [40]. Both analyses demonstrated superior efficacy of GUS over UST for endoscopic outcomes, as well as a high ranking for clinical response. Regarding clinical remission during the maintenance period, Disher et al. identified 200 mg GUS q4w as the most favourable advanced therapy, whereas MRK achieved the highest ranking in our primary analysis. After exclusion of two trials during sensitivity analyses [25], which were also excluded in a prior NMA [40], our findings became consistent with those previously reported.

Subgroup analyses further demonstrated that 400 mg GUS q4w was particularly effective among patients with prior biologic failure during induction therapy, ranking highest for both clinical remission and clinical response. Among the biologic-naïve population, 200 mg GUS q4w appeared most effective for achieving both clinical and endoscopic remission. 600 mg MRK q4w and 1000 mg MRK q4w may also be considered for rapid induction of clinical remission and clinical response, respectively; however, the wide CIs associated with MRK indicate uncertainty regarding these estimates. Overall, for biologic-naïve patients, 200 mg GUS q4w appears to represent an optimal therapeutic option because of its balanced efficacy across both clinical and endoscopic outcomes during induction and maintenance therapy. For patients with prior biologic failure, 400 mg GUS q4w may be preferred for induction therapy before 200 mg GUS q4w during maintenance, particularly when clinical outcomes are prioritized. In contrast, 600 mg RZB during induction and 360 mg RZB during maintenance may be preferable when endoscopic remission is the primary therapeutic target in bio-failure patients. The robust efficacy of GUS in biologic-failure populations is consistent with the findings reported by Disher et al. [40].

This NMA consistently showed that UST was less effective than the new IL-23p19 antagonists in both induction and maintenance therapy in moderate-to-severe CD. These findings align with another meta-analysis that incorporated five head-to-head randomized trials [41]. The superior efficacy of IL-23p19 antagonists appears to be driven predominantly by patients with prior biologic exposure, especially previous TNF antagonist therapy, in whom UST demonstrates comparatively lower efficacy [41]. Moreover, two independent NMAs reported that RZB may have a higher probability of inducing clinical remission than UST in moderate-to-severe CD [39,42]. Although another NMA confirmed the sustained superiority of IL-23 inhibitors for achieving clinical remission, endoscopic remission, and endoscopic response over one year of maintenance therapy, 90 mg UST q12w remained the second-best therapy after GUS for inducing clinical response [40]. The divergent efficacy between selective IL-23p19 inhibitors and dual IL-12/23p40 blockade reflects their distinct immunological roles in intestinal inflammation. IL-12 drives early-phase inflammation via IFN-gamma-mediated Th1 responses, whereas IL-23 perpetuates chronic inflammation through IL-17-producing Th17 cells [43]. In established Crohn’s disease, IL-23 is the dominant pathogenic driver, with anti-TNF non-responders showing marked mucosal IL-23p19, IL-23R, and IL-17A upregulation alongside expansion of TNFR2+IL23R+ T cells [44]. Mechanistically, IL-12 and IL-23 exhibit independent, non-redundant functions: IL-12p35 deficiency impairs Th1 but preserves Th17 responses, while IL-23p19 deficiency impairs Th17 but preserves Th1 immunity [45]. Dual p40 blockade simultaneously abrogates both Th1 and Th17 pathways, eliminating pathogenic IL-23 signaling while also compromising IL-12-dependent protective immunity against intracellular pathogens and anti-tumor surveillance [46,47]. Selective IL-23p19 inhibition therefore offers a more targeted approach by preserving IL-12-mediated host defense while suppressing chronic intestinal inflammation. Taken together, the relatively lower performance of UST may be attributed to its dual actions on both the p40 subunit of IL-12 and IL-23, representing a form of “over-targeting” that modulates both pathogenic Th17 and protective Th1 pathways [3].

While our network meta-analysis shows IL-23p19 inhibitors superior to UST, biologic therapy is not the sole approach. Dietary interventions such as plant-based and whole-food diets have shown promise in mild-to-moderate CD (whole-food diet OR 1.41, 95% CI 1.03–1.93 for clinical remission) [48], and combining biologics with the Crohn’s Disease Exclusion Diet and partial enteral nutrition may offer early benefits [49], yet head-to-head comparative studies against dietary strategies are lacking. Beyond the IL-12/IL-23 axis, the JAK1 inhibitor upadacitinib ranks highly for endoscopic remission [50], anti-TNF agents like infliximab remain mainstays especially with immunomodulators [51]. CARD9-targeting small molecules are emerging, autologous haematopoietic stem cell transplantation serves as salvage for refractory disease [52], and IL23R-CAR T-regulatory cells show enhanced preclinical activity [53]. These developments, from diet to cellular therapies, highlight a therapeutic landscape far broader than biologics alone, and our findings should be interpreted accordingly.

This study offers several major strengths. Its large evidence base comprises 15 RCTs (7747 patients), synthesized via rigorous frequentist network meta-analysis, enabling robust indirect comparisons. Additionally, prespecified subgroup analyses by prior biologic exposure yield actionable insights for biologic-naïve versus failure populations, and sensitivity analyses stratified by induction duration and dosing consistently confirmed stable rankings. However, there are some limitations. First, the evidence includes phase II/III trials, and though inconsistency was not significant, heterogeneity may bias results. Second, support for MRK came from a limited sample, affecting precision. In addition, not all trials reported endoscopic assessments in all participants. Future work should incorporate ulcer healing, inflammatory markers, quality of life, and long-term safety.

## 5. Conclusions

This systematic review and NMA demonstrated the superior efficacy of IL-23p19 antagonists, particularly GUS and RZB, compared with UST for induction and maintenance therapy in moderate-to-severe CD. Future head-to-head trials and real-world studies could corroborate these findings, determine optimal dosing strategies, and further evaluate the long-term efficacy and safety of these promising biologic therapies, particularly across specific patient phenotypes.

## Figures and Tables

**Table 1:** League table of comparative efficacy of different IL-23 and IL-12/23 agents on clinical remission and clinical response during induction phases.

	PBO	RZB ≤ 600 mg	RZB > 600 mg	MRK ≤ 600 mg	MRK > 600 mg	GUS < 600 mg	GUS ≥ 600 mg	UST < 6 mg/kg	UST 6 mg/kg
PBO	PBO	2.03 (1.74, 2.36)	2.06 (1.73, 2.46)	2.23 (1.32, 3.77)	1.80 (1.04, 3.12)	2.15 (1.73, 2.68)	1.92 (1.48, 2.51)	1.52 (1.26, 1.82)	1.82 (1.56, 2.13)
RZB ≤ 600 mg	2.30 (1.91, 2.76)	RZB ≤ 600 mg	1.02 (0.88, 1.17)	1.10 (0.64, 1.90)	0.89 (0.50, 1.57)	1.06 (0.82, 1.37)	0.95 (0.71, 1.27)	0.75 (0.60, 0.93)	0.90 (0.75, 1.07)
RZB > 600 mg	2.07 (1.67, 2.56)	0.90 (0.76, 1.06)	RZB > 600 mg	1.08 (0.62, 1.88)	0.87 (0.49, 1.55)	1.04 (0.79, 1.38)	0.93 (0.68, 1.28)	0.74 (0.58, 0.94)	0.88 (0.71, 1.10)
MRK ≤ 600 mg	2.51 (1.41, 4.49)	1.10 (0.59, 2.02)	1.22 (0.65, 2.27)	MRK ≤ 600 mg	0.81 (0.54, 1.20)	0.96 (0.55, 1.70)	0.86 (0.48, 1.55)	0.68 (0.39, 1.18)	0.81 (0.47, 1.41)
MRK > 600 mg	2.21 (1.69, 2.89)	0.96 (0.69, 1.34)	1.07 (0.75, 1.51)	0.88 (0.51, 1.52)	MRK > 600 mg	1.19 (0.66, 2.16)	1.07 (0.58, 1.97)	0.84 (0.47, 1.50)	1.01 (0.57, 1.79)
GUS < 600 mg	2.53 (2.04, 3.14)	1.10 (0.84, 1.44)	1.22 (0.91, 1.65)	1.01 (0.54, 1.87)	1.15 (0.82, 1.61)	GUS < 600 mg	0.89 (0.70, 1.14)	0.71 (0.55, 0.91)	0.85 (0.68, 1.05)
GUS ≥ 600 mg	2.05 (1.42, 2.95)	0.89 (0.60, 1.32)	0.99 (0.65, 1.50)	0.82 (0.41, 1.61)	0.93 (0.59, 1.45)	0.81 (0.58, 1.13)	GUS ≥ 600 mg	0.79 (0.60, 1.04)	0.95 (0.74, 1.21)
UST < 6 mg/kg	1.57 (1.23, 2.01)	0.68 (0.52, 0.91)	0.76 (0.55, 1.05)	0.63 (0.33, 1.17)	0.71 (0.50, 1.02)	0.62 (0.46, 0.83)	0.77 (0.51, 1.15)	UST < 6 mg/kg	1.20 (1.03, 1.40)
UST 6 mg/kg	1.89 (1.55, 2.30)	0.82 (0.66, 1.02)	0.91 (0.70, 1.19)	0.75 (0.41, 1.38)	0.86 (0.62, 1.19)	0.75 (0.59, 0.95)	0.92 (0.64, 1.32)	1.20 (0.98, 1.47)	UST 6 mg/kg

Note: Comparisons should be read from left to right. The risk ratio (RR) shown in each cell corresponds to the comparison between the treatment defined in the row and the treatment defined in the column. An RR greater than 1 indicates a favourable outcome for the row-defining treatment when assessing clinical remission and the column-defining treatment when assessing clinical response. Statistically significant values are highlighted in yellow. Blue shading indicates the dose group label. Results were expressed as RRs with 95% CIs. RZB, risankizumab; MRK, mirikizumab; GUS, guselkumab; UST, ustekinumab; PBO, placebo.

**Table 2:** League table of comparative efficacy of different IL-23 and IL-12/23 agents on endoscopic remission and endoscopic response during the induction phase.

	PBO	RZB ≤ 600 mg	RZB > 600 mg	MRK ≤ 600 mg	MRK > 600 mg	GUS < 600 mg	GUS ≥ 600 mg	UST 6 mg/kg
PBO	PBO	3.21 (2.29, 4.49)	2.97 (2.08, 4.24)	2.24 (1.19, 4.22)	2.91 (1.90, 4.44)	2.39 (1.75, 3.27)	2.13 (1.23, 3.68)	1.68 (1.12, 2.54)
RZB ≤ 600 mg	3.27 (2.23, 4.79)	RZB ≤ 600 mg	0.93 (0.70, 1.22)	0.70 (0.34, 1.43)	0.91 (0.53, 1.56)	0.75 (0.49, 1.13)	0.66 (0.37, 1.19)	0.53 (0.37, 0.75)
RZB > 600 mg	3.28 (2.22, 4.86)	1.00 (0.80, 1.25)	RZB > 600 mg	0.75 (0.36, 1.56)	0.98 (0.56, 1.70)	0.80 (0.52, 1.25)	0.72 (0.39, 1.32)	0.57 (0.37, 0.87)
MRK ≤ 600 mg	1.92 (0.67, 5.52)	0.59 (0.19, 1.81)	0.58 (0.19, 1.80)	MRK ≤ 600 mg	1.30 (0.76, 2.21)	1.07 (0.53, 2.15)	0.95 (0.41, 2.19)	0.75 (0.36, 1.59)
MRK > 600 mg	3.24 (1.65, 6.36)	0.99 (0.46, 2.15)	0.99 (0.45, 2.15)	1.68 (0.73, 3.92)	MRK > 600 mg	0.82 (0.49, 1.39)	0.73 (0.37, 1.46)	0.58 (0.32, 1.04)
GUS < 600 mg	1.99 (1.30, 3.05)	0.61 (0.36, 1.03)	0.61 (0.36, 1.04)	1.04 (0.33, 3.24)	0.62 (0.28, 1.37)	GUS < 600 mg	0.89 (0.54, 1.48)	0.70 (0.46, 1.07)
GUS ≥ 600 mg	2.47 (1.10, 5.57)	0.76 (0.33, 1.73)	0.75 (0.32, 1.75)	1.29 (0.34, 4.88)	0.76 (0.27, 2.20)	1.24 (0.57, 2.69)	GUS ≥ 600 mg	0.79 (0.45, 1.39)
UST 6 mg/kg	1.98 (1.25, 3.13)	0.60 (0.44, 0.82)	0.60 (0.41, 0.88)	1.03 (0.33, 3.25)	0.61 (0.27, 1.38)	0.99 (0.57, 1.72)	0.80 (0.35, 1.82)	UST 6 mg/kg

Note: Comparisons should be read from left to right. The RR shown in each cell corresponds to the comparison between the treatment defined in the row and the treatment defined in the column. An RR greater than 1 indicates a favourable outcome for the row-defining treatment when assessing endoscopic remission and the column-defining treatment when assessing endoscopic response. Statistically significant values are highlighted in yellow. Blue shading indicates the dose group label. Results were expressed as RRs with 95% CIs. RZB, risankizumab; MRK, mirikizumab; GUS, guselkumab; UST, ustekinumab; PBO, placebo.

**Table 3:** League table of comparative efficacy of different IL-23 and IL-12/23 agents on clinical remission and clinical response during the maintenance phase.

	PBO	RZB	MRK	GUS 100 mg	GUS 200 mg	UST
PBO	PBO	1.58 (1.13, 2.22)	1.44 (0.86, 2.41)	3.05 (1.77, 5.25)	3.34 (1.95, 5.72)	1.29 (0.96, 1.74)
RZB	1.90 (1.21, 2.97)	RZB	0.91 (0.53, 1.56)	1.93 (1.02, 3.65)	2.11 (1.12, 3.98)	0.81 (0.58, 1.14)
MRK	2.30 (1.33, 3.96)	1.21 (0.62, 2.35)	MRK	2.12 (1.00, 4.47)	2.32 (1.10, 4.88)	0.90 (0.59, 1.36)
GUS 100 mg	2.22 (1.43, 3.44)	1.17 (0.67, 2.05)	0.97 (0.51, 1.82)	GUS 100 mg	1.09 (0.71, 1.69)	0.42 (0.23, 0.78)
GUS 200 mg	2.37 (1.53, 3.65)	1.25 (0.71, 2.18)	1.03 (0.55, 1.93)	1.07 (0.76, 1.50)	GUS 200 mg	0.39 (0.21, 0.72)
UST	1.80 (1.32, 2.47)	0.95 (0.61, 1.48)	0.78 (0.46, 1.33)	0.81 (0.56, 1.18)	0.76 (0.53, 1.10)	UST

Note: Comparisons should be read from left to right. The RR shown in each cell corresponds to the comparison between the treatment defined in the row and the treatment defined in the column. An RR greater than 1 indicates a favorable outcome for the row-defining treatment when assessing clinical remission and the column-defining treatment when assessing clinical response. Statistically significant values are highlighted in yellow. Blue shading indicates the dose group label. Results were expressed as RRs with 95% CIs. RZB, risankizumab; MRK, mirikizumab; GUS, guselkumab; UST, ustekinumab; PBO, placebo.

**Table 4:** League table of comparative efficacy of different IL-23 and IL-12/23 agents on endoscopic remission and endoscopic response during the maintenance phase.

	PBO	RZB	MRK	GUS 100 mg	GUS 200 mg	UST
PBO	PBO	3.90 (1.97, 7.72)	4.15 (1.81, 9.51)	4.96 (2.41, 10.20)	5.42 (2.65, 11.10)	3.37 (1.81, 6.26)
RZB	3.38 (1.90, 6.00)	RZB	1.07 (0.41, 2.77)	1.27 (0.56, 2.91)	1.39 (0.61, 3.17)	0.86 (0.44, 1.71)
MRK	2.20 (0.90, 5.33)	0.65 (0.29, 1.44)	MRK	1.20 (0.48, 2.98)	1.31 (0.53, 3.25)	0.81 (0.37, 1.76)
GUS 100 mg	3.46 (1.62, 7.40)	1.03 (0.50, 2.12)	1.58 (0.72, 3.47)	GUS 100 mg	1.09 (0.67, 1.79)	0.68 (0.39, 1.18)
GUS 200 mg	4.27 (1.98, 9.24)	1.26 (0.61, 2.64)	1.95 (0.88, 4.31)	1.23 (0.85, 1.78)	GUS 200 mg	0.62 (0.36, 1.07)
UST	2.09 (1.08, 4.07)	0.62 (0.36, 1.07)	0.95 (0.53, 1.72)	0.60 (0.36, 1.02)	0.49 (0.29, 0.84)	UST

Note: Comparisons should be read from left to right. The RR shown in each cell corresponds to the comparison between the treatment defined in the row and the treatment defined in the column. An RR greater than 1 indicates a favourable outcome for the row-defining treatment when assessing endoscopic remission and the column-defining treatment when assessing endoscopic response. Statistically significant values are highlighted in yellow. Blue shading indicates the dose group label. Results were expressed as RRs with 95% CIs. RZB, risankizumab; MRK, mirikizumab; GUS, guselkumab; UST, ustekinumab; PBO, placebo.

## Data Availability

The analyzed data are available in the article and online Supplementary Materials.

## Supplementary Materials

The following supporting information can be downloaded at https://www.mdpi.com/article/10.3390/jcm15145593/s1: Table S1. PRISMA 2020 Checklist.

## Abbreviations

CDAI, Crohn’s disease activity index; CD, Crohn’s disease; CDEIS, Crohn’s Disease Endoscopic Index of Severity; CI, confidence interval; CINeMA, confidence in network meta-analysis; GUS, guselkumab; IBD, inflammatory bowel disease; IFN-γ, interferon gamma; IgG, immunoglobulin G; IL, interleukin; IV, intravenous; JAK-STAT, Janus kinase-signal transducer and activator of transcription; MRK, mirikizumab; PBO, placebo; P-best, probability of being the best; PRISMA-NMA, Preferred Reporting Items for Systematic Reviews and Meta-Analyses extension for Network Meta-Analyses; RCT, randomized controlled trial; RR: risk ratio; RZB, risankizumab; SC, subcutaneous; SES-CD, Simple Endoscopic Score for Crohn’s Disease; SUCRA, surface under the cumulative ranking curve; Th, T helper cell; UST, ustekinumab.
