# Supplementary material for: Comparing the Efficacy of Advanced Interleukin Inhibitors for Crohn’s Disease: A Systematic Review and Network Meta-Analysis
Source: J Clin Med. 2026 Jul 16;15(14):5593. doi: 10.3390/jcm15145593 (PMC13413369; doi:10.3390/jcm15145593)
Supplement: Supplementary file 1 [file jcm-15-05593-s001.zip › jcm-4402791-supplementary.pdf]

SUPPLEMENTAL MATERIALS

Supplementary Table S1. PRISMA 2020 Checklist.
Supplementary Table S2. Detailed search strategies.
Supplementary Table S3. Stratification of treatment arms for nodes in Network meta-analysis.
Supplementary Table S4. Characteristics of included trails.
Supplementary Table S5. Definition of clinical and endoscopic outcomes from included trails.
Supplementary Table S6. Cochrane Risk of Bias for Randomized Trials.
Supplementary Table S7. Confidence in network meta-analysis evaluating the confidence of indirect and direct evidence by CINeMA.
Supplementary Table S8. Summary of safety outcomes from randomized controlled trials included in the network meta-analysis.
Supplementary Figure S1. Network heat plot for clinical remission during induction.
Supplementary Figure S2. Forest plot of sensitivity analysis for inducing the clinical remission among all participants, excluding Risankizumab 200mg iv q4w from RZB≤600mg.
Supplementary Figure S3. Forest plot of sensitivity analysis for inducing the clinical response among all participants, excluding Risankizumab 200mg iv q4w from RZB≤600mg.
Supplementary Figure S4. Forest plot of Sensitivity analysis for inducing the clinical remission among all participants, excluding ADVANCE.
Supplementary Figure S5. Network heat plot for clinical response during induction.
Supplementary Figure S6. Forest plot of Sensitivity analysis for inducing the clinical response among all participants, excluding ADVANCE.
Supplementary Figure S7. Forest plot of interventions’ efficacy on inducing clinical and endoscopic outcomes with placebo as reference among previous bio-failure participants.
Supplementary Figure S8. Forest plot of interventions’ efficacy on inducing and maintaining clinical and endoscopic outcomes with placebo as reference among previous bio-naive participants.
Supplementary Figure S9. Network heat plot for endoscopic remission during induction.
Supplementary Figure S10. Network heat plot for endoscopic response during induction.
Supplementary Figure S11. Network heat plot for clinical remission during maintenance.
Supplementary Figure S12. Forest plot of sensitivity analysis for maintaining the clinical remission among all participants, excluding SEQUENCE and CERTIFI.
Supplementary Figure S13. Network heat plot for clinical response during maintenance.
Supplementary Figure S14. Forest plot of sensitivity analysis for maintaining the clinical response among all participants, excluding CERTIFI.
Supplementary Figure S15. Forest plot of interventions’ efficacy on maintaining clinical and endoscopic outcomes with placebo as reference among previous bio-failure participants.
Supplementary Figure S16. Network heat plot for endoscopic remission during maintenance.
Supplementary Figure S17. Forest plot of sensitivity analysis for maintaining the endoscopic remission among all participants, excluding FORTIFY.
Supplementary Figure S18. Network heat plot for endoscopic response during maintenance.
Supplementary Figure S19. Forest plot of sensitivity analysis for maintaining the endoscopic response among all participants, excluding FORTIFY.
Supplementary Figure S20.Forest plot of dose-stratified traditional Meta-Analysis comparing efficacy of individual IL-23p19 inhibitor doses against placebo on clinical remission during induction.

Supplementary Figure S21.Forest plot of dose-stratified traditional Meta-Analysis comparing efficacy of individual IL-23p19 inhibitor doses against placebo on clinical response during induction.
Supplementary Figure S22.Forest plot of dose-stratified traditional Meta-Analysis comparing efficacy of individual IL-23p19 inhibitor doses against placebo on endoscopic remission during induction.
Supplementary Figure S23. Forest plot of dose-stratified traditional Meta-Analysis comparing efficacy of individual IL-23p19 inhibitor doses against placebo on endoscopic response during induction.
Supplementary Figure S24. Forest plot of dose-stratified traditional Meta-Analysis comparing efficacy of individual IL-23p19 inhibitor doses against placebo on clinical remission during maintenance.
Supplementary Figure S25. Forest plot of dose-stratified traditional Meta-Analysis comparing efficacy of individual IL-23p19 inhibitor doses against ustekinumab on clinical remission during maintenance.
Supplementary Figure S26. Forest plot of dose-stratified traditional Meta-Analysis comparing efficacy of individual IL-23p19 inhibitor doses against placebo on clinical response during maintenance.
Supplementary Figure S27. Forest plot of dose-stratified traditional Meta-Analysis comparing efficacy of individual IL-23p19 inhibitor doses against placebo on endoscopic remission during maintenance.
Supplementary Figure S28. Forest plot of dose-stratified traditional Meta-Analysis comparing efficacy of individual IL-23p19 inhibitor doses against ustekinumab on endoscopic remission during maintenance.
Supplementary Figure S29. Forest plot of dose-stratified traditional Meta-Analysis comparing efficacy of individual IL-23p19 inhibitor doses against placebo on endoscopic response during maintenance.
Supplementary Figure S30. Forest plot of dose-stratified traditional Meta-Analysis comparing efficacy of individual IL-23p19 inhibitor doses against ustekinumab on endoscopic response during maintenance.
Supplementary Figure S31. Forest plot of induction clinical remission by drug and assessment time point: a traditional Meta-Analysis stratified by induction duration.
Supplementary Figure S32. Forest plot of induction clinical response by drug and assessment time point: a traditional Meta-Analysis stratified by induction duration.
Supplementary Figure S33. Forest plot of induction endoscopic remission by drug and assessment time point: a traditional Meta-Analysis stratified by induction duration.
Supplementary Figure S34. Forest plot of induction endoscopic response by drug and assessment time point: a traditional Meta-Analysis stratified by induction duration.
Supplementary Figure S35. Risk of bias assessment.
Supplementary Figure S36. SUCRA cumulative rank curves of Sensitivity analysis for inducing the clinical remission among all participants, excluding ADVANCE.
Supplementary Figure S37. P-best histograms of Sensitivity analysis for inducing the clinical remission among all participants, excluding ADVANCE.
Supplementary Figure S38. SUCRA cumulative rank curves of Sensitivity analysis for inducing the clinical response among all participants, excluding ADVANCE.

Supplementary Figure S39. P-best histograms of Sensitivity analysis for inducing the clinical response among all participants, excluding ADVANCE.
Supplementary Figure S40. Network plot for clinical and endoscopic outcomes among previous bio-failure participants for induction phase.
Supplementary Figure S41. League table of interventions' efficacy on inducing and maintaining clinical outcomes with placebo as reference among previous bio-naive participants.
Supplementary Figure S42. League table of interventions' efficacy on inducing clinical outcomes with placebo as reference among previous bio-failure participants.
Supplementary Figure S43. League table of interventions' efficacy on inducing and maintaining clinical outcomes with placebo as reference among previous bio-naive participants.
Supplementary Figure S44. SUCRA cumulative rank curves of sensitivity analysis for maintaining the clinical remission among all participants, excluding SEQUENCE and CERTIFI.
Supplementary Figure S45.P-best histograms of sensitivity analysis for maintaining the clinical remission among all participants, excluding SEQUENCE and CERTIFI.
Supplementary Figure S46. SUCRA cumulative rank curves of sensitivity analysis for maintaining the clinical response among all participants, excluding CERTIFI.
Supplementary Figure S47.P-best histograms of sensitivity analysis for maintaining the clinical response among all participants, excluding CERTIFI.
Supplementary Figure S48. Network plot for clinical and endoscopic outcomes among previous bio-failure participants for maintenance phase.
Supplementary Figure S49. League table of interventions' efficacy on maintaining clinical outcomes with placebo as reference among previous bio-failure participants.
Supplementary Figure S50. SUCRA cumulative rank curves of Sensitivity analysis for maintaining the endoscopic remission among all participants, excluding FORTIFY.
Supplementary Figure S51.P-best histograms of Sensitivity analysis for maintaining the endoscopic remission among all participants, excluding FORTIFY.
Supplementary Figure S52. SUCRA cumulative rank curves of sensitivity analysis for maintaining the endoscopic response among all participants, excluding FORTIFY.
Supplementary Figure S53. P-best histograms of sensitivity analysis for maintaining the endoscopic response among all participants, excluding FORTIFY.
Supplementary Figure S54. SUCRA cumulative rank curves of different IL-23 and IL-12/23 agents for different efficacy on clinical and endoscopic outcomes during induction phase.
Supplementary Figure S55. SUCRA cumulative rank curves of different IL-23 and IL-12/23 agents for different efficacy on clinical and endoscopic outcomes during maintenance phase.
Supplementary Figure S56. P-best histograms of different IL-23 and IL-12/23 agents for different efficacy on clinical and endoscopic outcomes during induction phase.
Supplementary Figure S57. P-best histograms of different IL-23 and IL-12/23 agents for different efficacy on clinical and endoscopic outcomes during maintenance phase.
Supplementary Figure S58. Funnel chart of clinical and endoscopic outcomes during induction.
Supplementary Figure S59. Funnel chart of clinical and endoscopic outcomes during maintenance.
Supplementary Figure S60. League table of sensitivity analysis for comparative efficacy of different IL-23 and IL-12/23 agents on clinical remission and clinical response during induction phases, ADVANCE was excluded after sensitivity analysis.
Supplementary Figure S61. League table of sensitivity analysis for comparative efficacy of different IL-23 and IL-12/23 agents on clinical remission and clinical response during

maintenance phases. SEQUENCE and CERTIFI were excluded after sensitivity analysis for clinical remission in maintenance phase; CERTIFI was excluded after sensitivity analysis for clinical response in maintenance phase.

Supplementary Figure S62. League table of sensitivity analysis comparative efficacy of different IL-23 and IL-12/23 agents on endoscopic remission and endoscopic response during maintenance phase, FORTIFY was excluded after sensitivity analysis.

Reference list for supplementary materials

Supplementary Table S1. PRISMA 2020 Checklist.

Section and Topic	Item #	Checklist item	Location where item is reported
**TITLE**			
Title	1	Identify the report as a systematic review.	1
**ABSTRACT**			
Abstract	2	See the PRISMA 2020 for Abstracts checklist.	1
**INTRODUCTION**			
Rationale	3	Describe the rationale for the review in the context of existing knowledge.	1-2
Objectives	4	Provide an explicit statement of the objective(s) or question(s) the review addresses.	2
**METHODS**			
Eligibility criteria	5	Specify the inclusion and exclusion criteria for the review and how studies were grouped for the syntheses.	3
Information sources	6	Specify all databases, registers, websites, organisations, reference lists and other sources searched or consulted to identify studies. Specify the date when each source was last searched or consulted.	2
Search strategy	7	Present the full search strategies for all databases, registers and websites, including any filters and limits used.	Table S1
Selection process	8	Specify the methods used to decide whether a study met the inclusion criteria of the review, including how many reviewers screened each record and each report retrieved, whether they worked independently, and if applicable, details of automation tools used in the process.	3
Data collection process	9	Specify the methods used to collect data from reports, including how many reviewers collected data from each report, whether they worked independently, any processes for obtaining or confirming data from study investigators, and if applicable, details of automation tools used in the process.	3
Data items	10a	List and define all outcomes for which data were sought. Specify whether all results that were compatible with each outcome domain in each study were sought (e.g. for all measures, time points, analyses), and if not, the methods used to decide which results to collect.	3
	10b	List and define all other variables for which data were sought (e.g. participant and intervention characteristics, funding sources). Describe any assumptions made about any missing or unclear information.	3
Study risk of bias assessment	11	Specify the methods used to assess risk of bias in the included studies, including details of the tool(s) used, how many reviewers assessed each study and whether they worked independently, and if applicable, details of automation tools used in the process.	3
Effect measures	12	Specify for each outcome the effect measure(s) (e.g. risk ratio, mean difference) used in the synthesis or presentation of results.	4
Synthesis	13a	Describe the processes used to decide which studies were eligible for each synthesis (e.g. tabulating the study intervention	4

Section and Topic	Item #	Checklist item	Location where item is reported
methods		characteristics and comparing against the planned groups for each synthesis (item #5)).	
	13b	Describe any methods required to prepare the data for presentation or synthesis, such as handling of missing summary statistics, or data conversions.	4
	13c	Describe any methods used to tabulate or visually display results of individual studies and syntheses.	4
	13d	Describe any methods used to synthesize results and provide a rationale for the choice(s). If meta-analysis was performed, describe the model(s), method(s) to identify the presence and extent of statistical heterogeneity, and software package(s) used.	4
	13e	Describe any methods used to explore possible causes of heterogeneity among study results (e.g. subgroup analysis, meta-regression).	4
	13f	Describe any sensitivity analyses conducted to assess robustness of the synthesized results.	4
Reporting bias assessment	14	Describe any methods used to assess risk of bias due to missing results in a synthesis (arising from reporting biases).	3-4
Certainty assessment	15	Describe any methods used to assess certainty (or confidence) in the body of evidence for an outcome.	3-4
**RESULTS**			
Study selection	16a	Describe the results of the search and selection process, from the number of records identified in the search to the number of studies included in the review, ideally using a flow diagram.	4
	16b	Cite studies that might appear to meet the inclusion criteria, but which were excluded, and explain why they were excluded.	4
Study characteristics	17	Cite each included study and present its characteristics.	Table S2
Risk of bias in studies	18	Present assessments of risk of bias for each included study.	Table S6
Results of individual studies	19	For all outcomes, present, for each study: (a) summary statistics for each group (where appropriate) and (b) an effect estimate and its precision (e.g. confidence/credible interval), ideally using structured tables or plots.	Table S1
Results of syntheses	20a	For each synthesis, briefly summarise the characteristics and risk of bias among contributing studies.	Table S6
	20b	Present results of all statistical syntheses conducted. If meta-analysis was done, present for each the summary estimate and its precision (e.g. confidence/credible interval) and measures of statistical heterogeneity. If comparing groups, describe the direction of the effect.	4-13
	20c	Present results of all investigations of possible causes of heterogeneity among study results.	4-13
	20d	Present results of all sensitivity analyses conducted to assess the robustness of the synthesized results.	4-13

Section and Topic	Item #	Checklist item	Location where item is reported
Reporting biases	21	Present assessments of risk of bias due to missing results (arising from reporting biases) for each synthesis assessed.	4-13
Certainty of evidence	22	Present assessments of certainty (or confidence) in the body of evidence for each outcome assessed.	4-13
**DISCUSSION**			
Discussion	23a	Provide a general interpretation of the results in the context of other evidence.	13
	23b	Discuss any limitations of the evidence included in the review.	14-15
	23c	Discuss any limitations of the review processes used.	14-15
	23d	Discuss implications of the results for practice, policy, and future research.	14-15
**OTHER INFORMATION**			
Registration and protocol	24a	Provide registration information for the review, including register name and registration number, or state that the review was not registered.	2
	24b	Indicate where the review protocol can be accessed, or state that a protocol was not prepared.	2
	24c	Describe and explain any amendments to information provided at registration or in the protocol.	2
Support	25	Describe sources of financial or non-financial support for the review, and the role of the funders or sponsors in the review.	17
Competing interests	26	Declare any competing interests of review authors.	17
Availability of data, code and other materials	27	Report which of the following are publicly available and where they can be found: template data collection forms; data extracted from included studies; data used for all analyses; analytic code; any other materials used in the review.	17

From: Page MJ, McKenzie JE, Bossuyt PM, Boutron I, Hoffmann TC, Mulrow CD, et al. The PRISMA 2020 statement: an updated guideline for reporting systematic reviews. BMJ 2021;372:n71. doi: 10.1136/bmj.n71. This work is licensed under CC BY 4.0. To view a copy of this license, visit https://creativecommons.org/licenses/by/4.0/

Supplementary Table S2. Detailed search strategies.

PubMed (Search Date: 2025-11-04)		
Number	Searches	Results
#1	"crohn disease"[MeSH] OR "crohn s disease"[Title/Abstract] OR "crohns disease"[Title/Abstract] OR "crohn s enteritis"[Title/Abstract] OR "inflammatory bowel disease 1"[Title/Abstract] OR "regional enteritis"[Title/Abstract] OR "Ileocolitis"[Title/Abstract] OR "ileitis terminal"[Title/Abstract] OR "terminal ileitis"[Title/Abstract] OR "ileitis regional"[Title/Abstract] OR "regional ileitis"[Title/Abstract] OR "enteritis granulomatous"[Title/Abstract] OR "granulomatous enteritis"[Title/Abstract] OR "enteritis regional"[Title/Abstract] OR "colitis granulomatous" [Title/Abstract] OR "granulomatous colitis"[Title/Abstract]	71885
#2	"risankizumab"[Title/Abstract] OR "BI-655066"[Title/Abstract] OR "BI-655066"[Title/Abstract] OR "skyrizi"[Title/Abstract] OR "risankizumab-rzaa"[Title/Abstract] OR "ABBV-066"[Title/Abstract]	774
#3	"Mirikizumab"[Title/Abstract] OR "LY-3074828"[Title/Abstract] OR "LY3074828"[Title/Abstract]	172
#4	"guselkumab"[Title/Abstract] OR "Tremfya"[Title/Abstract] OR "CNTO-1959"[Title/Abstract] OR "CNTO-1959"[Title/Abstract]	953
#5	"Interleukin-23"[MeSH] OR "Interleukin-23"[Title/Abstract] OR "IL-23"[Title/Abstract]	11314
#6	"Ustekinumab"[MeSH] OR "CNTO-1275"[Title/Abstract] OR "CNTO-1275"[Title/Abstract] OR "Stelara"[Title/Abstract]	2202
#7	#2 OR #3 OR #4 OR #5 OR #6	13875
#8	#1 AND #7	1352
#9	#8 NOT ((((((case reports[Publication Type]) OR (comment[Publication Type])) OR (editorial[Publication Type])) OR (letter[Publication Type])) OR (review[Publication Type])) OR (guideline[Publication Type]))	793

Embase (Search Date: 2025-11-04)		
Number	Searches	Results
#1	'Crohn disease'/exp	133559
#2	'cleron disease':ab,ti OR 'crohn`s disease':ab,ti OR 'crohns disease':ab,ti OR 'enteritis regionalis':ab,ti OR 'intestinal tract, regional enteritis':ab,ti OR 'morbus crohn':ab,ti OR 'regional enteritis':ab,ti OR 'regional enterocolitis':ab,ti OR 'crohn disease':ab,ti	109495
#3	#1 OR #2	143959
#4	'risankizumab'/exp	3138
#5	'abbv 066':ab,ti OR 'abbv066':ab,ti OR 'bi 655066':ab,ti OR 'bi655066':ab,ti OR 'risankizumab rzaa':ab,ti OR 'risankizumab-rzaa':ab,ti OR 'skyrizi':ab,ti OR 'risankizumab':ab,ti	1542
#6	#4 OR #5	3194
#7	'mirikizumab'/exp	766

#8	'ly 3074828':ab,ti OR 'ly3074828':ab,ti OR 'mirikizumab mrkz':ab,ti OR 'mirikizumab-mrkz':ab,ti OR 'omvoh':ab,ti OR 'mirikizumab':ab,ti	542
#9	#7 OR #8	779
#10	'guselkumab'/exp	3809
#11	'cnto 1959':ab,ti OR 'cnto1959':ab,ti OR 'tremfya':ab,ti OR 'guselkumab':ab,ti	1986
#12	#10 OR #11	3871
#13	'Interleukin-23':ab,ti OR 'Interleukin 23':ab,ti OR 'IL-23':ab,ti	15610
#14	'ustekinumab'/exp	17405
#15	'abp 654':ab,ti OR 'abp654':ab,ti OR 'absimky':ab,ti OR 'amg 654':ab,ti OR 'amg654':ab,ti OR 'avt 04':ab,ti OR 'avt04':ab,ti OR 'bat 2206':ab,ti OR 'bat2206':ab,ti OR 'bfi 751':ab,ti OR 'bfi751':ab,ti OR 'bmab 1200':ab,ti OR 'bmab1200':ab,ti OR 'bow 090':ab,ti OR 'bow090':ab,ti OR 'cnto 1275':ab,ti OR 'cnto1275':ab,ti OR 'ct p43':ab,ti OR 'ctp43':ab,ti OR 'da 3115':ab,ti OR 'da3115':ab,ti OR 'dmb 3115':ab,ti OR 'dmb3115':ab,ti OR 'eb 1004':ab,ti OR 'eb1004':ab,ti OR 'eksunbi':ab,ti OR 'fyb 202':ab,ti OR 'fyb202':ab,ti OR 'fymskina':ab,ti OR 'imuldosa':ab,ti OR 'jamteki':ab,ti OR 'monoclonal antibody cnto 1275':ab,ti OR 'ons 3040':ab,ti OR 'ons3040':ab,ti OR 'otulfi':ab,ti OR 'pb 007':ab,ti OR 'pb007':ab,ti OR 'pyzchiva':ab,ti OR 'ro 723 3920':ab,ti OR 'ro 7233920':ab,ti OR 'ro7233920':ab,ti OR 'sb 17':ab,ti OR 'sb17':ab,ti OR 'selarsdi':ab,ti OR 'stelara':ab,ti OR 'stellara':ab,ti OR 'steqeyma':ab,ti OR 'suterara':ab,ti OR 'tt 20':ab,ti OR 'tt20':ab,ti OR 'upstelda':ab,ti OR 'ustekinumab aauz':ab,ti OR 'ustekinumab aekn':ab,ti OR 'ustekinumab auub':ab,ti OR 'ustekinumab srlf':ab,ti OR 'ustekinumab ttwe':ab,ti OR 'ustekinumab-aauz':ab,ti OR 'ustekinumab-aekn':ab,ti OR 'ustekinumab-auub':ab,ti OR 'ustekinumab-srlf':ab,ti OR 'ustekinumab-ttwe':ab,ti OR 'uzpruvo':ab,ti OR 'wezenla':ab,ti OR 'wezlana':ab,ti OR 'ustekinumab':ab,ti	9437
#16	#14 OR #15	17839
#17	#6 OR #9 OR #12 OR #13 OR #16	33714
#18	#3 AND #17	7455
#19	#18 AND ([editorial]/lim OR [letter]/lim OR [note]/lim OR [review]/lim OR [conference abstract]/lim OR [conference review]/lim)	4370

Web of Science (Search Date: 2025-11-04)		
Number	Searches	Results
#1	TS=(crohn disease OR crohn s disease OR crohns disease OR crohn s enteritis OR inflammatory bowel disease 1 OR regional enteritis OR Ileocolitis OR ileitis terminal OR terminal ileitis OR ileitis regional OR regional ileitis OR enteritis granulomatous OR granulomatous enteritis OR enteritis regional OR colitis granulomatous OR granulomatous colitis)	90760
#2	TS=(risankizumab OR BI-655066 OR skyrizi OR risankizumab-rzaa OR ABBV-066)	1224

#3	TS=(Mirikizumab OR LY-3074828 OR LY3074828)	480
#4	TS=(guselkumab OR Tremfya OR CNTO-1959)	1716
#5	TS=(Interleukin-23 OR IL-23)	11002
#6	TS=(Ustekinumab OR CNTO-1275 OR Stelara)	6256
#7	#2 OR #3 OR #4 OR #5 OR #6	18391
#8	#1 AND #7	3687
#9	#8 and Article (Document Types)	1713

Cochrane (Search Date: 2025-11-04)		
Number	Searches	Results
#1	MeSH descriptor: [Crohn Disease] explode all trees	2394
#2	(Crohn Disease):ab,ti,kw OR (Crohn's Disease):ab,ti,kw OR(Crohns Disease):ab,ti,kw OR(Crohn's Enteritis):ab,ti,kw OR(Inflammatory Bowel Disease 1):ab,ti,kw OR(Regional Enteritis):ab,ti,kw OR(Ileocolitis):ab,ti,kw OR(Ileitis, Terminal):ab,ti,kw OR(Terminal Ileitis):ab,ti,kw OR(Ileitis, Regional):ab,ti,kw OR(Regional Ileitides):ab,ti,kw OR(Regional Ileitis):ab,ti,kw OR(Enteritis, Granulomatous):ab,ti,kw OR(Granulomatous Enteritis):ab,ti,kw OR(Enteritis, Regional):ab,ti,kw OR(Colitis, Granulomatous):ab,ti,kw OR(Granulomatous Colitis):ab,ti,kw	8919
#3	#1 OR #2	8919
#4	MeSH descriptor: [Interleukin-23] explode all trees	165
#5	(Interleukin-23):ab,ti,kw OR (Interleukin 23):ab,ti,kw OR (IL-23):ab,ti,kw	3415
#6	#4 OR #5	3415
#7	MeSH descriptor: [Ustekinumab] explode all trees	356
#8	(Ustekinumab):ab,ti,kw OR (CNTO 1275):ab,ti,kw OR (CNTO-1275):ab,ti,kw OR (Stelara):ab,ti,kw	1351
#9	#7 OR #8	1351
#10	(risankizumab):ab,ti,kw OR (BI 655066):ab,ti,kw OR (BI-655066):ab,ti,kw OR (skyrizi):ab,ti,kw OR (risankizumab-rzaa):ab,ti,kw OR (ABBV-066):ab,ti,kw	416
#11	(mirikizumab):ab,ti,kw OR (LY-3074828):ab,ti,kw OR (LY3074828):ab,ti,kw	315
#12	(guselkumab):ab,ti,kw OR (Tremfya):ab,ti,kw OR (CNTO 1959):ab,ti,kw OR (CNTO-1959):ab,ti,kw	793
#13	#6 OR #9 OR #10 OR #11 OR #12	5267
#14	#3 AND #13 in Trials	839

Supplementary Table S3. Stratification of treatment arms for nodes in Network meta-analysis.

Induction

Trial	Node	Treatment arm	Sample (n)
SEQUENCE	RZB≤600mg	Risankizumab 600mg iv (weeks 0, 4, 8)	255
SEQUENCE	UST 6mg/kg	Ustekinumab 6 mg/kg iv single dose	265
ADVANCE	RZB≤600mg	Risankizumab 600mg iv (weeks 0, 4, 8)	336
ADVANCE	RZB>600mg	Risankizumab 1200mg iv (weeks 0, 4, 8)	339
ADVANCE	PBO	Placebo	175
MOTIVATE	RZB≤600mg	Risankizumab 600mg iv (weeks 0, 4, 8)	191
MOTIVATE	RZB>600mg	Risankizumab 1200mg iv (weeks 0, 4, 8)	191
MOTIVATE	PBO	Placebo	187
M15-993	RZB≤600mg	Risankizumab 200mg iv (weeks 0, 4, 8)	41
M15-993	RZB≤600mg	Risankizumab 600mg iv (weeks 0, 4, 8)	41
M15-993	PBO	Placebo	39
VIVID-1	MRK>600mg	Mirikizumab 900mg iv (week 0, 4, 8)	579
VIVID-1	PBO	Placebo	199
SERENITY	MRK≤600mg	Mirikizumab 200mg iv (weeks 0, 4, 8)	31
SERENITY	MRK≤600mg	Mirikizumab 600mg iv (weeks 0, 4, 8)	32
SERENITY	MRK>600mg	Mirikizumab 1000mg iv (weeks 0, 4, 8)	64
SERENITY	PBO	Placebo	64
GALAXI-1	GUS<600mg	Guselkumab 200mg iv (weeks 0, 4, 8)	61
GALAXI-1	GUS<600mg	Guselkumab 600mg iv (weeks 0, 4, 8)	63
GALAXI-1	GUS≥600mg	Guselkumab 1200mg iv (weeks 0, 4, 8)	61
GALAXI-1	UST 6mg/kg	Ustekinumab 6mg/kg iv single dose	63
GALAXI-1	PBO	Placebo	61
GALAXI-2,3	GUS<600mg	Guselkumab 200mg iv (weeks 0,4,8)	582
GALAXI-2,3	PBO	Placebo	148
GRAVITY	GUS<600mg	Guselkumab 400mg sc (weeks 0,4,8)	230
GRAVITY	PBO	Placebo	117
UNITI-1	UST<6mg/kg	Ustekinumab 130mg iv single dose	245
UNITI-1	UST 6mg/kg	Ustekinumab 6mg/kg iv single dose	249
UNITI-1	PBO	Placebo	247
UNITI-2	UST<6mg/kg	Ustekinumab 130mg iv single dose	209
UNITI-2	UST 6mg/kg	Ustekinumab 6mg/kg iv single dose	209
UNITI-2	PBO	Placebo	210
CERTIFI	UST<6mg/kg	Ustekinumab 1mg/kg iv single dose	131
CERTIFI	UST<6mg/kg	Ustekinumab 3mg/kg iv single dose	132
CERTIFI	UST 6mg/kg	Ustekinumab 6mg/kg iv single dose	131
CERTIFI	PBO	Placebo	132

RZB, Risankizumab, MRK, Mirikizumab, GUS, Guselkumab, UST, Ustekinumab, PBO, placebo, iv, intravenous, sc, subcutaneous, q4w, every 4 weeks.

Maintenance

Trial	Node	Treatment arm	Sample (n)
SEQUENCE	RZB	Risankizumab 360mg sc q8w	255
SEQUENCE	UST	Ustekinumab 90mg sc q8w	265
FORTIFY	RZB	Risankizumab 180mg sc q8w	157
FORTIFY	RZB	Risankizumab 360mg sc q8w	141
FORTIFY	PBO	Placebo	164
VIVID-1	MRK	Mirikizumab 300mg sc q4w	579
VIVID-1	UST	Ustekinumab 90mg sc q8w	287
VIVID-1	PBO	Placebo	199
GALAXI-1	GUS 100mg	Guselkumab 200mg-100mg sc q8w	61
GALAXI-1	GUS 200mg	Guselkumab 600mg-200mg sc q4w	63
GALAXI-1	GUS 200mg	Guselkumab 1200mg-200mg sc q4w	61
GALAXI-1	UST	Ustekinumab 90mg sc q8w	63
GALAXI-2,3	GUS 100mg	Guselkumab 100mg sc q8w	296
GALAXI-2,3	GUS 200mg	Guselkumab 200mg sc q4w	299
GALAXI-2,3	UST	Ustekinumab 90mg sc q8w	300
GALAXI-2,3	PBO	Placebo	153
GRAVITY	GUS 100mg	Guselkumab 100mg sc q8w	115
GRAVITY	GUS 200mg	Guselkumab 200mg sc q4w	115
GRAVITY	PBO	Placebo	117
IM-UNITI	UST	Ustekinumab 90mg sc q8w	132
IM-UNITI	UST	Ustekinumab 90mg sc q12w	132
IM-UNITI	PBO	Placebo	133
CERTIFI	UST	Ustekinumab 90mg sc q8w (weeks 8, 16)	72
CERTIFI	PBO	Placebo	73

RZB, Risankizumab, MRK, Mirikizumab, GUS, Guselkumab, UST, Ustekinumab, PBO, placebo, iv, intravenous, sc, subcutaneous, q4w, every 4 weeks, q8w, every 8 weeks, q12w, every 12 weeks.

Reationales for arms stratifications

In this network meta-analysis (NMA) of this study, the induction dose of risankizumab (RZB) was divided into two nodes: "RZB≤600mg" (600 mg iv q4w and 200mg iv q4w) and "RZB >600 mg" (1200 mg iv q4w). The clinical and endoscopic response rate was numerically greater at 600 mg than 200 mg or placebo; however not statistically significant [12]. Subjects treated with risankizumab showed numerically better endoscopic response rate at both 200 mg and 600 mg doses, compared to placebo[14], which suggests the dose-response curve has not yet reached a clear plateau. To verify the robustness of this grouping strategy, we also conducted a sensitivity analysis excluding the 200 mg RZB subgroup, as presented in the appendix (Supplementary Figure 43 and 44). During the maintenance period, Risankizumab 180 mg and 360 mg demonstrated highly overlapping rates of clinical remission, endoscopic response, and biomarker improvements in the pivotal Phase III FORTIFY maintenance trial, with no dose-dependent differences observed in the safety profile[2]. Both 180 mg and 360 mg are considered effective; no clear dose-response was observed. The 360 mg dose may confer numerically higher endoscopic remission, but the incremental benefit is modest [14]. Therefore, the two doses were combined into a single "RZB" node to avoid network fragmentation and improve estimation accuracy.

The induction dose of Mirikizumab (MRK) was categorized into "MRK ≤600 mg"(including 200mg iv q4w and 600mg IV q4w) and "MRK >600 mg"(900mg iv q4w and 1000mg iv q4w) groups in our network meta-analysis. This stratification was guided by the following key evidence-based rationales. A model-based exposure–efficacy analysis indicated that the 900 mg iv of mirikizumab in VIVID-1 produced efficacy comparable to the 1000 mg IV regimen evaluated in SERENITY, regardless of prior biologic failure [15]. Intravenous doses of 600-1000mg of mirikizumab have been proven to demonstrate near-maximal efficacy by 12 weeks [15]. From a statistical modeling perspective, treating 200 mg and 600 mg as separate nodes would result in network sparsity and insufficient event counts. In the maintenance phase, the MRK regimen employed only the 300 mg dose from VIVID-1 [5], which precluded any grouping or pooling of dose arms.

We stratified the guselkumab induction regimen into two dose tiers: the “GUS<600mg” group (including 200 mg iv q4w and 400 mg sc q4w) and the “GUS≥600 mg” group (including 600mg iv q4w and 1200mg iv q4w). Guselkumab for treating Crohn’s disease was given as an induction course of 200 mg intravenously at weeks 0, 4, and 8, or as 400 mg subcutaneously at the same time-points [16]. Although the 200 mg iv and 400 mg sc doses differ in their route of administration, pharmacokinetic data from the prescribing information indicate that their systemic exposure (AUC) is comparable at the end of the induction period [16]. Since the 600 mg and 1200 mg doses were not designated as recommended dosages but belonged to investigational high-dose groups, they were assigned to a separate category to differentiate them from the standard therapeutic doses [7] [16]. For the maintenance phase, although the total dose administered over an 8-week weeks is equivalent (200 mg), the two maintenance regimens of guselkumab (100 mg every 8 weeks vs. 200 mg every 4 weeks) result in markedly different steady-state trough concentrations (1.2 vs. 10.1 mcg/mL) [16]. Furthermore, the FDA label recommends using the lowest effective dose, implying that these regimens are not presumed equivalent in clinical practice[16]. Therefore, we preserved both GUS 100 mg q8w and GUS 200 mg q4w as independent nodes in our NMA.

The induction dose of ustekinumab (UST) was divided into two groups: "UST<6 mg/kg" (including 1mg/kg, 3mg/kg iv and 1iv30mg sc) and "UST 6 mg/kg"(6mg/kg iv). Pharmacokinetic (PK) data indicated that a 6 mg/kg intravenous dose, which was recommended as an induction dose by the FDA, rapidly achieved the target trough concentration associated with long-term remission. To maintain network connectivity, the fixed-dose Phase II arm (e.g., 130 mg sc) [10], which could not be precisely stratified by weight (mg/kg), was incorporated into the "<6 mg/kg" stratum after conversion using an average 70 kg body weight. As for maintenance dose, Ustekinumab 90 mg SC administered at both

q8w and q12w intervals was pooled and defined as a unified "UST" node in the network. The two treatment groups showed consistent efficacy with ustekinumab across all prespecified subgroups [10]. The FDA label treats both intervals as the same 90 mg dose strength, merely recommending q8w as the default without declaring q12w sub-therapeutic [17]. If dose stratification is retained, self-referential loops may emerge in the network, increasing the risk of inconsistency.

In conclusion, grouping of treatment arms in this meta-analysis not only conformed to the dose-exposure-effect relationship, but also met the best practice of network meta-analysis to deal with dose heterogeneity, while preserving the potential dose-effect information.

Supplementary Table S4. Characteristics of included trails.

Trails	Author Year*	Phase	Center	Groups, n	Duration	Age, yr (mean(SD)/median (range))	Gender ,n male/female	Disease location Ileum/ colon/ ileocolonic, %	Steroids use, %	Immunosuppressants use, %	Prior biologics/anti-TNF/anti-integrin failure , %
SEQUENCE [1]<br>NCT04524611	Peyrin-Biroulet<br>2024	IIIb	187 sites in 28 countries	RZB 600/360mg IV: 255<br>UST 90mg SC: 265<br>Total: 520	Induction<br>Maintenance	RZB:38.0(13.1)<br>UST:38.3(13.8)	267/253	RZB:16.5/40/43.5<br>UST:17/40/43	RZB:22.7<br>UST:26.8	RZB:13.3<br>UST:17.7	RZB:NA/100/NA<br>UST:NA/100/NA
FORTIFY [2]<br>NCT03105102	Ferrante<br>2022	III	273 sites in 44 countries	RZB(I) 180mg IV:157<br>RZB(II) 360mg IV:141<br>PBO:164<br>Total: 462	Maintenance	RZB (I) 39.1(14.8)<br>RZB (II)37.0 (12.8)<br>PBO 38.0 (13.0)	238/224	RZB(I):10/45/46<br>RZB(II):11/42/48<br>PBO:14/38/48	RZB(I):32<br>RZB(II):30<br>PBO:31	RZB(I):26<br>RZB(II):28<br>PBO:24	RZB(I):72/68/NA<br>RZB(II):72/64/NA<br>PBO:75/73/NA
ADVANCE [3]<br>NCT03105128	D'Haens<br>2022	III	297 sites in 39 countries	RZB(I) 600mg IV:336<br>RZB(II) 1200mg IV:339<br>PBO:175<br>Total 850	Induction	RZB (I)38.3 (13.3)<br>RZB (II)37.0 (13.2)<br>PBO 37.1 (13.4)	460/390	RZB (I):15/34/50<br>RZB(II):16/35/49<br>PBO:11/40/49	RZB (I):30<br>RZB (II):30<br>PBO:29	RZB(I):26<br>RZB(II):22<br>PBO:24	RZB(I):58/55/NA<br>RZB(II):59/55/NA<br>PBO:55/52/NA
MOTIVATE [3]<br>NCT03104413	D'Haens<br>2022	III	214 sites in 40 countries	RZB(I) 600mg IV:191<br>RZB(II) 1200mg IV:191<br>PBO:187<br>Total: 569	Induction	RZB (I)40.2 (13.6)<br>RZB (II)39.3(12.9)<br>PBO 39.3 (13.5)<br>39.6 (13.3)	293/276	RZB(I):17/39/43<br>RZB(II):11/39/50<br>PBO:14/39/47	RZB(I):34<br>RZB(II):32<br>PBO:36	RZB(I):19<br>RZB(II):28<br>PBO:21	RZB(I):100/93/NA<br>RZB(II):100/95/NA<br>PBO:100/97/NA
M15-993 [4]<br>NCT02031276	Feagan<br>2018	II	36 sites (Europe, southeast Asia, North America)	RZB(I) 200mg IV: 41<br>RZB(II) 600mg IV: 41<br>PBO: 39<br>Total:121	Induction	RZB(I):39(13)<br>RZB(II):40(13)<br>PBO: 36(14)	47/74	RZB(I):15/24/61<br>RZB(II):34/39/10<br>PBO: 46/41/9	RZB(I):24<br>RZB(II):29<br>PBO:28	RZB(I):24<br>RZB(II):20<br>PBO:33	RZB(I):NA/90/NA<br>RZB(II):NA/88/NA<br>PBO:NA/90/NA
VIVID-1 [5]<br>NCT03926130	Jairath<br>2024	III	324 sites in 33 countries	MRK 900/300mg : 579<br>UST ~6mg/kg/90mg: 287<br>PBO: 199<br>Total 1065	Induction<br>Maintenance	MRK:36(13.2)<br>UST:36.6(12.7)<br>PBO:36.3(12.7)	587/478	MRK:11.2/38.9/49.9<br>UST:10.1/41.8/48.1<br>PBO:9.5/38.7/51.8	MRK:30.6<br>UST:31.4<br>PBO:29.1	MRK:25.2<br>UST:30.3<br>PBO:29.1	MRK:48.5/45.8/11.7<br>UST:48.4/46.3/10.8<br>PBO:48.7/44.7/12.1
SERENITY [6]<br>NCT02891226	Sands<br>2021	II	80 sites in 14 countries	MRK(I) 200mg IV:31<br>MRK(II) 600mg IV:32<br>MRK(III) 1000mg IV:64<br>PBO:64<br>Total:191	Induction<br>Maintenance	MRK(I): 38.1(11.8)<br>MRK(II):40.4(13.3)<br>MRK(III):37.7(13.1)<br>PBO:39.0(13.0)	93/98	MRK(I):19.4/45.2/35.5<br>MRK(II):15.6/31.3/53.1<br>MRK(III):17.2/40.6/42.2/6.7?<br>PBO:17.2/39.1/43.8	MRK(I):45.2<br>MRK(II):21.9<br>MRK(III):23.4<br>PBO:32.8	MRK(I):38.7<br>MRK(II):31.3<br>MRK(III):32.8<br>PBO:29.7	MRK(I):48.4/NA/16.1<br>MRK(II):50/NA/15.6<br>MRK(III):48.4/NA/9.4<br>PBO:56.3/NA/21.9
GALAXI 1 [7]<br>NCT03466411	Danese<br>2024	II	128 sites in 32 conutries	GUS(I) 200mg IV: 61<br>GUS(II) 600mg IV: 63<br>GUS(III) 1200mg IV: 61<br>UST: 63<br>PBO: 61	Induction	GUS:39.6 (13.9)<br>GUS(I):40.3(13.7)<br>GUS(II):39(14.4)<br>GUS(III):39.6(13.7)<br>UST:36.1(12.0)	183/126	GUS(I):28/44/28<br>GUS(II):35/29/37<br>GUS(III):21/51/28<br>UST:17/46/37<br>PBO:18/43/39	GUS(I):39<br>GUS(II):30<br>GUS(III):33<br>UST:41<br>PBO:39	GUS(I):25<br>GUS(II):29<br>GUS(III):41<br>UST:41<br>PBO:43	GUS(I):52.5/49.2/9.8<br>GUS(II):55.6/55.6/12.7<br>GUS(III):55.7/54.1/4.9<br>UST:58.7/58.7/7.9<br>PBO:49.2/47.5/8.2

				Total: 309		PBO:38.9(13.0)				
GALAXI 2, 3**<br>[8]<br>NCT03466411	Panaccione<br>2024	III	257 sites<br>in 40<br>countries	GUS (I) 200/100mg: 286<br>GUS (II) 200/200mg: 296<br>UST: 291<br>PBO: 148<br>Total: 1021	Induction<br>Maintenance	GUS(I):36.0 (12.2)<br>GUS(II):36.9(13.3)<br>UST:37.4 (13.2)<br>PBO:34.8 (12.2)	590/431	GUS(I):20.8/41.7/40.3<br>GUS(II):27.0/37.8/35.1<br>UST:18.9/39.9/41.2<br>PBO:20.9/41.9/37.2	GUS(I): 38.5<br>GUS(II):35.8<br>UST:37.5<br>PBO:41.2	GUS(I):NA<br>GUS(II):NA<br>UST:NA<br>PBO:NA	GUS(I):54.1/52.7/8.8<br>GUS(II):49.7/48.3/6.1<br>UST:53.6/50.5/10.7<br>PBO:52.7/51.4/8.8
GRAVITI [9]<br>NCT05197049	Hart<br>2025	III	143 sites<br>in 23<br>countries	GUS(I) 400/100mg SC:115<br>GUS(II) 400/200mg SC:115<br>PBO:117<br>Total: 347	Induction<br>Maintenance	GUS(I):37.4(13.3)<br>GUS(II):39.0(12.6)<br>PBO:36.0(12.7)	203/144	GUS(I):21.7/35.7/42.6<br>GUS(II):23.5/34.8/41.7<br>PBO:18.8/34.3/47	GUS(I):27.8<br>GUS(II):33.0<br>PBO:28.2	GUS(I): 24.3<br>GUS(II):31.3<br>PBO:27.4	GUS(I):47.8/44.3/11.3<br>GUS(II):46.1/45.2/5.2<br>PBO:45.3/42.7/6.8
UNITI-1 [10]<br>NCT01369329	Feagon<br>2016	III	178 sites<br>in 23<br>countries	UST(I) 130mg IV:245<br>UST(II) 6mg/kg IV:249<br>PBO:247<br>Total: 741	Induction	UST(I):37.4 (11.8)<br>UST(II):37.3 (12.5)<br>PBO:37.3 (11.8)	317/424	UST(I):15.5/14.7/69.8<br>UST(II):14.9/16.1/68.7<br>PBO:11.4/19.5/67.5	UST(I):49.4<br>UST(II):43.4<br>PBO:44.9	UST(I):30.2<br>UST(II):31.3<br>PBO:32.8	UST(I): NA/99.2/NA<br>UST(II):NA/98.8/NA<br>PBO:NA/99.6/NA
UNITI-2 [10]<br>NCT01369342	Feagon<br>2016	III	175 sites<br>in 23<br>countries	UST(I) 130mg IV:209<br>UST(II) 6mg/kg IV:209<br>PBO:210<br>Total: 628	Induction	UST (I) 39.1 (13.8)<br>UST (II) 38.4(13.1)<br>PBO 40.2 (13.1)	293/335	UST(I):25.5/21.2/52.4<br>UST(II):23.4/20.6/56<br>PBO:21/17.6/61.4	UST(I):38.3<br>UST(II):44<br>PBO:35.7	UST(I):35.4<br>UST(II):34.4<br>PBO:34.8	UST(I):NA/NA/NA<br>UST(II):NA/NA/NA<br>PBO:NA/NA/NA
IM-UNITI [10]<br>NCT01369355	Feagon<br>2016	III	260 sites<br>in 27<br>countries	UST(I) 90mg SC q12w:132<br>UST(II) 90mg SC q8w:132<br>PBO:133<br>Total: 397	Maintenance	UST(I):37.9 (13.2)<br>UST(II):38.6 (13.7)<br>PBO:39.5(12.7)	173/224	UST(I):19.7/17.4/62.9<br>UST(II):14.4/22/63.6<br>PBO:14.3/21.1/64.7	UST(I):43.9<br>UST(II):48.5<br>PBO:44.4	UST(I):39.4<br>UST(II):33.3<br>PBO:35.3	UST(I):NA/44.7/NA<br>UST(II):NA/43.9/NA<br>PBO:NA/45.9/NA
CERTIFI [11]<br>NCT00771667	Sandborn<br>2012	IIb	153 sites<br>in 12<br>countries	UST(I) 1mg/kg IV:131<br>UST(II) 3mg/kg IV:132<br>UST(III) 6mg/kg IV:131<br>PBO:132<br>Total:526	Induction<br>Maintenance	UST(I):38.8(12.0)<br>UST(II):38.2(12.6)<br>UST(III):39.4(13.2)<br>PBO:39.5(13.1)	217/309	UST(I):NA<br>UST(II):NA<br>UST(III):NA<br>PBO:NA	UST(I):45.0<br>UST(II):53.8<br>UST(III):45.0<br>PBO:55.3	UST(I):25.2<br>UST(II):21.2<br>UST(III):26.7<br>PBO:22.7	UST(I):NA/100/NA<br>UST(II):NA/100/NA<br>UST(III):NA/99.2/NA<br>PBO:NA/100/NA

*Author and year are based on the first author and publication year from the primary publication of each trail.
**pooled GALAXI 2 and 3 data
Abbreviations: BL baseline, CDAI Crohn's Disease Activity Index, SD standard SES-CD Simple Endoscopic Score for Crohn's Disease, SF/APS, stool frequency or abdominal pain score, RZB risankizumab, MRK mirikizumab, GUS guselkumab, UST ustekinumab, PBO placebo, NA, not available, SC, subcutaneous, IV, intravenous, yr, year, TNF, tumor

Supplementary Table S5. Definition of clinical and endoscopic outcomes from included trails.

Trails	Author Year*	CDAI clinical remission	endoscopic remission	PRO clinical remission	CDAI clinical response	endoscopic response	PRO clinical response	deep remission	mucosal healing	IBDQ improvement	biologic improvement	Co-endpoint
SEQUENCE [1] NCT04524611	Peyrin-Biroulet 2024	CDAI<150 [8, 24, 48w]	SES-CD≤ 4 and at least a 2-point reduction versus BL and no subscore >1 [24, 48w]	SF ≤ 2.8 and AP ≤ 1 and not worse than BL [8, 24, 48w]	Reduction of CDAI ≥100 from BL [8, ,24, 48w]	decrease in SES-CD >50% from BL [or for patients with a BL SES-CD of 4, at least a 2-point reduction from BL] [24, 48w]	NA	clinical remission and endoscopic remission [24, 48w]	SES-CD ulcerated surface subscore of 0 which ≥1 at BL [24, 48w]	NA (mean change from BL [24, 48w])	remission: clinical remission and FCP ≤250 mg/kg or hs-CRP ≤5 mg/L [8, 24, 48w]	NA
FORTIFY [2] NCT03105102	Ferrante 2022	CDAI<150 [52w]	SES-CD≤4 and at least a 2-point reduction v.s. BL and no subscore >1 [52w]	SF ≤ 2.8 and AP ≤ 1 and not worse than BL[52w]	Reduction of CDAI ≥100 from BL [52w]	decrease in SES-CD >50% from BL[or for patients with isolated ileal disease and a BL SES-CD of 4, at least a 2-point reduction from BL [52w]	≥ 30% decrease in SF and/or ≥ in AP score and both not worse than BL [52w]	clinical (CDAI or PRO) and endoscopic remission[52w]	SES-CD ulcerated surface subscore of 0 which ≥1 at BL [52w]	NA	NA	52w clinical remission + 52w endoscopic response; 52w PRO remission + 52w endoscopic response
ADVANCE [3] NCT03105128	D'Haens 2022	CDAI<150 [12w]	SES-CD≤4 and at least a 2-point reduction versus BL and no subscore >1 [12w]	SF ≤ 2.8 and AP ≤ 1 and not worse than BL [12w]	Reduction of CDAI ≥100 from BL [12w]	decrease in SES-CD >50% from BL[or for patients with isolated ileal disease and a BL SES-CD of 4, at least a 2-point reduction from BL] [12w]	≥ 30% decrease in SF and/or ≥ in AP score and both not worse than BL [12w]	NA	SES-CD ulcerated surface subscore of 0 which ≥1 at BL [12w]	NA	NA	12w clinical response + 12w endoscopic response; 12w enhanced PRO response(≥60% decrease SF or ≥35% decrease in AP neither worse than BL or PRO remission) + 12w endoscopic response
MOTIVATE [3] NCT03104413	D'Haens 2022	CDAI<150 [12w]	SES-CD≤4 and at least a 2-point reduction versus BL and no subscore >1 [12w]	SF ≤ 2.8 and AP ≤ 1 and not worse than BL [12w]	Reduction of CDAI ≥100 from BL [12w]	decrease in SES-CD >50% from BL[or for patients with isolated ileal disease and a BL SES-CD of 4, at least a 2-point reduction from BL] [12w]	≥ 30% decrease in SF and/or ≥ in AP score and both not worse than BL[12w]	NA	SES-CD ulcerated surface subscore of 0 which ≥1 at BL [12w]	NA	NA	12w clinical response + 12w endoscopic response; 12w enhanced PRO response(≥60% decrease SF or ≥35% decrease in AP neither worse than BL or PRO remission) + 12w endoscopic response
M15-993 [4] NCT02031276	Feagan 2018	CDAI<150 [12w]	CDEIS ≤4 or ≤2 for patients with isolated ileitis [12w]	NA	CDAI <150 or a CDAI reduction from BL of ≥100[12w]	>50% CDEIS reduction from BL [12w]	NA	clinical remission and endoscopic remission [12w]	absence of mucosal ulceration [12w]	remission:IBDQ≥170; response: IBDQ 16-point increase from BL [12w]	NA (median change from BL [24w])	NA
VIVID-1 [5] NCT03926130	Jairath 2024	CDAI<150 [12, 52w]	SES-CD ≤4 and at least a 2-point reduction versus BL and no subscore >1 [12, 52w]	SF ≤ 3 and AP ≤ 1 and no worse than BL [52w]	NA	decrease in SES-CD >50% from BL [12, 52w]	≥30% decrease in AP and/or SF and no worse than BL [12w]	NA	NA	NA	NA (change from BL[12, 52w])	12w PRO remission + 52w endoscopic remission 12w PRO response + 52w CDAI remission

SERENITY [6] NCT02891226	Sands 2021	CDAI<150 [at 12,52w]	SES-CD score of <4 for ileal-colonic disease or <2 for isolated ileal disease, and no subscore >1 [12, 52w]	SF ≤ 2.5 and AP ≤ 1 and no worse than BL [12, 52w]	Reduction of CDAI ≥100 from BL or CDAI <150 [12, 52w]	50% reduction from BL in SES-CD [12, 52w]	≥30% decrease in AP and/or SF and no worse than BL [12, 52w]	NA	NA	NA (change from BL [12, 52w])	NA(median change from BL [12, 52w])	NA
GALAXI-1 [7] NCT03466411	Danese 2024	CDAI<150 [12,48w]	SES-CD≤ 2[48w]	SF ≤ 3 and AP ≤ 1 and no worse than BL [12, 48w]	Reduction of CDAI ≥100 from BL or CDAI <150 [12, 48w]	decrease in SES-CD ≥50% from BL or SES-CD ≤ 2 [48w]	NA	NA	absence of mucosal ulceration [48w]	remission: IBDQ≥170 response: IBDQ 16-point increase from BL [24,48w]	Clinical response and 50% reduction from BL in CRP or FCP [24, 48w]	NA
GALAXI 2, 3** [8] NCT03466411	Panaccion e 2024	CDAI<150 [12, 48w]	SES-CD ≤4 and at least a 2-point reduction versus BL and no subscore >1[48w]	NA	Reduction of CDAI ≥100 from BL or CDAI <150 [48w]	decrease in SES-CD ≥50% from BL or SES-CD ≤ 2 [12, 48w]	NA	achieving both clinical and endoscopic remission[48w]	NA	NA	NA	12w clinical response + 48w clinical remission; 12w clinical response + 48w endoscopic remission
GRAVITI [9] NCT05197049	Hart 2025	CDAI<150 [12, 24, 48w]	SES-CD ≤4 and at least a 2-point reduction vs BL and no subscore >1 [12, 48w]	AP ≤1 and SF ≤3 and no worsening of AP or SF from BL [12w]	Reduction of CDAI ≥100 from BL [12, 24, 48w]	decrease in SES-CD >50% from BL (at 12w) decrease in SES-CD >50% from BL or SES-CD ≤ 2 [12, 48w]	NA	achieving both clinical and endoscopic remission[48w]	NA	remission: IBDQ≥170 [at 48w]	Elevated CRP (>5 mg/L) and elevated FCP (>250 mg/kg) [48w]	12w clinical remission + 12w endoscopic response
UNITI-1 [10] NCT01369329	Feagon 2016	CDAI<150 [8w]	NA	NA	Reduction of CDAI ≥100 from BL or CDAI <150 [8w]	NA	NA	NA	absence of ulcers [8w]	change of ≥16 points in IBDQ score [8w]	CRP ≤3 mg/L [8w];FCP≤250 μg/g [6w]	NA
UNITI-2 [10] NCT01369342	Feagon 2016	CDAI<150 [8w]	NA	NA	Reduction of CDAI ≥100 from BL or CDAI <150 [8w]	NA	NA	NA	absence of ulcers [8w]	change of ≥16 points in IBDQ score [8w]	CRP ≤3mg/L [8w];FCP≤250 μg/g [6w]	NA
IM-UNITI [10] NCT01369355	Feagon 2016	CDAI<150 [44w]	NA	NA	Reduction of CDAI ≥100 from BL or CDAI <150 [44w]	NA	NA	NA	absence of ulcers [44, 52w]	change of ≥16 points in IBDQ score [22,44w]	FCP≤250 μg/g [44w]	NA
CERTIFI [11] NCT00771667	Sandborn 2012	CDAI<150 [8, 22w]	NA	NA	Reduction of CDAI ≥100 from BL [8, 22w]	NA	NA	NA	absence of mucosal ulcerations [6, 22w]	NA	CRP≥3mg/L [6, 8, 22w]	NA

*Author and year are based on the first author and publication year from the primary publication of each trail.
**pooled GALAXI 2 and 3 data
Abbreviations: BL, baseline, CDAI, Crohn's Disease Activity Index, SD, standard, SES-CD, Simple Endoscopic Score for Crohn's Disease, SF/APS, stool frequency or abdominal pain score, IBDQ, inflammatory bowel disease questionnaire, CDEIS, Crohn's disease endoscopic index of severity, RZB, risankizumab, MRK, mirikizumab, GUS, guselkumab, UST, ustekinumab, PBO, placebo, w, week, NA, not available, CRP, C-reactive protin, FCP, fecal calprotectin.

Supplementary Table S6. Cochrane Risk of Bias for Randomized Trials.

	Random sequence generation (selection bias)	Allocation concealment (selection bias)	Blinding of participants and personnel (performance bias)	Blinding of outcome assessment (detection bias)	Incomplete outcome data (attrition bias)	Selective reporting (reporting bias)	Other bias	Global rating
SEQUENCE	low risk	low risk	high risk	high risk	high risk	low risk	low risk	high risk
ADVANCE	low risk	low risk	low risk	low risk	low risk	low risk	unclear risk	low risk
MOTIVATE	low risk	low risk	low risk	low risk	low risk	low risk	unclear risk	low risk
FORTIFY	low risk	low risk	low risk	low risk	low risk	low risk	unclear risk	low risk
M15-993	low risk	low risk	low risk	low risk	low risk	low risk	low risk	low risk
VIVID-1	low risk	low risk	low risk	low risk	low risk	low risk	low risk	low risk
SERENITY	low risk	low risk	low risk	low risk	low risk	low risk	low risk	low risk
GALAXI-1	low risk	low risk	low risk	low risk	low risk	low risk	low risk	low risk
GALAXI-2	low risk	low risk	low risk	low risk	low risk	low risk	low risk	low risk
GALAXI-3	low risk	low risk	low risk	low risk	low risk	low risk	low risk	low risk
GRAVITI	low risk	low risk	low risk	low risk	low risk	low risk	low risk	low risk
UNITI-1	low risk	low risk	low risk	low risk	low risk	low risk	low risk	low risk
UNITI-2	low risk	low risk	low risk	low risk	low risk	low risk	low risk	low risk
IM-UNITI	low risk	low risk	low risk	low risk	high risk	low risk	low risk	low risk
CERTIFI	low risk	low risk	low risk	low risk	low risk	low risk	low risk	low risk

Global rating was assigned in three levels (high, moderate, and low risk).
A study is judged to have a low risk of bias if it contains zero items with a high risk of bias and three or fewer items with an unclear risk. A study is classified as having a moderate risk of bias under two conditions: if it has zero high-risk items but more than three unclear-risk items; or if it has exactly one high-risk item, irrespective of the

number of unclear-risk items. A study is deemed to have a high risk of bias if it contains more than one item with a high risk of bias, regardless of the number of items with an unclear risk.

Global rating	Items with high risk	Items with unclear risk
low risk	0	=<3
moderate	0	>3
moderate	1	any
high risk	>1	any

Supplementary Table S7. Confidence in network meta-analysis evaluating the confidence of indirect and direct evidence by CINeMA.

Induction

(a) Clinical remission

Comparison	Number of studies	Within-study bias	Reporting bias	Indirectness	Imprecision	Heterogeneity	Incoherence	Confidence rating	Reason(s) for downgrading
Mixed evidence									
GUS-HD:GUS-LD	1	No concerns	Low risk	No concerns	Some concerns	No concerns	No concerns	Moderate	Imprecision
GUS-HD:PBO	1	No concerns	Low risk	No concerns	No concerns	No concerns	No concerns	High	NA
GUS-HD:UST-HD	1	No concerns	Low risk	No concerns	Major concerns	No concerns	No concerns	Low	Imprecision
GUS-LD:PBO	3	No concerns	Low risk	No concerns	No concerns	No concerns	No concerns	High	NA
GUS-LD:UST-HD	1	No concerns	Low risk	No concerns	No concerns	No concerns	No concerns	High	NA
MRK-HD:MRK-LD	1	No concerns	Low risk	No concerns	Major concerns	No concerns	No concerns	Low	Imprecision
MRK-HD:PBO	2	No concerns	Low risk	No concerns	No concerns	No concerns	No concerns	High	NA
MRK-LD:PBO	1	No concerns	Low risk	No concerns	No concerns	No concerns	No concerns	High	NA
PBO:RZB-HD	2	No concerns	Low risk	No concerns	No concerns	No concerns	No concerns	High	NA
PBO:RZB-LD	3	No concerns	Low risk	No concerns	No concerns	No concerns	No concerns	High	NA
PBO:UST-HD	4	No concerns	Low risk	No concerns	No concerns	No concerns	No concerns	High	NA
PBO:UST-LD	3	No concerns	Low risk	No concerns	No concerns	No concerns	No concerns	High	NA
RZB-HD:RZB-LD	2	No concerns	Low risk	No concerns	Some concerns	No concerns	Major concerns	Low	Imprecision, Incoherence
RZB-LD:UST-HD	1	Some concerns	Low risk	No concerns	No concerns	No concerns	Major concerns	Very low	Within-study bias, Incoherence
UST-HD:UST-LD	3	No concerns	Low risk	No concerns	Some concerns	No concerns	No concerns	Moderate	Imprecision
Indirect evidence									
GUS-HD:MRK-HD	0	No concerns	Low risk	No concerns	Major concerns	No concerns	No concerns	Low	Imprecision
GUS-HD:MRK-LD	0	No concerns	Low risk	No concerns	Major concerns	No concerns	No concerns	Low	Imprecision
GUS-HD:RZB-HD	0	No concerns	Low risk	No concerns	Major concerns	No concerns	No concerns	Low	Imprecision
GUS-HD:RZB-LD	0	No concerns	Low risk	No concerns	Some concerns	No concerns	No concerns	Moderate	Imprecision
GUS-HD:UST-LD	0	No concerns	Low risk	No concerns	Some concerns	No concerns	No concerns	Moderate	Imprecision
GUS-LD:MRK-HD	0	No concerns	Low risk	No concerns	Some concerns	No concerns	No concerns	Moderate	Imprecision
GUS-LD:MRK-LD	0	No concerns	Low risk	No concerns	Major concerns	No concerns	No concerns	Low	Imprecision

GUS-LD:RZB-HD	0	No concerns	Low risk	No concerns	Some concerns	No concerns	No concerns	Moderate	Imprecision
GUS-LD:RZB-LD	0	No concerns	Low risk	No concerns	Some concerns	No concerns	No concerns	Moderate	Imprecision
GUS-LD:UST-LD	0	No concerns	Low risk	No concerns	No concerns	No concerns	No concerns	High	NA
MRK-HD:RZB-HD	0	No concerns	Low risk	No concerns	Major concerns	No concerns	No concerns	Low	Imprecision
MRK-HD:RZB-LD	0	No concerns	Low risk	No concerns	Major concerns	No concerns	No concerns	Low	Imprecision
MRK-HD:UST-HD	0	No concerns	Low risk	No concerns	Some concerns	No concerns	No concerns	Moderate	Imprecision
MRK-HD:UST-LD	0	No concerns	Low risk	No concerns	No concerns	Some concerns	No concerns	Moderate	Heterogeneity
MRK-LD:RZB-HD	0	No concerns	Low risk	No concerns	Major concerns	No concerns	No concerns	Low	Imprecision
MRK-LD:RZB-LD	0	No concerns	Low risk	No concerns	Major concerns	No concerns	No concerns	Low	Imprecision
MRK-LD:UST-HD	0	No concerns	Low risk	No concerns	Major concerns	No concerns	No concerns	Low	Imprecision
MRK-LD:UST-LD	0	No concerns	Low risk	No concerns	Some concerns	No concerns	No concerns	Moderate	Imprecision
RZB-HD:UST-HD	0	Some concerns	Low risk	No concerns	Some concerns	No concerns	No concerns	Low	Within-study bias, Imprecision
RZB-HD:UST-LD	0	No concerns	Low risk	No concerns	No concerns	No concerns	No concerns	High	NA
RZB-LD:UST-LD	0	Some concerns	Low risk	No concerns	No concerns	No concerns	No concerns	Moderate	Within-study bias

(b) Clinical response

Comparison	Number of studies	Within-study bias	Reporting bias	Indirectness	Imprecision	Heterogeneity	Incoherence	Confidence rating	Reason(s) for downgrading
Mixed evidence									
GUS-HD:GUS-LD	1	No concerns	Low risk	No concerns	Some concerns	No concerns	No concerns	Moderate	Imprecision
GUS-HD:PBO	1	No concerns	Low risk	No concerns	No concerns	No concerns	No concerns	High	NA
GUS-HD:UST-HD	1	No concerns	Low risk	No concerns	Some concerns	No concerns	No concerns	Moderate	Imprecision
GUS-LD:PBO	2	No concerns	Low risk	No concerns	No concerns	No concerns	No concerns	High	NA
GUS-LD:UST-HD	1	No concerns	Low risk	No concerns	Some concerns	No concerns	No concerns	Moderate	Imprecision
MRK-HD:MRK-LD	1	No concerns	Low risk	No concerns	Some concerns	No concerns	Some concerns	Moderate	Imprecision, Incoherence
MRK-HD:PBO	1	No concerns	Low risk	No concerns	No concerns	Some concerns	Some concerns	Moderate	Heterogeneity, Incoherence
MRK-LD:PBO	1	No concerns	Low risk	No concerns	No concerns	No concerns	Some concerns	Moderate	Incoherence
PBO:RZB-HD	2	No concerns	Low risk	No concerns	No concerns	No concerns	No concerns	High	NA

PBO:RZB-LD	3	No concerns	Low risk	No concerns	No concerns	No concerns	No concerns	High	NA
PBO:UST-HD	4	No concerns	Low risk	No concerns	No concerns	No concerns	No concerns	High	NA
PBO:UST-LD	3	No concerns	Low risk	No concerns	No concerns	No concerns	No concerns	High	NA
RZB-HD:RZB-LD	2	No concerns	Low risk	No concerns	Some concerns	No concerns	Major concerns	Low	Imprecision, Incoherence
RZB-LD:UST-HD	1	Some concerns	Low risk	No concerns	Some concerns	No concerns	Major concerns	Very low	Within-study bias, Imprecision, Incoherence
UST-HD:UST-LD	3	No concerns	Low risk	No concerns	No concerns	Some concerns	No concerns	Moderate	Heterogeneity
Indirect evidence									
GUS-HD:MRK-HD	0	No concerns	Low risk	No concerns	Major concerns	No concerns	Some concerns	Low	Imprecision, Incoherence
GUS-HD:MRK-LD	0	No concerns	Low risk	No concerns	Major concerns	No concerns	Some concerns	Low	Imprecision, Incoherence
GUS-HD:RZB-HD	0	No concerns	Low risk	No concerns	Some concerns	No concerns	Some concerns	Moderate	Imprecision, Incoherence
GUS-HD:RZB-LD	0	No concerns	Low risk	No concerns	Some concerns	No concerns	Some concerns	Moderate	Imprecision, Incoherence
GUS-HD:UST-LD	0	No concerns	Low risk	No concerns	Some concerns	No concerns	Some concerns	Moderate	Imprecision, Incoherence
GUS-LD:MRK-HD	0	No concerns	Low risk	No concerns	Major concerns	No concerns	Some concerns	Low	Imprecision, Incoherence
GUS-LD:MRK-LD	0	No concerns	Low risk	No concerns	Major concerns	No concerns	Some concerns	Low	Imprecision, Incoherence
GUS-LD:RZB-HD	0	No concerns	Low risk	No concerns	Some concerns	No concerns	Some concerns	Moderate	Imprecision, Incoherence
GUS-LD:RZB-LD	0	No concerns	Low risk	No concerns	Some concerns	No concerns	Some concerns	Moderate	Imprecision, Incoherence
GUS-LD:UST-LD	0	No concerns	Low risk	No concerns	No concerns	No concerns	Some concerns	Moderate	Incoherence
MRK-HD:RZB-HD	0	No concerns	Low risk	No concerns	Major concerns	No concerns	Some concerns	Low	Imprecision, Incoherence
MRK-HD:RZB-LD	0	No concerns	Low risk	No concerns	Major concerns	No concerns	Some concerns	Low	Imprecision, Incoherence
MRK-HD:UST-HD	0	No concerns	Low risk	No concerns	Major concerns	No concerns	Some concerns	Low	Imprecision, Incoherence
MRK-HD:UST-LD	0	No concerns	Low risk	No concerns	Major concerns	No concerns	Some concerns	Low	Imprecision, Incoherence
MRK-LD:RZB-HD	0	No concerns	Low risk	No concerns	Major concerns	No concerns	Some concerns	Low	Imprecision, Incoherence
MRK-LD:RZB-LD	0	No concerns	Low risk	No concerns	Major concerns	No concerns	Some concerns	Low	Imprecision, Incoherence
MRK-LD:UST-HD	0	No concerns	Low risk	No concerns	Major concerns	No concerns	Some concerns	Low	Imprecision, Incoherence
MRK-LD:UST-LD	0	No concerns	Low risk	No concerns	Some concerns	No concerns	Some concerns	Moderate	Imprecision, Incoherence
RZB-HD:UST-HD	0	No concerns	Low risk	No concerns	Some concerns	No concerns	Some concerns	Moderate	Imprecision, Incoherence
RZB-HD:UST-LD	0	No concerns	Low risk	No concerns	No concerns	No concerns	Some concerns	Moderate	Incoherence
RZB-LD:UST-LD	0	No concerns	Low risk	No concerns	No concerns	No concerns	Some concerns	Moderate	Incoherence

(c) endoscopic remission

Comparison	Number of studies	Within-study bias	Reporting bias	Indirectness	Imprecision	Heterogeneity	Incoherence	Confidence rating	Reason(s) for downgrading
Mixed evidence									
GUS-HD:GUS-LD	1	No concerns	Low risk	No concerns	Major concerns	No concerns	No concerns	Low	Imprecision
GUS-HD:PBO	1	No concerns	Low risk	No concerns	No concerns	Major concerns	No concerns	Low	Heterogeneity
GUS-HD:UST-HD	1	No concerns	Low risk	No concerns	Major concerns	No concerns	No concerns	Low	Imprecision
GUS-LD:PBO	2	No concerns	Low risk	No concerns	No concerns	No concerns	No concerns	High	NA
GUS-LD:UST-HD	1	No concerns	Low risk	No concerns	Major concerns	No concerns	No concerns	Low	Imprecision
MRK-HD:MRK-LD	1	No concerns	Low risk	No concerns	Major concerns	No concerns	No concerns	Low	Imprecision
MRK-HD:PBO	2	No concerns	Low risk	No concerns	No concerns	No concerns	No concerns	High	NA
MRK-LD:PBO	1	No concerns	Low risk	No concerns	Major concerns	No concerns	No concerns	Low	Imprecision
PBO:RZB-HD	2	No concerns	Low risk	No concerns	No concerns	No concerns	No concerns	High	NA
PBO:RZB-LD	2	No concerns	Low risk	No concerns	No concerns	No concerns	No concerns	High	NA
PBO:UST-HD	1	Some concerns	Low risk	No concerns	No concerns	No concerns	No concerns	Moderate	Within-study bias
RZB-HD:RZB-LD	2	No concerns	Low risk	No concerns	Some concerns	No concerns	No concerns	Moderate	Imprecision
RZB-LD:UST-HD	1	Major concerns	Low risk	No concerns	No concerns	No concerns	No concerns	Low	Within-study bias
Indirect evidence									
GUS-HD:MRK-HD	0	No concerns	Low risk	No concerns	Major concerns	No concerns	No concerns	Low	Imprecision
GUS-HD:MRK-LD	0	No concerns	Low risk	No concerns	Major concerns	No concerns	No concerns	Low	Imprecision
GUS-HD:RZB-HD	0	No concerns	Low risk	No concerns	Major concerns	No concerns	No concerns	Low	Imprecision
GUS-HD:RZB-LD	0	No concerns	Low risk	No concerns	Major concerns	No concerns	No concerns	Low	Imprecision
GUS-LD:MRK-HD	0	No concerns	Low risk	No concerns	Major concerns	No concerns	No concerns	Low	Imprecision
GUS-LD:MRK-LD	0	No concerns	Low risk	No concerns	Major concerns	No concerns	No concerns	Low	Imprecision
GUS-LD:RZB-HD	0	No concerns	Low risk	No concerns	Some concerns	No concerns	No concerns	Moderate	Imprecision
GUS-LD:RZB-LD	0	No concerns	Low risk	No concerns	Some concerns	No concerns	No concerns	Moderate	Imprecision
MRK-HD:RZB-HD	0	No concerns	Low risk	No concerns	Major concerns	No concerns	No concerns	Low	Imprecision
MRK-HD:RZB-LD	0	No concerns	Low risk	No concerns	Major concerns	No concerns	No concerns	Low	Imprecision

MRK-HD:UST-HD	0	No concerns	Low risk	No concerns	Major concerns	No concerns	No concerns	Low	Imprecision
MRK-LD:RZB-HD	0	No concerns	Low risk	No concerns	Major concerns	No concerns	No concerns	Low	Imprecision
MRK-LD:RZB-LD	0	No concerns	Low risk	No concerns	Major concerns	No concerns	No concerns	Low	Imprecision
MRK-LD:UST-HD	0	No concerns	Low risk	No concerns	Major concerns	No concerns	No concerns	Low	Imprecision
RZB-HD:UST-HD	0	Some concerns	Low risk	No concerns	No concerns	Some concerns	No concerns	Low	Within-study bias, Heterogeneity

(d) endoscopic response

Comparison	Number of studies	Within-study bias	Reporting bias	Indirectness	Imprecision	Heterogeneity	Incoherence	Confidence rating	Reason(s) for downgrading
Mixed evidence									
GUS-HD:GUS-LD	1	No concerns	Low risk	No concerns	Major concerns	No concerns	No concerns	Low	Imprecision
GUS-HD:PBO	1	No concerns	Low risk	No concerns	No concerns	Some concerns	No concerns	Moderate	Heterogeneity
GUS-HD:UST-HD	1	No concerns	Low risk	No concerns	Major concerns	No concerns	No concerns	Low	Imprecision
GUS-LD:PBO	3	No concerns	Low risk	No concerns	No concerns	No concerns	No concerns	High	NA
GUS-LD:UST-HD	1	No concerns	Low risk	No concerns	Some concerns	No concerns	No concerns	Moderate	Imprecision
MRK-HD:MRK-LD	1	No concerns	Low risk	No concerns	Major concerns	No concerns	No concerns	Low	Imprecision
MRK-HD:PBO	2	No concerns	Low risk	No concerns	No concerns	No concerns	No concerns	High	NA
MRK-LD:PBO	1	No concerns	Low risk	No concerns	No concerns	Some concerns	No concerns	Moderate	Heterogeneity
PBO:RZB-HD	2	No concerns	Low risk	No concerns	No concerns	No concerns	No concerns	High	NA
PBO:RZB-LD	2	No concerns	Low risk	No concerns	No concerns	No concerns	No concerns	High	NA
PBO:UST-HD	1	No concerns	Low risk	No concerns	No concerns	Some concerns	No concerns	Moderate	Heterogeneity
RZB-HD:RZB-LD	2	No concerns	Low risk	No concerns	Some concerns	No concerns	No concerns	Moderate	Imprecision
RZB-LD:UST-HD	1	Some concerns	Low risk	No concerns	No concerns	No concerns	No concerns	Moderate	Within-study bias
Indirect evidence									
GUS-HD:MRK-HD	0	No concerns	Low risk	No concerns	Major concerns	No concerns	No concerns	Low	Imprecision
GUS-HD:MRK-LD	0	No concerns	Low risk	No concerns	Major concerns	No concerns	No concerns	Low	Imprecision
GUS-HD:RZB-HD	0	No concerns	Low risk	No concerns	Major concerns	No concerns	No concerns	Low	Imprecision
GUS-HD:RZB-LD	0	No concerns	Low risk	No concerns	Some concerns	No concerns	No concerns	Moderate	Imprecision

GUS-LD:MRK-HD	0	No concerns	Low risk	No concerns	Major concerns	No concerns	No concerns	Low	Imprecision
GUS-LD:MRK-LD	0	No concerns	Low risk	No concerns	Major concerns	No concerns	No concerns	Low	Imprecision
GUS-LD:RZB-HD	0	No concerns	Low risk	No concerns	Some concerns	No concerns	No concerns	Moderate	Imprecision
GUS-LD:RZB-LD	0	No concerns	Low risk	No concerns	Some concerns	No concerns	No concerns	Moderate	Imprecision
MRK-HD:RZB-HD	0	No concerns	Low risk	No concerns	Major concerns	No concerns	No concerns	Low	Imprecision
MRK-HD:RZB-LD	0	No concerns	Low risk	No concerns	Major concerns	No concerns	No concerns	Low	Imprecision
MRK-HD:UST-HD	0	No concerns	Low risk	No concerns	Some concerns	No concerns	No concerns	Moderate	Imprecision
MRK-LD:RZB-HD	0	No concerns	Low risk	No concerns	Major concerns	No concerns	No concerns	Low	Imprecision
MRK-LD:RZB-LD	0	No concerns	Low risk	No concerns	Major concerns	No concerns	No concerns	Low	Imprecision
MRK-LD:UST-HD	0	No concerns	Low risk	No concerns	Major concerns	No concerns	No concerns	Low	Imprecision
RZB-HD:UST-HD	0	Some concerns	Low risk	No concerns	No concerns	Some concerns	No concerns	Low	Within-study bias, Heterogeneity

Maintenance

(d) clinical remission

Comparison	Number of studies	Within-study bias	Reporting bias	Indirectness	Imprecision	Heterogeneity	Incoherence	Confidence rating	Reason(s) for downgrading
Mixed evidence									
GUS 100mg:GUS 200mg	3	No concerns	Low risk	No concerns	Major concerns	No concerns	Major concerns	Low	Imprecision, Incoherence
GUS 100mg:PBO	1	No concerns	Low risk	No concerns	No concerns	No concerns	No concerns	High	NA
GUS 100mg:UST	2	No concerns	Low risk	No concerns	Some concerns	No concerns	No concerns	Moderate	Imprecision
GUS 200mg:PBO	1	No concerns	Low risk	No concerns	No concerns	No concerns	No concerns	High	NA
GUS 200mg:UST	2	No concerns	Low risk	No concerns	Some concerns	No concerns	Major concerns	Low	Imprecision, Incoherence
MRK:PBO	1	No concerns	Low risk	No concerns	No concerns	Some concerns	No concerns	Moderate	Heterogeneity
MRK:UST	1	No concerns	Low risk	No concerns	Some concerns	No concerns	No concerns	Moderate	Imprecision
PBO:RZB	1	No concerns	Low risk	No concerns	No concerns	Some concerns	Major concerns	Low	Heterogeneity, Incoherence
PBO:UST	3	No concerns	Low risk	No concerns	No concerns	Some concerns	No concerns	Moderate	Heterogeneity
RZB:UST	1	Some concerns	Low risk	No concerns	Major concerns	No concerns	No concerns	Very low	Within-study bias, Imprecision
Indirect evidence									
GUS 100mg:MRK	0	No concerns	Low risk	No concerns	Major concerns	No concerns	Major concerns	Low	Imprecision, Incoherence
GUS 100mg:RZB	0	Some concerns	Low risk	No concerns	Major concerns	No concerns	Major concerns	Very low	Within-study bias, Imprecision, Incoherence
GUS 200mg:MRK	0	No concerns	Low risk	No concerns	Major concerns	No concerns	Major concerns	Low	Imprecision, Incoherence
GUS 200mg:RZB	0	Some concerns	Low risk	No concerns	Major concerns	No concerns	Major concerns	Very low	Within-study bias, Imprecision, Incoherence
MRK:RZB	0	Some concerns	Low risk	No concerns	Major concerns	No concerns	Major concerns	Very low	Within-study bias, Imprecision, Incoherence

(e) clinical response

Comparison	Number of studies	Within-study bias	Reporting bias	Indirectness	Imprecision	Heterogeneity	Incoherence	Confidence rating	Reason(s) for downgrading
Mixed evidence									
GUS 100mg:GUS 200mg	1	No concerns	Low risk	No concerns	Major concerns	No concerns	Some concerns	Low	Imprecision, Incoherence
GUS 100mg:PBO	1	No concerns	Low risk	No concerns	No concerns	Major concerns	Some concerns	Low	Heterogeneity, Incoherence

GUS 200mg:PBO	1	No concerns	Low risk	No concerns	No concerns	Major concerns	Some concerns	Low	Heterogeneity, Incoherence
MRK:UST	1	No concerns	Low risk	No concerns	Major concerns	No concerns	Some concerns	Low	Imprecision, Incoherence
PBO:RZB	1	No concerns	Low risk	No concerns	No concerns	Major concerns	No concerns	Low	Heterogeneity
PBO:UST	2	No concerns	Low risk	No concerns	Major concerns	No concerns	No concerns	Low	Imprecision
RZB:UST	1	Some concerns	Low risk	No concerns	Some concerns	No concerns	No concerns	Low	Within-study bias, Imprecision
Indirect evidence									
GUS 100mg:MRK	0	No concerns	Low risk	No concerns	No concerns	Major concerns	Some concerns	Low	Heterogeneity, Incoherence
GUS 100mg:RZB	0	No concerns	Low risk	No concerns	No concerns	Major concerns	Some concerns	Low	Heterogeneity, Incoherence
GUS 100mg:UST	0	No concerns	Low risk	No concerns	No concerns	Major concerns	Some concerns	Low	Heterogeneity, Incoherence
GUS 200mg:MRK	0	No concerns	Low risk	No concerns	No concerns	Major concerns	Some concerns	Low	Heterogeneity, Incoherence
GUS 200mg:RZB	0	No concerns	Low risk	No concerns	No concerns	Major concerns	Some concerns	Low	Heterogeneity, Incoherence
GUS 200mg:UST	0	No concerns	Low risk	No concerns	No concerns	Major concerns	Some concerns	Low	Heterogeneity, Incoherence
MRK:PBO	0	No concerns	Low risk	No concerns	Some concerns	No concerns	Some concerns	Low	Imprecision, Incoherence
MRK:RZB	0	Some concerns	Low risk	No concerns	Major concerns	No concerns	Some concerns	Low	Within-study bias, Imprecision, Incoherence

(f) endoscopic remission

Comparison	Number of studies	Within-study bias	Reporting bias	Indirectness	Imprecision	Heterogeneity	Incoherence	Confidence rating	Reason(s) for downgrading
Mixed evidence									
GUS 100mg:GUS 200mg	3	No concerns	Low risk	No concerns	Some concerns	No concerns	No concerns	Moderate	Imprecision
GUS 100mg:PBO	1	No concerns	Low risk	No concerns	No concerns	Some concerns	No concerns	Moderate	Heterogeneity
GUS 100mg:UST	2	No concerns	Low risk	No concerns	No concerns	Major concerns	No concerns	Low	Heterogeneity
GUS 200mg:PBO	1	No concerns	Low risk	No concerns	No concerns	No concerns	No concerns	High	NA
GUS 200mg:UST	2	No concerns	Low risk	No concerns	No concerns	Major concerns	No concerns	Low	Heterogeneity
MRK:UST	1	No concerns	Low risk	No concerns	Major concerns	No concerns	No concerns	Low	Imprecision
PBO:RZB	1	No concerns	Low risk	No concerns	No concerns	No concerns	No concerns	High	NA
RZB:UST	1	Some concerns	Low risk	No concerns	Some concerns	No concerns	No concerns	Low	Within-study bias, Imprecision
Indirect evidence									

GUS 100mg:MRK	0	No concerns	Low risk	No concerns	Major concerns	No concerns	No concerns	Low	Imprecision
GUS 100mg:RZB	0	Some concerns	Low risk	No concerns	Major concerns	No concerns	No concerns	Very low	Within-study bias, Imprecision
GUS 200mg:MRK	0	No concerns	Low risk	No concerns	Some concerns	No concerns	No concerns	Moderate	Imprecision
GUS 200mg:RZB	0	Some concerns	Low risk	No concerns	Major concerns	No concerns	No concerns	Very low	Within-study bias, Imprecision
MRK:PBO	0	No concerns	Low risk	No concerns	Some concerns	No concerns	No concerns	Moderate	Imprecision
MRK:RZB	0	Some concerns	Low risk	No concerns	Major concerns	No concerns	No concerns	Very low	Within-study bias, Imprecision
PBO:UST	0	No concerns	Low risk	No concerns	No concerns	Major concerns	No concerns	Low	Heterogeneity

(g) endoscopic response

Comparison	Number of studies	Within-study bias	Reporting bias	Indirectness	Imprecision	Heterogeneity	Incoherence	Confidence rating	Reason(s) for downgrading
Mixed evidence									
GUS 100mg:GUS 200mg	3	No concerns	Low risk	No concerns	Major concerns	No concerns	Major concerns	Low	Imprecision, Incoherence
GUS 100mg:PBO	1	No concerns	Low risk	No concerns	No concerns	No concerns	No concerns	High	NA
GUS 100mg:UST	2	No concerns	Low risk	No concerns	Some concerns	No concerns	No concerns	Moderate	Imprecision
GUS 200mg:PBO	1	No concerns	Low risk	No concerns	No concerns	No concerns	No concerns	High	NA
GUS 200mg:UST	2	No concerns	Low risk	No concerns	Some concerns	No concerns	No concerns	Moderate	Imprecision
MRK:PBO	1	No concerns	Low risk	No concerns	No concerns	Some concerns	No concerns	Moderate	Heterogeneity
MRK:UST	1	No concerns	Low risk	No concerns	Major concerns	No concerns	No concerns	Low	Imprecision
PBO:RZB	1	No concerns	Low risk	No concerns	No concerns	No concerns	Major concerns	Low	Incoherence
PBO:UST	1	No concerns	Low risk	No concerns	No concerns	Some concerns	No concerns	Moderate	Heterogeneity
RZB:UST	1	Some concerns	Low risk	No concerns	Major concerns	No concerns	No concerns	Very low	Within-study bias, Imprecision
Indirect evidence									
GUS 100mg:MRK	0	No concerns	Low risk	No concerns	Major concerns	No concerns	Major concerns	Low	Imprecision, Incoherence
GUS 100mg:RZB	0	Some concerns	Low risk	No concerns	Major concerns	No concerns	Major concerns	Very low	Within-study bias, Imprecision, Incoherence
GUS 200mg:MRK	0	No concerns	Low risk	No concerns	Major concerns	No concerns	Major concerns	Low	Imprecision, Incoherence
GUS 200mg:RZB	0	Some concerns	Low risk	No concerns	Major concerns	No concerns	Major concerns	Very low	Within-study bias, Imprecision, Incoherence

MRK:RZB	0	Some concerns	Low risk	No concerns	Major concerns	No concerns	Major concerns	Very low	Within-study bias, Imprecision, Incoherence

CINeMA assigns judgments at 3 levels (no concerns, some concerns, or major concerns) to each domain. Judgments across domains can be summarized to obtain 4 levels of confidence for each relative treatment effect, corresponding to the usual GRADE assessments of very low, low, moderate, or high. The quality of evidence was downgraded by two levels if there were "major concerns" in one area and downgraded by one level if there were "some concerns" in two areas.

Definition of reasons for downgrading: 1) *Within-study bias* refers to methodological shortcomings in the design or conduct of the individual studies included in a meta-analysis, which can lead to a systematic deviation of the estimated relative treatment effect from the true value; 2) *Reporting bias* arises from the selective publication or dissemination of research findings, typically where statistically significant ('positive') results are more likely to be published and available for inclusion than non-significant or unfavorable ones, thus distorting the overall evidence base; 3) *Indirectness* addresses the relevance and applicability of the included studies to the review's specific research question, concerning mismatches in the populations, interventions, comparators, outcomes, or settings (PICOS) between the available evidence and the context the review aims to inform; 4) Imprecision describes the uncertainty in the estimated treatment effect, which is reflected in the width of its confidence interval; it is assessed by evaluating whether this interval extends into ranges that represent clinically important benefit or harm relative to a pre-defined range of equivalence; 5) *Heterogeneity* signifies the genuine variability in treatment effects across the included studies beyond what would be expected by chance alone, which reduces the confidence that a single summary estimate is representative of the true effect in different settings; 6) *Incoherence* (also known as inconsistency) is the statistical manifestation of intransitivity in a network meta-analysis, occurring when the direct evidence for a treatment comparison meaningfully disagrees with the indirect evidence obtained through a common comparator.

RZB, Risankizumab, MRK, Mirikizumab, GUS, Guselkumab, UST, Ustekinumab, PBO, placebo, LD, low dose, HD, high dose.

Supplementary Table S8. Summary of safety outcomes from randomized controlled trials included in the network meta-analysis.
Induction

Trails	Author Year*	Phase	Treatment arms	N, n	Follow-up, w	Any AE, n (%)	SAE, n (%)	Serious infection, n (%)	Discontiuation due to AE, n (%)	Malignancy, n (%)	Death, n (%)
ADVANCE [3]	D'Haens 2022	III	Risankizumab 600mg<br>Risankizumab 1200mg<br>Placebo	336<br>339<br>175	12	210 (56)<br>191 (51)<br>105 (56)	27 (7)<br>14 (4)<br>28 (15)	3 (1)<br>2 (1)<br>7 (4)	9 (2)<br>7 (2)<br>14 (8)	0<br>0<br>0	0<br>0<br>2 (1)
MOTIVATE [3]	D'Haens 2022	III	Risankizumab 600mg<br>Risankizumab 1200mg<br>Placebo	191<br>191<br>187	12	98 (48)<br>121 (59)<br>137 (66)	10 (5)<br>9 (4)<br>26 (13)	1 (<1)<br>2 (1)<br>5 (2)	2 (1)<br>5 (2)<br>17 (8)	0<br>1 (<1)<br>0	0<br>1 (<1)<br>0
M15-993 [4]	Feagan 2018	II	Risankizumab 200mg<br>Risankizumab 600mg<br>Placebo	41<br>41<br>39	12	32 (78)<br>31 (76)<br>32 (82)	9 (22)<br>3 (7)<br>12 (31)	NA	5 (12)<br>1 (2)<br>6 (15)	NA	NA
SERENITY [6]	Sands 2021	II	Mirikizumab 200mg<br>Mirikizumab 600mg<br>Mirikizumab 1000mg<br>Placebo	31<br>32<br>64<br>64	12	18 (58.1)<br>21 (65.6)<br>42 (65.6)<br>45 (70.3)	0<br>3 (9.4)<br>2 (3.1)<br>7 (10.9)	NA	1 (3.2)<br>3 (9.4)<br>0<br>3 (4.7)	NA	NA
UNITI-1 [10]	Feagon 2016	III	Ustekinumab 130mg<br>Ustekinumab 6mg/kg<br>Placebo	245<br>249<br>247	8	159 (64.6)<br>164 (65.9)<br>159 (64.9)	12 (4.9)<br>18 (7.2)<br>15 (6.1)	3 (1.2)<br>7 (2.8)<br>3 (1.2)	NA	NA	NA
UNITI-2 [10]	Feagon 2016	III	Ustekinumab 130mg<br>Ustekinumab 6mg/kg<br>Placebo	209<br>209<br>210	8	106 (50.0)<br>115 (55.6)<br>113 (54.3)	10 (4.7)<br>6 (2.9)<br>12 (5.8)	3 (1.4)<br>1 (0.5)<br>3 (1.4)	NA	NA	NA
CERTIFI [11]	Sandborn 2012	II	Ustekinumab 1mg/kg<br>Ustekinumab 3mg/kg<br>Ustekinumab 6mg/kg<br>Placebo	131<br>132<br>131<br>132	8	89 (68.5)<br>88 (66.2)<br>80 (61.1)<br>94 (71.2)	6 (4.6)<br>8 (6.0)<br>9 (6.9)<br>11 (8.3)	1 (0.8)<br>0<br>5 (3.8)<br>1 (0.8)	NA	NA	NA

*Author and year are based on the first author and publication year from the primary publication of each trail.**pooled GALAXI 2 and 3 data
Abbreviations: AE, adverse event, RZB, risankizumab, MRK, mirikizumab, GUS, guselkumab, UST, ustekinumab, PBO, placebo, w, week, N, number of sample, n, number, NA, not available, SAE, serious adverse event (Serious adverse events were defined as any of the following events: death, a life-threatening event, hospitalization, prolongation of hospitalization, a congenital anomaly, persistent or substantial disability or incapacity, an event that led to medical or surgical intervention to prevent a serious outcome).

maintenance

Trails	Author Year*	Phase	Treatment arms	N, n	Follow-up, w	Any AE, n (%)	SAE, n (%)	Serious infection, n (%)	Discontiuation due to AE, n (%)	Malignancy, n (%)	Death, n (%)
SEQUENCE [1]	Peyrin-Biroulet 2024	III	Risankizumab 360mg<br>Ustekinumab 90mg	255<br>265	48	223 (85.1)<br>219 (82.6)	27 (10.3)<br>36 (14.0)	8 (3.1)<br>11 (4.2)	10 (3.8)<br>13 (4.9)	1 (0.4)<br>1 (0.4)	0<br>0
FORTIFY [2]	Ferrante 2022	III	Risankizumab 180mg<br>Risankizumab 360mg<br>Placebo	157<br>141<br>164	52	128 (72)<br>129 (72)<br>135 (73)	22 (12)<br>24 (13)<br>23 (13)	5 (3)<br>8 (4)<br>7 (4)	3 (2)<br>6 (3)<br>6 (3)	0<br>1 (1)<br>1 (1)	0<br>0<br>0
VIVID-1 [5]	Jairath 2024	III	Mirikizumab 300mg<br>Ustekinumab 90mg<br>Placebo	579<br>287<br>199	52	495 (78.6)<br>239 (77.3)<br>154 (73.0)	65 (10.3)<br>33 (10.7)<br>36 (17.1)	14 (2.2)<br>9 (2.9)<br>6 (2.8)	32 (5.1)<br>8 (2.6)<br>20 (9.5)	2 (0.3)<br>0<br>1 (0.5)	0<br>1 (0.3)<br>1 (0.5)
GALAXI 1 [7]	Danese 2024	II	Guselkumab 200-100mg<br>Guselkumab 600-200mg<br>Guselkumab 1200-200mg<br>Ustekinumab 90mg<br>Placebo	73<br>73<br>73<br>71<br>70	48	52 (71)<br>59 (81)<br>51 (70)<br>60 (85)<br>46 (66)	6 (8)<br>5 (7)<br>5 (7)<br>9 (13)<br>6 (9)	2 (3)<br>2 (3)<br>1 (1)<br>1 (1)<br>1 (1)	5 (7)<br>2 (3)<br>6 (8)<br>6 (8)<br>4 (6)	NA	0<br>0<br>0<br>0<br>0
GALAXI 2, 3**[8]	Panaccione 2024	III	Guselkumab 100mg<br>Guselkumab 200mg<br>Ustekinumab 90mg<br>Placebo	296<br>299<br>300<br>153	48	225 (76)<br>233 (78)<br>236 (79)<br>82 (54)	32 (11)<br>21 (7)<br>35 (12)<br>16 (10)	1 (<1)<br>3 (1)<br>12 (4)<br>2 (1)	21 (7)<br>19 (6)<br>22 (7)<br>13 (8)	0<br>1 (<1)<br>0<br>0	0<br>0<br>0<br>0
GRAVITI [9]	Hart 2025	III	Guselkumab 100mg<br>Guselkumab 200mg<br>Placebo	115<br>115<br>117	48	95 (82.6)<br>92 (80.0)<br>77 (65.8)	15 (13.0)<br>9 (7.8)<br>16 (13.7)	2 (1.7)<br>1 (0.9)<br>0	4 (3.5)<br>3 (2.6)<br>10 (8.5)	1 (0.9)<br>0<br>0	1 (0.9)<br>0<br>0
IM-UNITI [10]	Feagon 2016	III	Ustekinumab 90mg q8w<br>Ustekinumab 90mg q12w<br>Placebo	132<br>132<br>133	44	106 (80.3)<br>107 (81.7)<br>111 (83.5)	16 (12.1)<br>13(9.9)<br>20 (15.0)	7 (5.3)<br>3 (2.3)<br>3 (2.3)	NA	NA	NA
CERTIFI [11]	Sandborn 2012	II	Ustekinumab 90mg<br>Placebo	181<br>183	36	140 (77.3)<br>151 (82.5)	31 (17.1)<br>33 (18.0)	4 (2.2)<br>7 (3.8)	NA	1 (0.6)<br>0	NA

*Author and year are based on the first author and publication year from the primary publication of each trail.**pooled GALAXI 2 and 3 data
Abbreviations: AE, adverse event, RZB, risankizumab, MRK, mirikizumab, GUS, guselkumab, UST, ustekinumab, PBO, placebo, w, week, N, number of sample, n, number, NA, not available, SAE, serious adverse event (Serious adverse events were defined as any of the following events: death, a life-threatening event, hospitalization, prolongation of hospitalization, a congenital anomaly, persistent or substantial disability or incapacity, an event that led to medical or surgical intervention to prevent a serious outcome)

Safety outcomes

Overall, IL-23p19 inhibitors demonstrated favorable and generally comparable safety profiles to both ustekinumab and placebo across the included induction and maintenance trials. Serious adverse events (SAEs) were consistently low across all treatment arms. In the risankizumab induction trials (ADVANCE, MOTIVATE, M15-993), SAE rates ranged from 4% to 7% across doses, compared with 13% to 31% for placebo, while maintenance data from FORTIFY showed SAE rates of 12% to 13% for risankizumab versus 13% for placebo. In the pivotal SEQUENCE trial, SAEs occurred in 10.3% of risankizumab-treated patients versus 14.0% of ustekinumab-treated patients, with the investigators noting that the incidence of adverse events appeared similar between groups[1]. A recent meta-analysis of five head-to-head randomized controlled trials confirmed these observations, finding no significant difference between IL-23p19 inhibitors and ustekinumab in SAEs (risk ratio 0.79 [95% CI 0.61 – 1.02]), serious infections (RR 0.56 [0.25 – 1.24]), or discontinuation due to adverse events (RR 1.01 [0.66 – 1.53])[12].

Serious infections were infrequent across all treatment groups, and no concerning imbalance was observed. Malignancy and death were rare events throughout the trial program. In the guselkumab phase 3 program (GALAXI-2 and GALAXI-3), SAEs occurred in approximately 7% of guselkumab-treated patients versus 11% to 12% with ustekinumab and 12% to 15% with placebo, with no reported deaths in either trial[7]. Similarly, in the VIVID-1 trial, mirikizumab and ustekinumab displayed comparable safety profiles through 52 weeks [5]. Long-term safety data from an integrated analysis of over 4,300 patients and approximately 10,800 patient-years of guselkumab exposure in psoriatic disease reported SAEs at 5.4 per 100 patient-years, serious infections at 1.0 per 100 patient-years, and malignancy at 0.6 per 100 patient-years, rates that were comparable to those observed with placebo [13]. These findings, together with the absence of new safety signals in the GALAXI-1 maintenance cohort [7], support the conclusion that IL-23p19 inhibitors exhibit a reassuring safety profile that is comparable to ustekinumab and placebo across both induction and maintenance phases of Crohn's disease treatment.

Supplementary Figure S1. Network heat plot for clinical remission during induction.

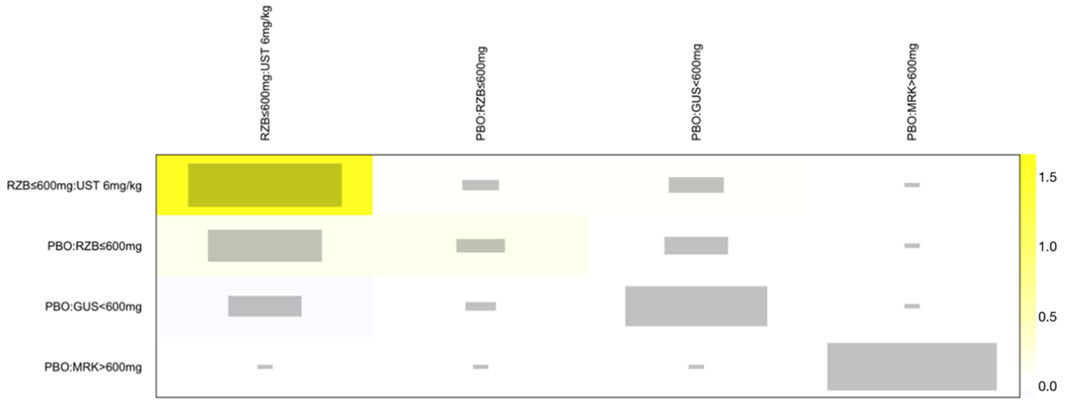

Network heat plot showed no red hotspots of inconsistency, suggesting good agreement between direct and indirect evidence within the network, and supporting the robustness of pooled RR and SUCRA estimates.

Supplementary Figure S2. Forest plot of sensitivity analysis for inducing the clinical remission among all participants, excluding Risankizumab 200mg iv q4w from RZB≤600mg.

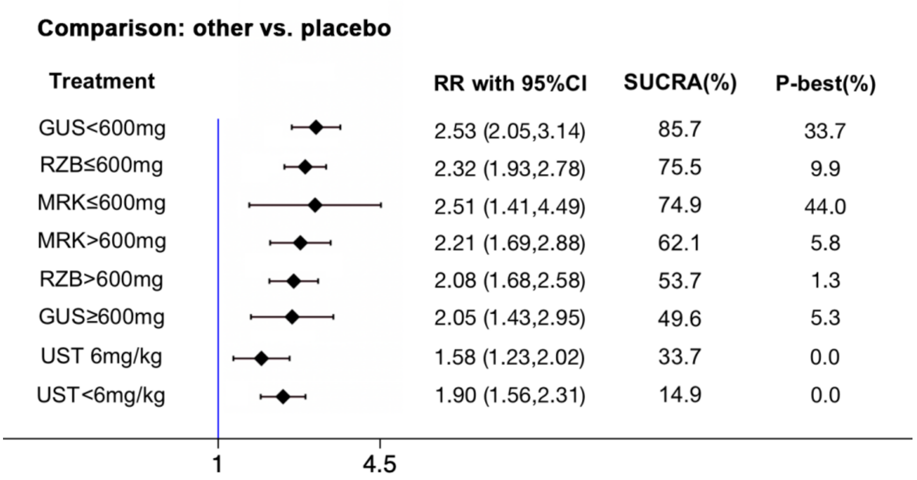

Note: SUCRA provides a cumulative distribution of a treatment's rankings, which quantifies the probability of it being among the most effective interventions. P-best is the probability of each intervention being ranked as best in the network. CD, Crohn's disease; RR, relative risk. PBO, placebo, RZB, Risankizumab, MRK, Mirikizumab, GUS, Guselkumab, UST, Ustekinumab.

Supplementary Figure S3. Forest plot of sensitivity analysis for inducing the clinical response among all participants, excluding Risankizumab 200mg iv q4w from RZB≤600mg.

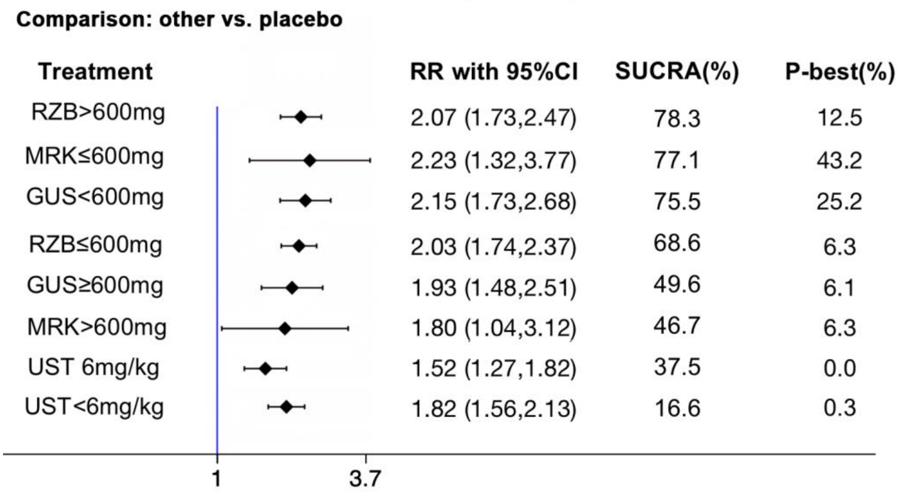

Note: SUCRA provides a cumulative distribution of a treatment's rankings, which quantifies the probability of it being among the most effective interventions. P-best is the probability of each intervention being ranked as best in the network. CD, Crohn's disease; RR, relative risk. PBO, placebo, RZB, Risankizumab, MRK, Mirikizumab, GUS, Guselkumab, UST, Ustekinumab.

Supplementary Figure S4. Forest plot of Sensitivity analysis for inducing the clinical remission among all participants, excluding ADVANCE.

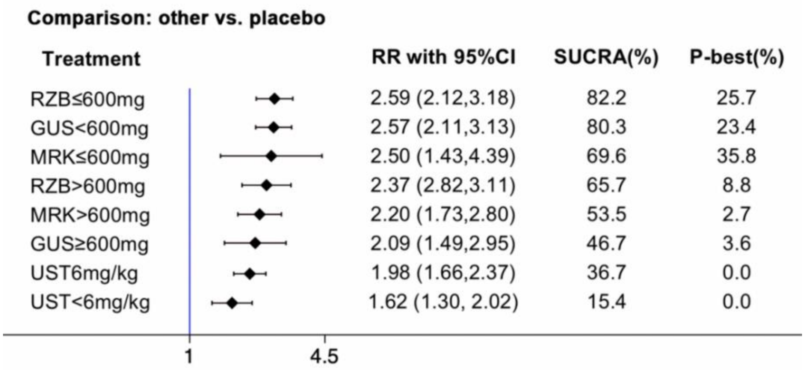

Note: SUCRA provides a cumulative distribution of a treatment's rankings, which quantifies the probability of it being among the most effective interventions. P-best is the probability of each intervention being ranked as best in the network. CD, Crohn’s disease; RR, relative risk. PBO, placebo, RZB, Risankizumab, MRK, Mirikizumab, GUS, Guselkumab, UST, Ustekinumab.

Supplementary Figure S5. Network heat plot for clinical response during induction.

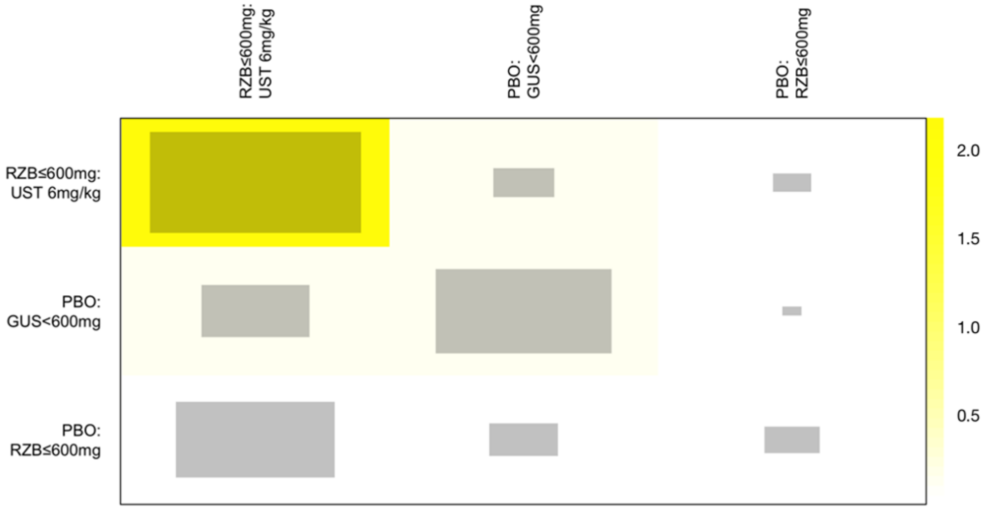

The network heat plot for clinical response reveals that most of the evidence is concentrated in the comparison between RZB ≤ 600 mg and UST 6 mg/kg, shown by the strong yellow intensity in the upper left quadrant, indicating robust direct data and high precision in this contrast. The moderate gray shading across PBO:GUS < 600 mg and PBO:RZB ≤ 600 mg suggests additional, but weaker, supportive links mainly derived from placebo-controlled studies. Overall, the evidence structure indicates that RZB-UST head-to-head data dominate the remission network, while other inter-biologic connections remain relatively sparse, implying that most of the transitivity in this network relies on indirect evidence mediated through placebo arms.

Supplementary Figure S6. Forest plot of Sensitivity analysis for inducing the clinical response among all participants, excluding ADVANCE.

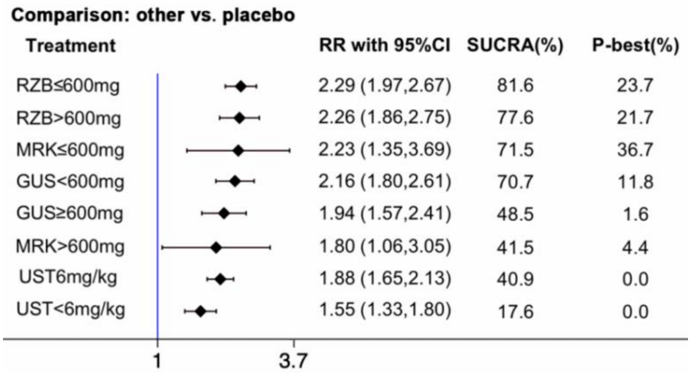

Note: SUCRA provides a cumulative distribution of a treatment's rankings, which quantifies the probability of it being among the most effective interventions. P-best is the probability of each intervention being ranked as best in the network. CD, Crohn's disease; RR, relative risk. PBO, placebo, RZB, Risankizumab, MRK, Mirikizumab, GUS, Guselkumab, UST, Ustekinumab.

Supplementary Figure S7. Forest plot of interventions' efficacy on inducing clinical and endoscopic outcomes with placebo as reference among previous bio-failure participants.

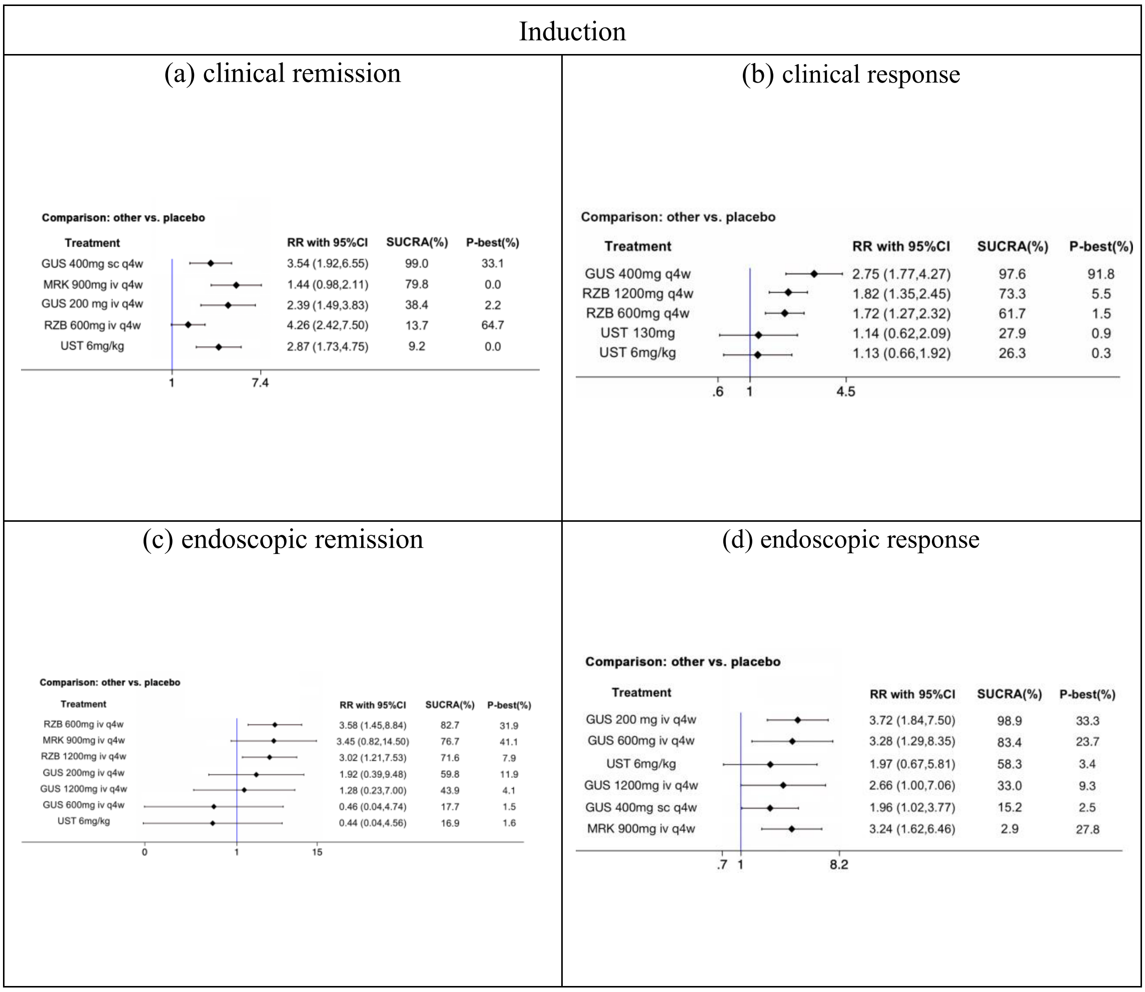

SUCRA provides a cumulative distribution of a treatment's rankings, which quantifies the probability of it being among the most effective interventions. P-best is the probability of each intervention being ranked as best in the network. SUCRA, surface under the cumulative ranking curve, P-best, probability of being the best, RR, relative risk, PBO, placebo, RZB, Risankizumab, MRK, Mirikizumab, GUS, Guselkumab, UST, Ustekinumab, q4w, every 4 weeks, q8w, every 8 weeks.

Supplementary Figure S8. Forest plot of interventions' efficacy on inducing and maintaining clinical and endoscopic outcomes with placebo as reference among previous bio-naive participants.

(a) clinical remission-induction

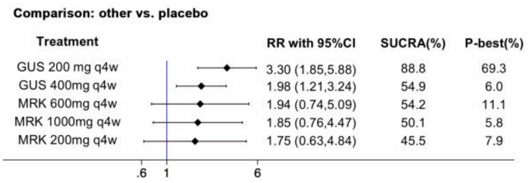

(b) clinical response-induction

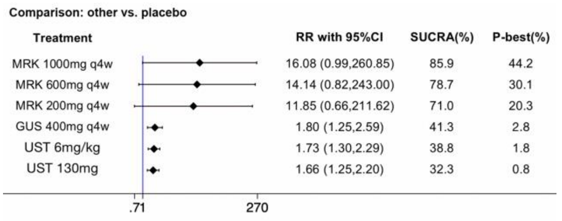

(c) endoscopic response-induction

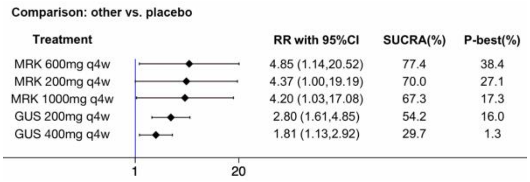

(d) clinical remission-maintenance

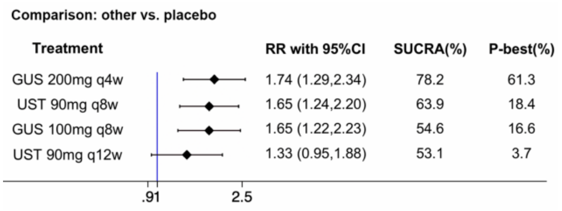

RZB, Risankizumab, MRK, Mirikizumab, GUS, Guselkumab, UST, Ustekinumab, q4w, every 4 weeks, q8w, every 8 weeks, q12w, every 12 weeks.

Supplementary Figure S9. Network heat plot for endoscopic remission during induction.

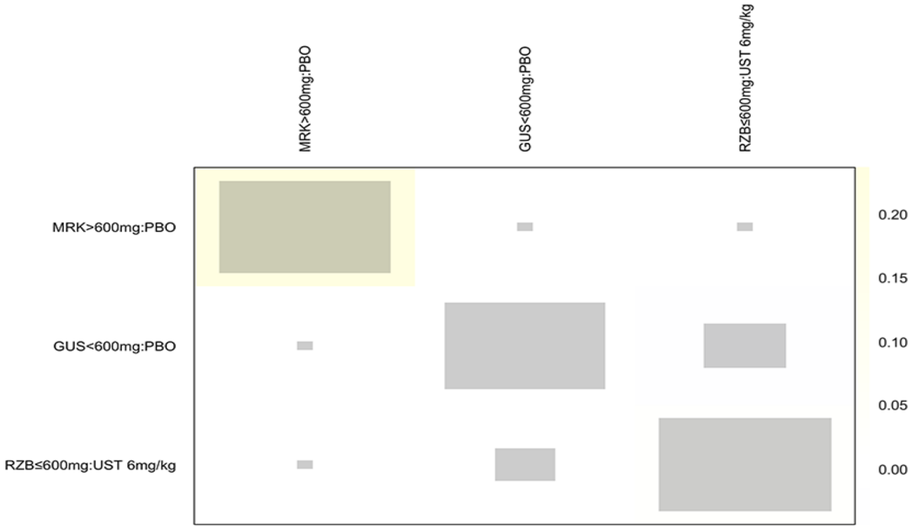

The contribution heat plot shows how much each direct comparison contributes to the network estimates of treatment effects. Darker colors represent greater contributions. In this analysis, the comparison MRK>600 mg vs PBO provides the largest contribution, particularly to the contrast RZB≤600 mg vs UST 6 mg/kg, indicating this pair of trials has a strong influence on the network estimates.

Supplementary Figure S10. Network heat plot for endoscopic response during induction.

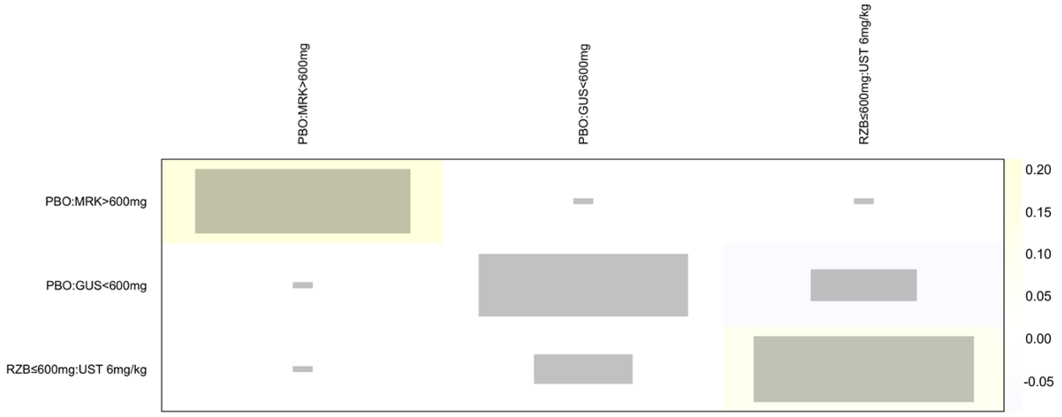

The network heat plot illustrates the relative contribution of direct and indirect evidence across treatment comparisons. The most prominent evidence originated from head-to-head trials comparing RZB≤600 mg + UST 6 mg/kg and RZB≤600 mg, as indicated by the darkest yellow cells. Moderate direct evidence was observed for comparisons involving PBO vs RZB≤600 mg and PBO vs GUS < 600 mg, whereas other treatment pairs relied mainly on indirect evidence through the network structure. Overall, the plot confirms a well-connected network with strong evidence concentration in RZB- and PBO-related nodes.

Supplementary Figure S11. Network heat plot for clinical remission during maintenance.

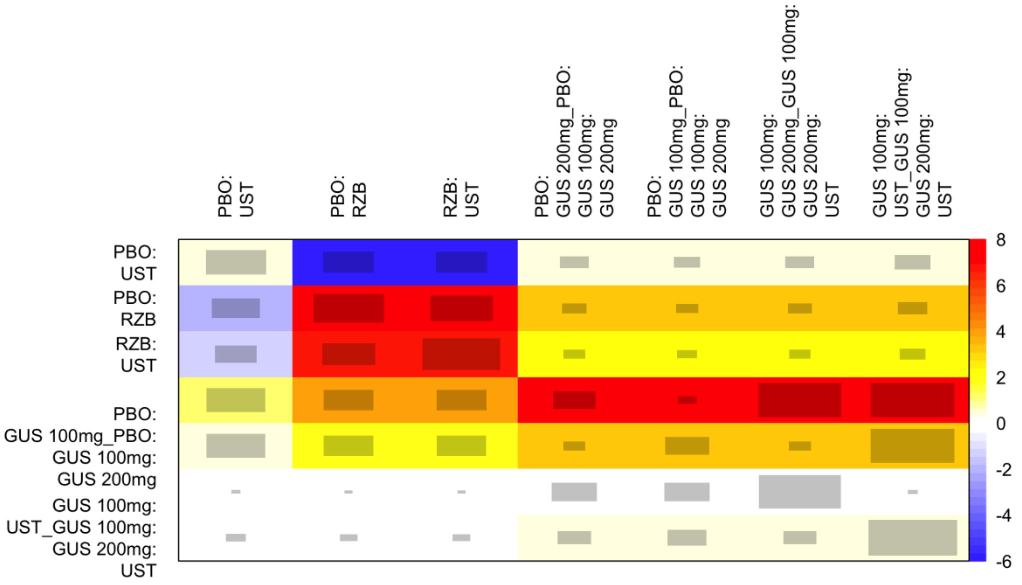

The plot shows that comparisons involving PBO:GUS 200 mg, PBO:GUS 100 mg, and RZB:UST exhibit the strongest direct evidence signals (red clusters), suggesting that these pairs serve as key evidence anchors within the network. The consistent yellow shading extending toward GUS 100 mg:UST and GUS 200 mg:UST indicates supplementary but less intensive links, supporting transitivity between UST- and GUS-based regimens. Conversely, blue or near-neutral areas highlight treatment contrasts that rely mainly on indirect inference-particularly among higher-dose regimens where head-to-head trials remain scarce. Overall, the network for clinical remission during maintenance demonstrates a well-connected but hierarchically unbalanced structure, in which placebo-controlled GUS trials dominate the evidence base, while direct UST-RZB or high-dose cross-comparisons remain limited. This uneven distribution implies that while the network provides sufficient linkage for global consistency testing, the precision of certain indirect estimates—especially between non-placebo comparators—should be interpreted with caution.

Supplementary Figure S12. Forest plot of sensitivity analysis for maintaining the clinical remission among all participants, excluding SEQUENCE and CERTIFI.

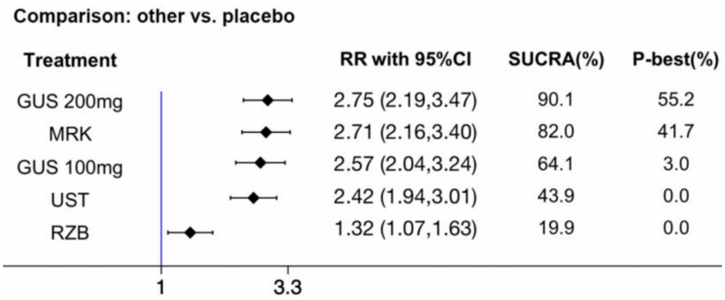

Note: SUCRA provides a cumulative distribution of a treatment's rankings, which quantifies the probability of it being among the most effective interventions. P-best is the probability of each intervention being ranked as best in the network. CD, Crohn's disease; RR, relative risk. PBO, placebo, RZB, Risankizumab, MRK, Mirikizumab, GUS, Guselkumab, UST, Ustekinumab.

Supplementary Figure S13. Network heat plot for clinical response during maintenance.

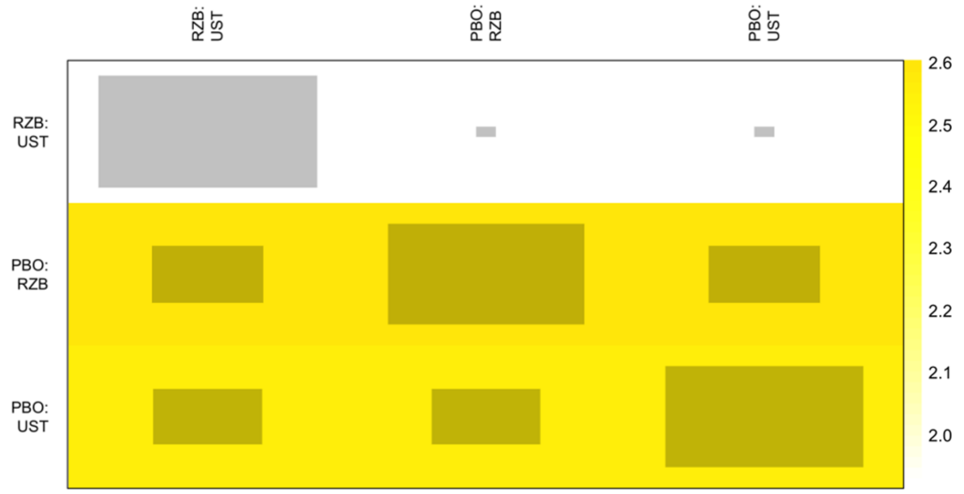

The network heat plot for clinical response during maintenance shows a structure dominated by placebo-controlled comparisons, as indicated by the strong yellow shading along the PBO:RZB and PBO:UST links. This pattern highlights that the majority of direct evidence arises from studies comparing risankizumab (RZB) and ustekinumab (UST) individually against placebo rather than against each other. The gray shading between RZB:UST suggests the existence of limited or lower-precision direct head-to-head data, implying that the relative efficacy estimates between these biologics rely largely on indirect evidence mediated through placebo arms. Overall, the heat distribution reflects a well-connected but placebo-centric evidence network, typical of biologic induction trials in Crohn's disease, where transitivity assumptions are critical for valid network inference.

Supplementary Figure S14. Forest plot of sensitivity analysis for maintaining the clinical response among all participants, excluding CERTIFI. 14

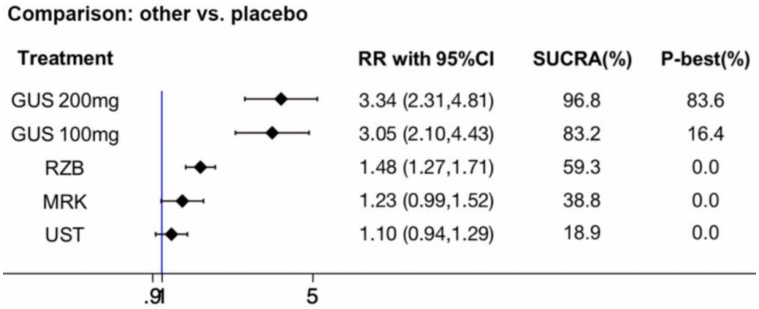

Note: SUCRA provides a cumulative distribution of a treatment's rankings, which quantifies the probability of it being among the most effective interventions. P-best is the probability of each intervention being ranked as best in the network. CD, Crohn's disease; RR, relative risk. PBO, placebo, RZB, Risankizumab, MRK, Mirikizumab, GUS, Guselkumab, UST, Ustekinumab.

Supplementary Figure S15. Forest plot of interventions' efficacy on maintaining clinical and endoscopic outcomes with placebo as reference among previous bio-failure participants. 15

Maintenance

(a) clinical remission

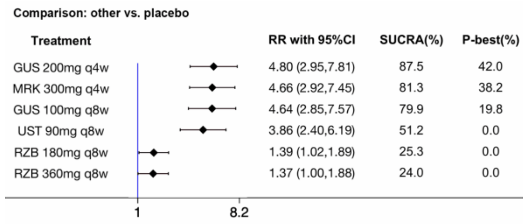

(b) endoscopic remission

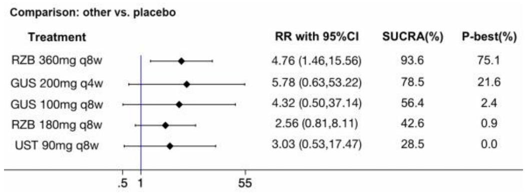

(c) endoscopic response

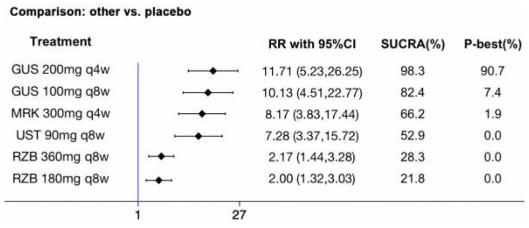

SUCRA provides a cumulative distribution of a treatment's rankings, which quantifies the probability of it being among the most effective interventions. P-best is the probability of each intervention being ranked as best in the network. SUCRA, surface under the cumulative ranking curve, P-best, probability of being the best, RR, relative risk, PBO, placebo, RZB, Risankizumab, MRK, Mirikizumab, GUS, Guselkumab, UST, Ustekinumab, q4w, every 4 weeks, q8w, every 8 weeks.

Supplementary Figure S16. Network heat plot for endoscopic remission during maintenance.

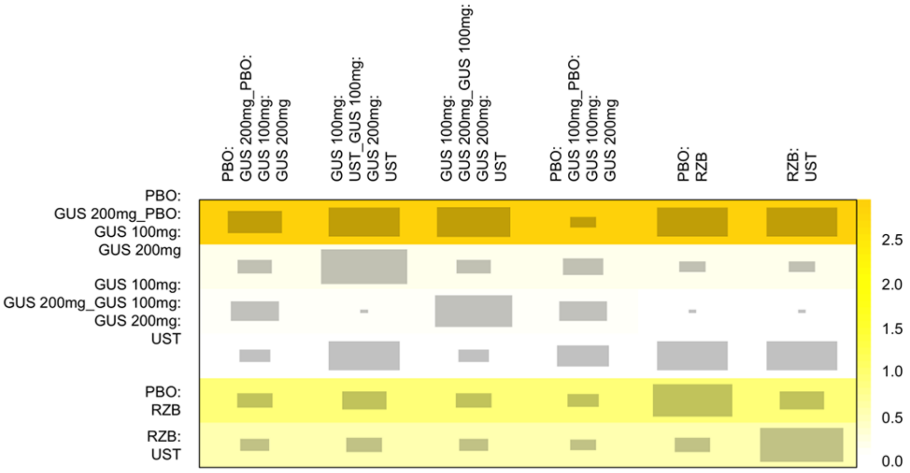

The network heat plot for endoscopic remission during maintenance reveals a complex yet unbalanced structure, characterized by dense clusters of direct evidence among guselkumab (GUS) dose regimens and relatively sparse cross-biologic comparisons. The strong yellow shading between PBO:GUS 100 mg and GUS 200 mg combinations indicates a high concentration of within-drug trials exploring multiple GUS dosing strategies, suggesting robust internal consistency within this treatment family. In contrast, the lighter shading along PBO:RZB and RZB:UST links reflects fewer direct comparisons, implying that efficacy inference across biologic classes—particularly between risankizumab and ustekinumab—relies mainly on indirect evidence through shared placebo arms. This pattern underscores that the induction network for endoscopic remission is dose-centric rather than class-comparative, strengthening conclusions about intra-drug dose–response but limiting the precision of inter-biologic ranking. Overall, the heat distribution reflects a hierarchically layered network, where guselkumab dose studies dominate the evidence intensity, providing strong internal validity but necessitating cautious interpretation of cross-class comparisons.

Supplementary Figure S17. Forest plot of sensitivity analysis for maintaining the endoscopic remission among all participants, excluding FORTIFY.

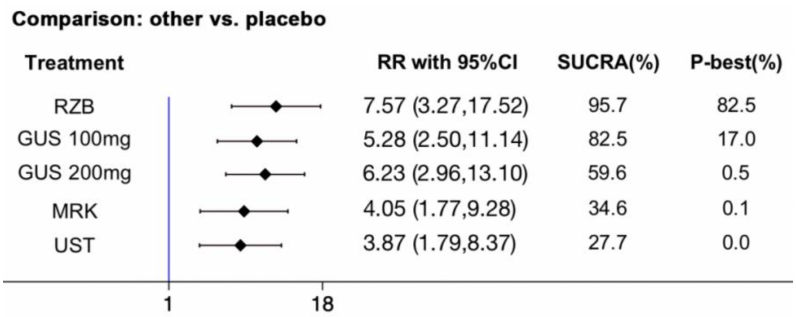

Note: SUCRA provides a cumulative distribution of a treatment's rankings, which quantifies the probability of it being among the most effective interventions. P-best is the probability of each intervention being ranked as best in the network. CD, Crohn’s disease; RR, relative risk. PBO, placebo, RZB, Risankizumab, MRK, Mirikizumab, GUS, Guselkumab, UST, Ustekinumab.

Supplementary Figure S18. Network heat plot for endoscopic response during maintenance.

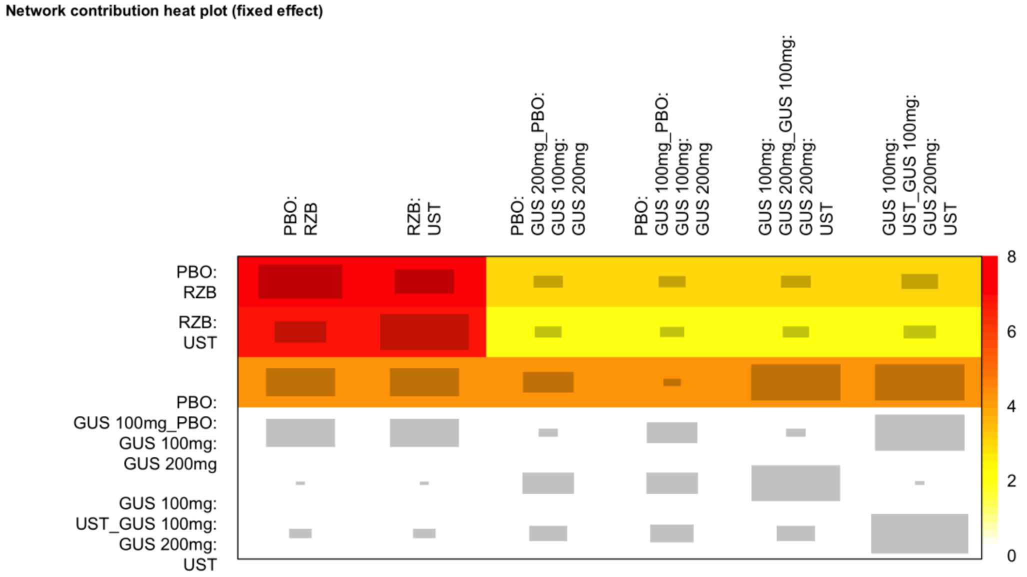

The cells corresponding to the comparison between PBO:RZB and RZB:UST appear in the deepest shade of red, indicating the strongest direct evidence between these two groups—that is, the highest number of related studies and the most comprehensive comparative information. Additionally, continuous yellow to light orange areas are visible between PBO, GUS 100 mg, GUS 200 mg, and UST, indicating relatively dense direct comparative relationships among these treatments that form the primary connective structure within the network. Gray or white areas denote nodes lacking direct evidence or exhibiting weaker connections.

Supplementary Figure S19. Forest plot of sensitivity analysis for maintaining the endoscopic response among all participants, excluding FORTIFY.

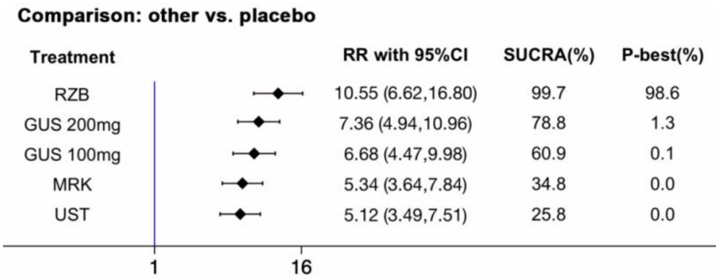

Note: SUCRA provides a cumulative distribution of a treatment's rankings, which quantifies the probability of it being among the most effective interventions. P-best is the probability of each intervention being ranked as best in the network. CD, Crohn's disease; RR, relative risk. PBO, placebo, RZB, Risankizumab, MRK, Mirikizumab, GUS, Guselkumab, UST, Ustekinumab.

Supplementary Figure S20. Forest plot of dose-stratified traditional Meta-Analysis comparing efficacy of individual IL-23p19 inhibitor doses against placebo on clinical remission during induction.

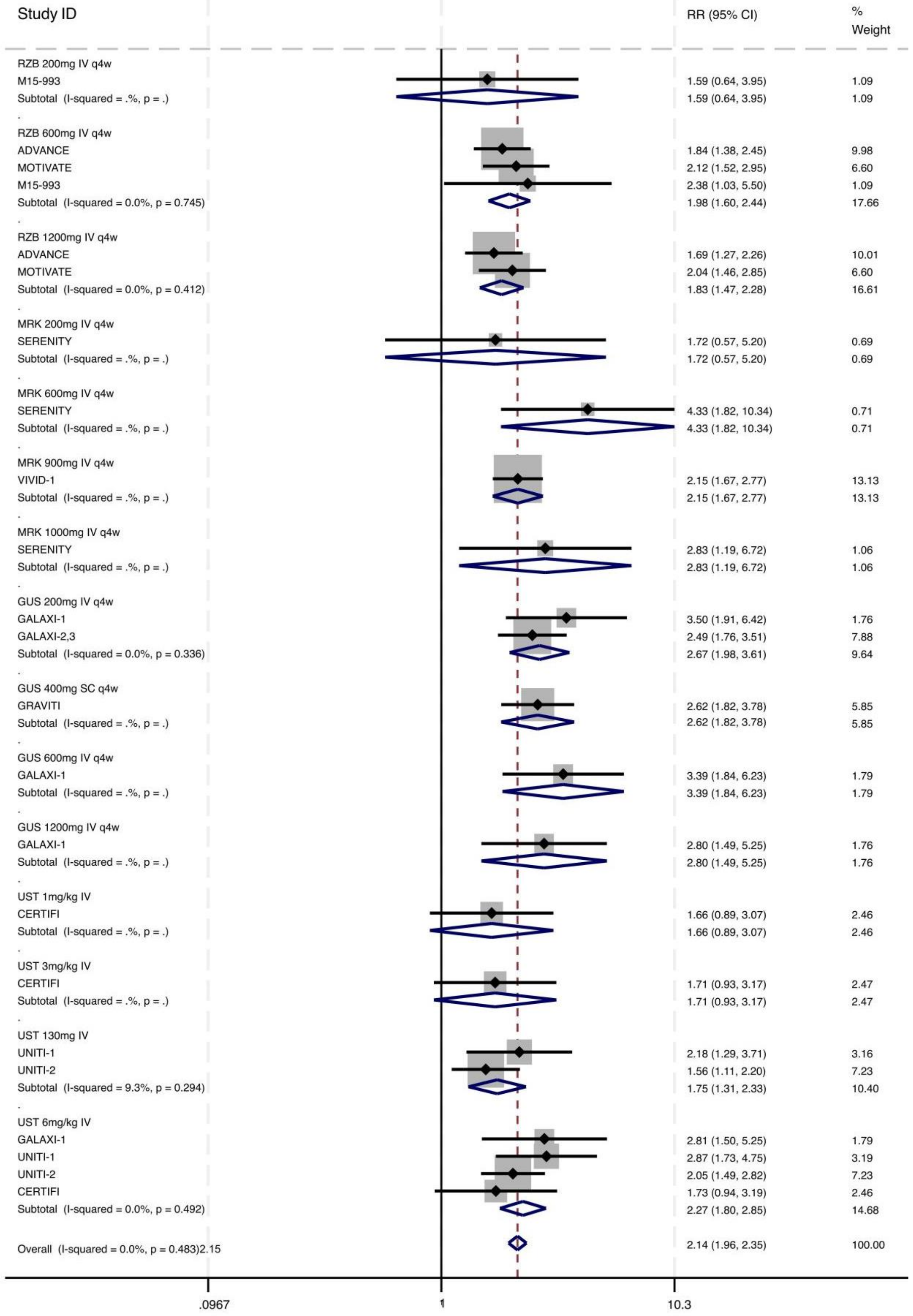

Traditional pairwise meta-analysis stratified strictly by identical individual doses to evaluating the efficacy of individual induction dosing regimens for each IL-23/IL-12/23 inhibitor versus placebo in patients with moderate-to-severe Crohn's disease. Risk ratios (RR) with 95% confidence intervals (CI) were pooled using a fixed-effect (Mantel-Haenszel) model, with a random-effects (M-H heterogeneity) model applied when statistical heterogeneity (I^2) exceeded 50%. RR > 1 favors the active agent. PBO, placebo; RR, risk ratio; CI, confidence interval; I^2, index of between-study inconsistency; M-H , Mantel-Haenszel.

Supplementary Figure S21. Forest plot of dose-stratified traditional Meta-Analysis comparing efficacy of individual IL-23p19 inhibitor doses against placebo on clinical response during induction.

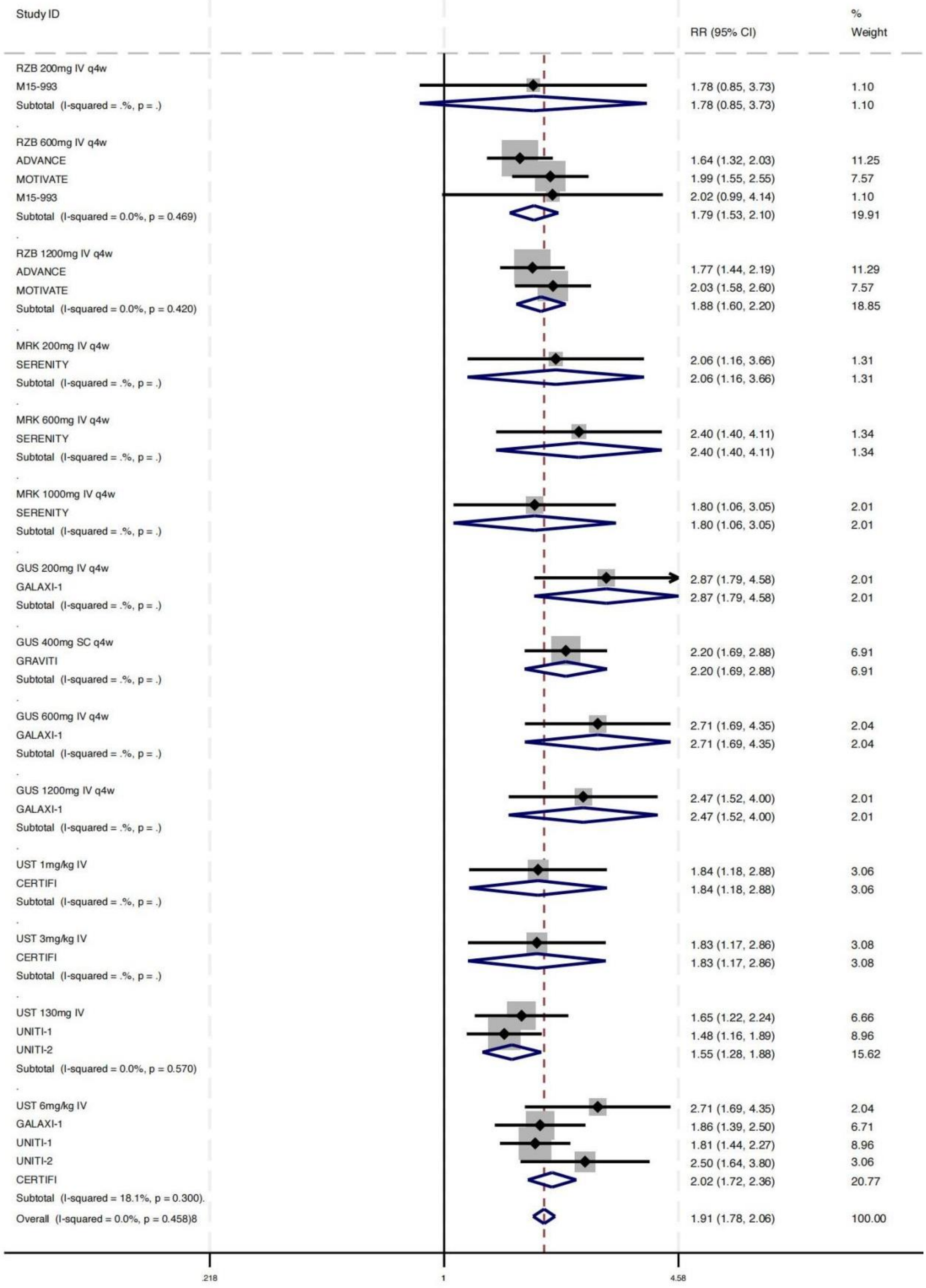

Traditional pairwise meta-analysis stratified strictly by identical individual doses to evaluating the efficacy of individual induction dosing regimens for each IL-23/IL-12/23 inhibitor versus placebo in patients with moderate-to-severe Crohn's disease. Risk ratios (RR) with 95% confidence intervals (CI) were pooled using a fixed-effect (Mantel-Haenszel) model, with a random-effects (M-H heterogeneity) model applied when statistical heterogeneity (I^2) exceeded 50%. RR > 1 favors the active agent. PBO, placebo; RR, risk ratio; CI, confidence interval; I^2, index of between-study inconsistency; M-H , Mantel-Haenszel.

Supplementary Figure S22. Forest plot of dose-stratified traditional Meta-Analysis comparing efficacy of individual IL-23p19 inhibitor doses against placebo on endoscopic remission during induction.

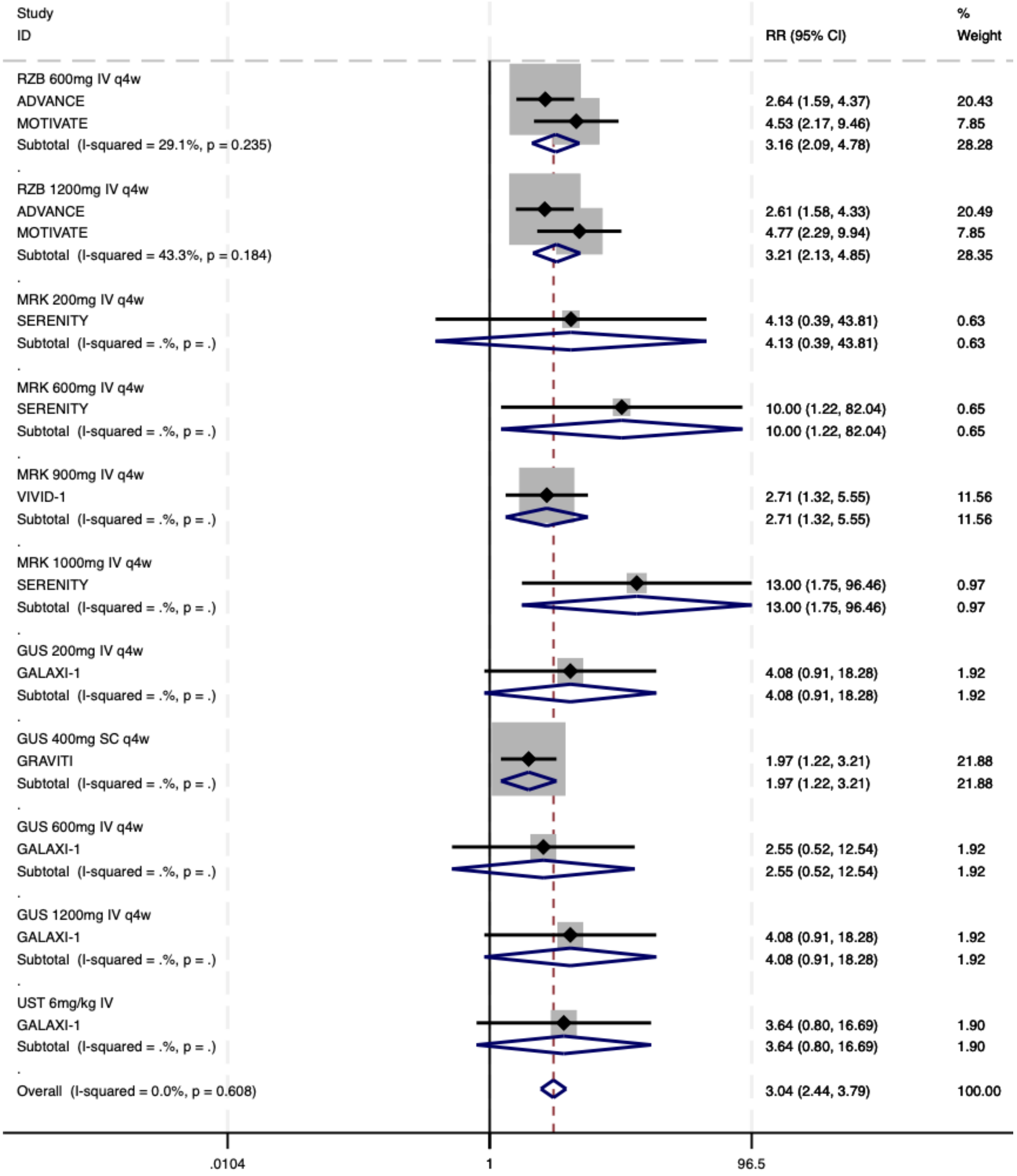

Traditional pairwise meta-analysis stratified strictly by identical individual doses to evaluating the efficacy of individual induction dosing regimens for each IL-23/IL-12/23 inhibitor versus placebo in patients with moderate-to-severe Crohn's disease. Risk ratios (RR) with 95% confidence intervals (CI) were pooled using a fixed-effect (Mantel-Haenszel) model, with a random-effects (M-H heterogeneity) model applied when statistical heterogeneity (I^2) exceeded 50%. RR > 1 favors the active agent. PBO, placebo; RR, risk ratio; CI, confidence interval; I^2, index of between-study inconsistency; M-H , Mantel-Haenszel.

Supplementary Figure S23. Forest plot of dose-stratified traditional Meta-Analysis comparing efficacy of individual IL-23p19 inhibitor doses against placebo on endoscopic response during induction.

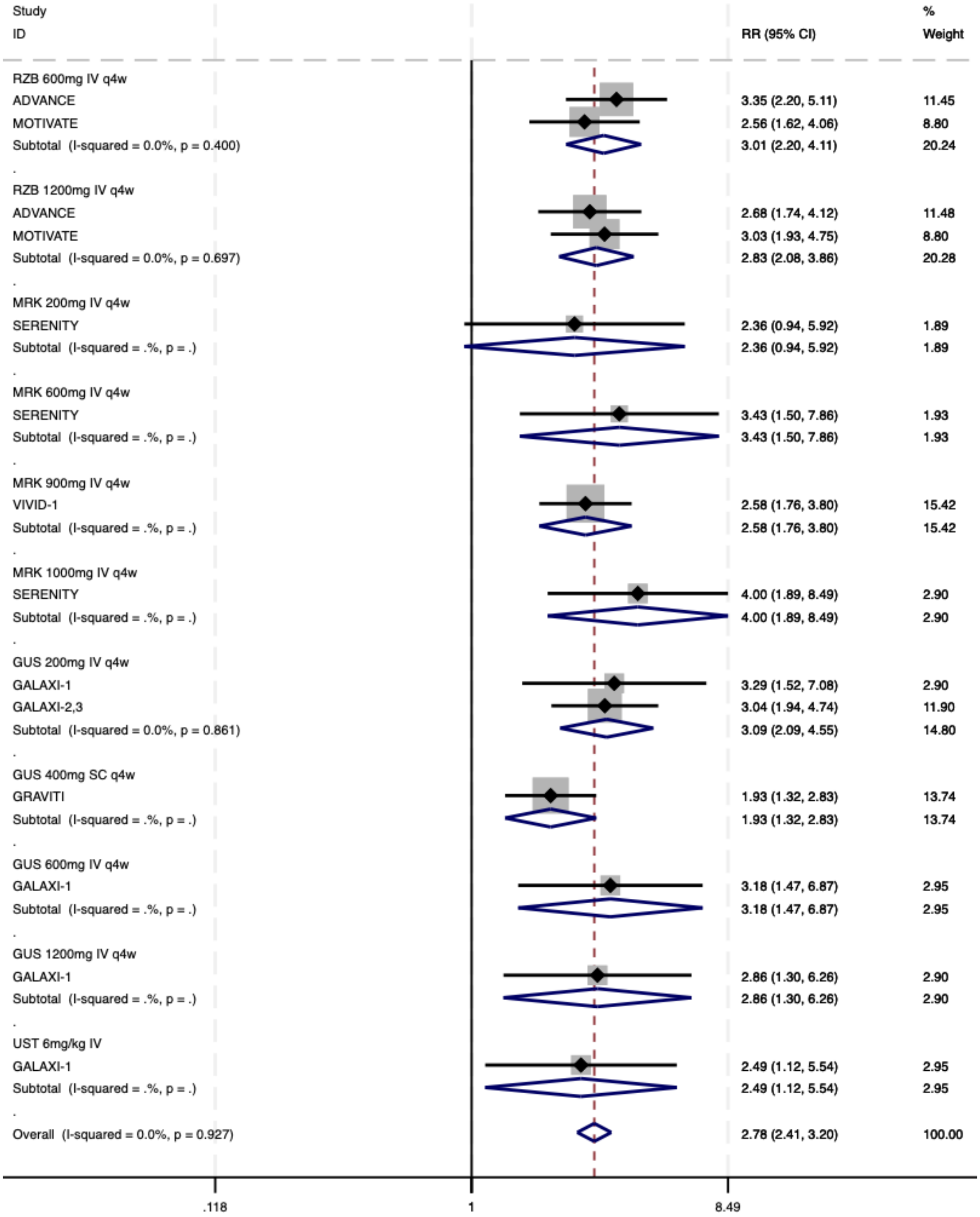

Traditional pairwise meta-analysis stratified strictly by identical individual doses to evaluating the efficacy of individual induction dosing regimens for each IL-23/IL-12/23 inhibitor versus placebo in patients with moderate-to-severe Crohn's disease. Risk ratios (RR) with 95% confidence intervals (CI) were pooled using a fixed-effect (Mantel-Haenszel) model, with a random-effects (M-H heterogeneity) model applied when statistical heterogeneity (I^2) exceeded 50%. RR > 1 favors the active agent. PBO, placebo; RR, risk ratio; CI, confidence interval; I^2, index of between-study inconsistency; M-H , Mantel-Haenszel.

Supplementary Figure S24. Forest plot of dose-stratified traditional Meta-Analysis comparing efficacy of individual IL-23p19 inhibitor doses against placebo on clinical remission during maintenance.

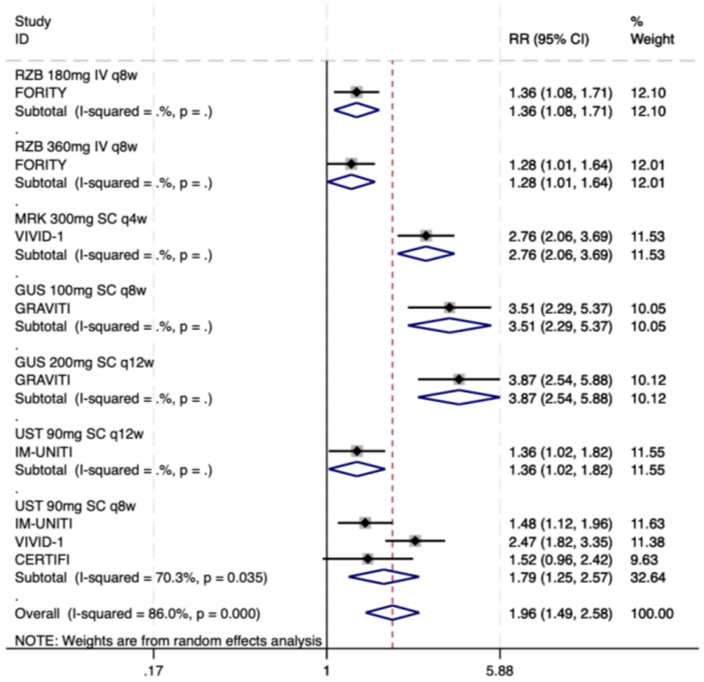

Traditional pairwise meta-analysis stratified strictly by identical individual doses to evaluating the efficacy of individual induction dosing regimens for each IL-23/IL-12/23 inhibitor versus placebo in patients with moderate-to-severe Crohn's disease. Risk ratios (RR) with 95% confidence intervals (CI) were pooled using a fixed-effect (Mantel-Haenszel) model, with a random-effects (M-H heterogeneity) model applied when statistical heterogeneity (I^2) exceeded 50%. RR > 1 favors the active agent. PBO, placebo; RR, risk ratio; CI, confidence interval; I^2, index of between-study inconsistency; M-H , Mantel-Haenszel.

Supplementary Figure S25. Forest plot of dose-stratified traditional Meta-Analysis comparing efficacy of individual IL-23p19 inhibitor doses against ustekinumab on clinical remission during maintenance.

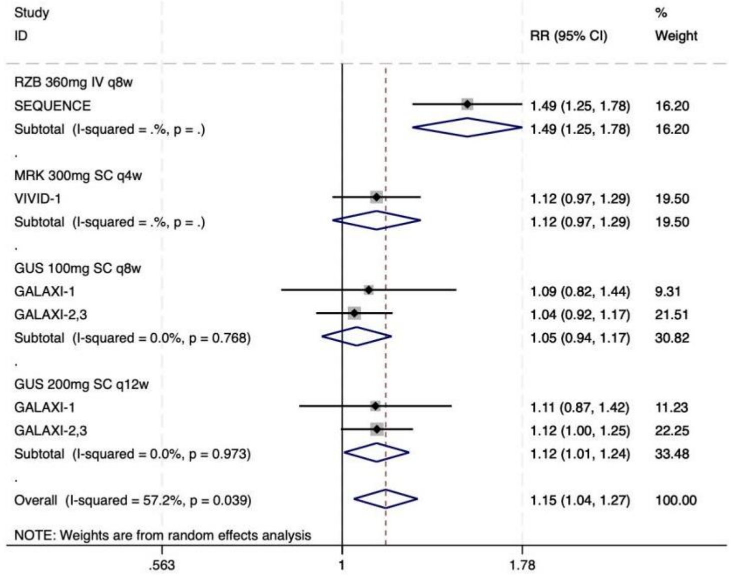

Traditional pairwise meta-analysis stratified strictly by identical individual doses to evaluating the efficacy of individual induction dosing regimens for each IL-23/IL-12/23 inhibitor versus placebo in patients with moderate-to-severe Crohn's disease. Risk ratios (RR) with 95% confidence intervals (CI) were pooled using a fixed-effect (Mantel-Haenszel) model, with a random-effects (M-H heterogeneity) model applied when statistical heterogeneity (I^2) exceeded 50%. RR > 1 favors the active agent. PBO, placebo; RR, risk ratio; CI, confidence interval; I^2, index of between-study inconsistency; M-H , Mantel-Haenszel.

Supplementary Figure S26. Forest plot of dose-stratified traditional Meta-Analysis comparing efficacy of individual IL-23p19 inhibitor doses against placebo on clinical response during maintenance.

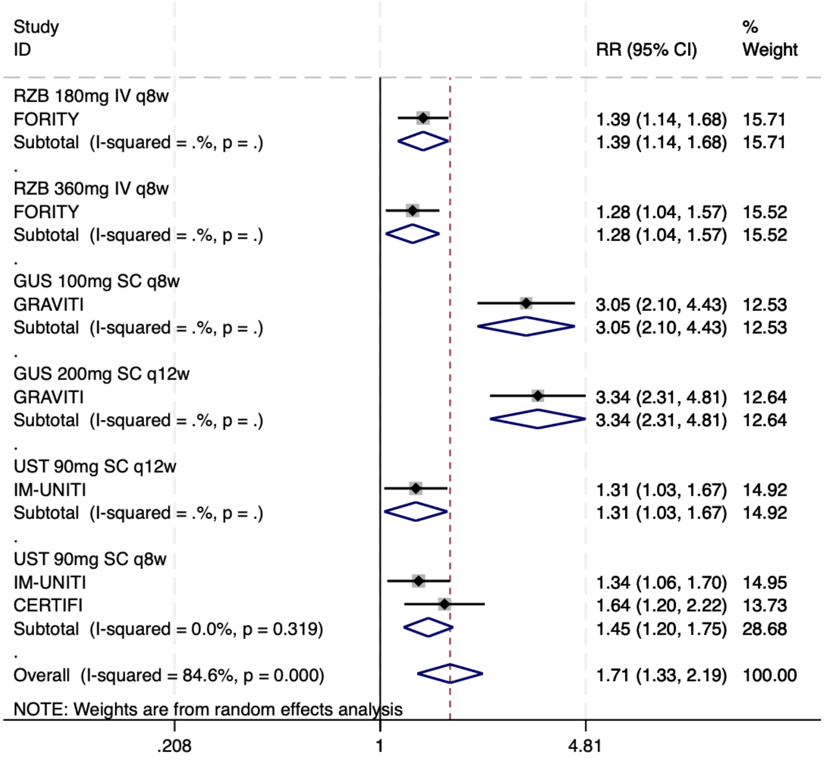

Traditional pairwise meta-analysis stratified strictly by identical individual doses to evaluating the efficacy of individual induction dosing regimens for each IL-23/IL-12/23 inhibitor versus placebo in patients with moderate-to-severe Crohn's disease. Risk ratios (RR) with 95% confidence intervals (CI) were pooled using a fixed-effect (Mantel-Haenszel) model, with a random-effects (M-H heterogeneity) model applied when statistical heterogeneity (I^2) exceeded 50%. RR > 1 favors the active agent. PBO, placebo; RR, risk ratio; CI, confidence interval; I^2, index of between-study inconsistency; M-H , Mantel-Haenszel.

Supplementary Figure S27. Forest plot of dose-stratified traditional Meta-Analysis comparing efficacy of individual IL-23p19 inhibitor doses against placebo on endoscopic remission during maintenance.

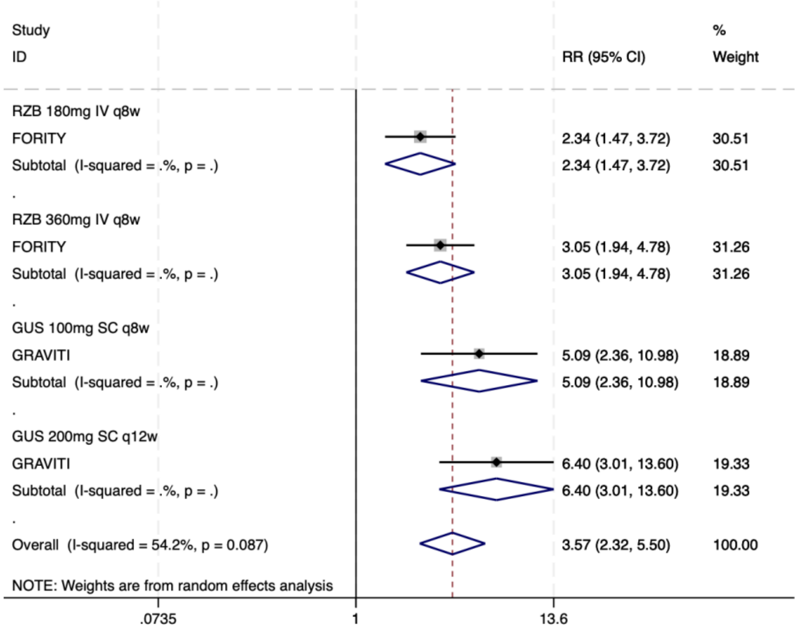

Traditional pairwise meta-analysis stratified strictly by identical individual doses to evaluating the efficacy of individual induction dosing regimens for each IL-23/IL-12/23 inhibitor versus placebo in patients with moderate-to-severe Crohn's disease. Risk ratios (RR) with 95% confidence intervals (CI) were pooled using a fixed-effect (Mantel-Haenszel) model, with a random-effects (M-H heterogeneity) model applied when statistical heterogeneity (I^2) exceeded 50%. RR > 1 favors the active agent. PBO, placebo; RR, risk ratio; CI, confidence interval; I^2, index of between-study inconsistency; M-H , Mantel-Haenszel.

Supplementary Figure S28. Forest plot of dose-stratified traditional Meta-Analysis comparing efficacy of individual IL-23p19 inhibitor doses against ustekinumab on endoscopic remission during maintenance.

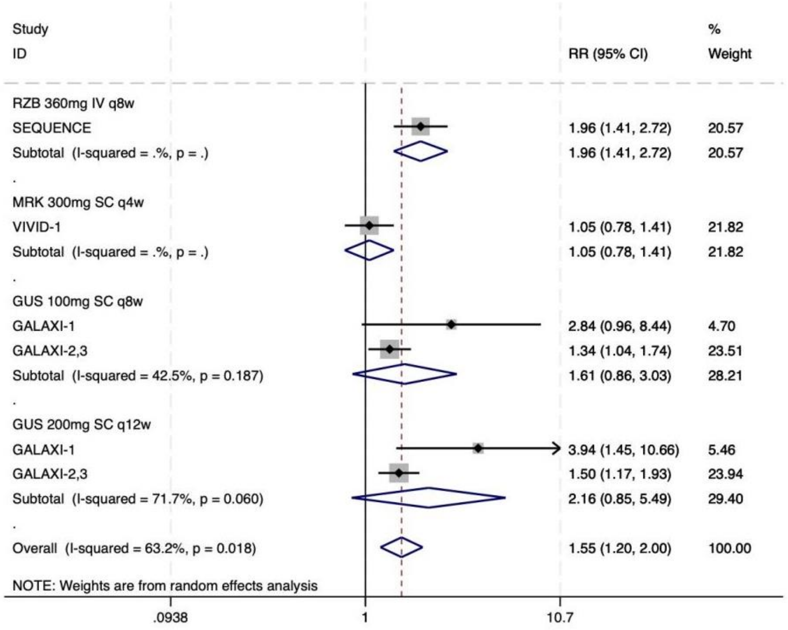

Traditional pairwise meta-analysis stratified strictly by identical individual doses to evaluating the efficacy of individual induction dosing regimens for each IL-23/IL-12/23 inhibitor versus placebo in patients with moderate-to-severe Crohn's disease. Risk ratios (RR) with 95% confidence intervals (CI) were pooled using a fixed-effect (Mantel-Haenszel) model, with a random-effects (M-H heterogeneity) model applied when statistical heterogeneity (I^2) exceeded 50%. RR > 1 favors the active agent. PBO, placebo; RR, risk ratio; CI, confidence interval; I^2, index of between-study inconsistency; M-H , Mantel-Haenszel.

Supplementary Figure S29. Forest plot of dose-stratified traditional Meta-Analysis comparing efficacy of individual IL-23p19 inhibitor doses against placebo on endoscopic response during maintenance.

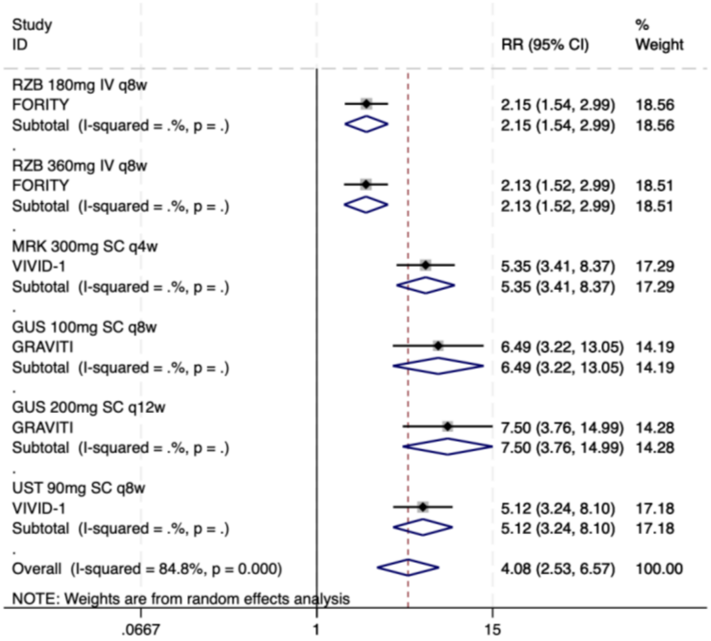

Traditional pairwise meta-analysis stratified strictly by identical individual doses to evaluating the efficacy of individual induction dosing regimens for each IL-23/IL-12/23 inhibitor versus placebo in patients with moderate-to-severe Crohn's disease. Risk ratios (RR) with 95% confidence intervals (CI) were pooled using a fixed-effect (Mantel-Haenszel) model, with a random-effects (M-H heterogeneity) model applied when statistical heterogeneity (I^2) exceeded 50%. RR > 1 favors the active agent. PBO, placebo; RR, risk ratio; CI, confidence interval; I^2, index of between-study inconsistency; M-H , Mantel-Haenszel.

Supplementary Figure S30. Forest plot of dose-stratified traditional Meta-Analysis comparing efficacy of individual IL-23p19 inhibitor doses against ustekinumab on endoscopic response during maintenance.

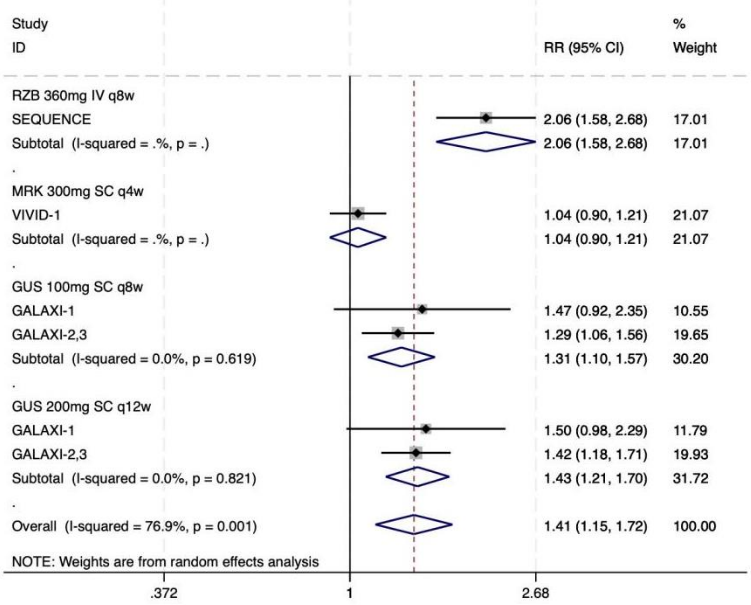

Traditional pairwise meta-analysis stratified strictly by identical individual doses to evaluating the efficacy of individual induction dosing regimens for each IL-23/IL-12/23 inhibitor versus placebo in patients with moderate-to-severe Crohn's disease. Risk ratios (RR) with 95% confidence intervals (CI) were pooled using a fixed-effect (Mantel-Haenszel) model, with a random-effects (M-H heterogeneity) model applied when statistical heterogeneity (I^2) exceeded 50%. RR > 1 favors the active agent. PBO, placebo; RR, risk ratio; CI, confidence interval; I^2, index of between-study inconsistency; M-H , Mantel-Haenszel.

Supplementary Figure S31. Forest plot of induction clinical remission by drug and assessment time point: a traditional Meta-Analysis stratified by induction duration.

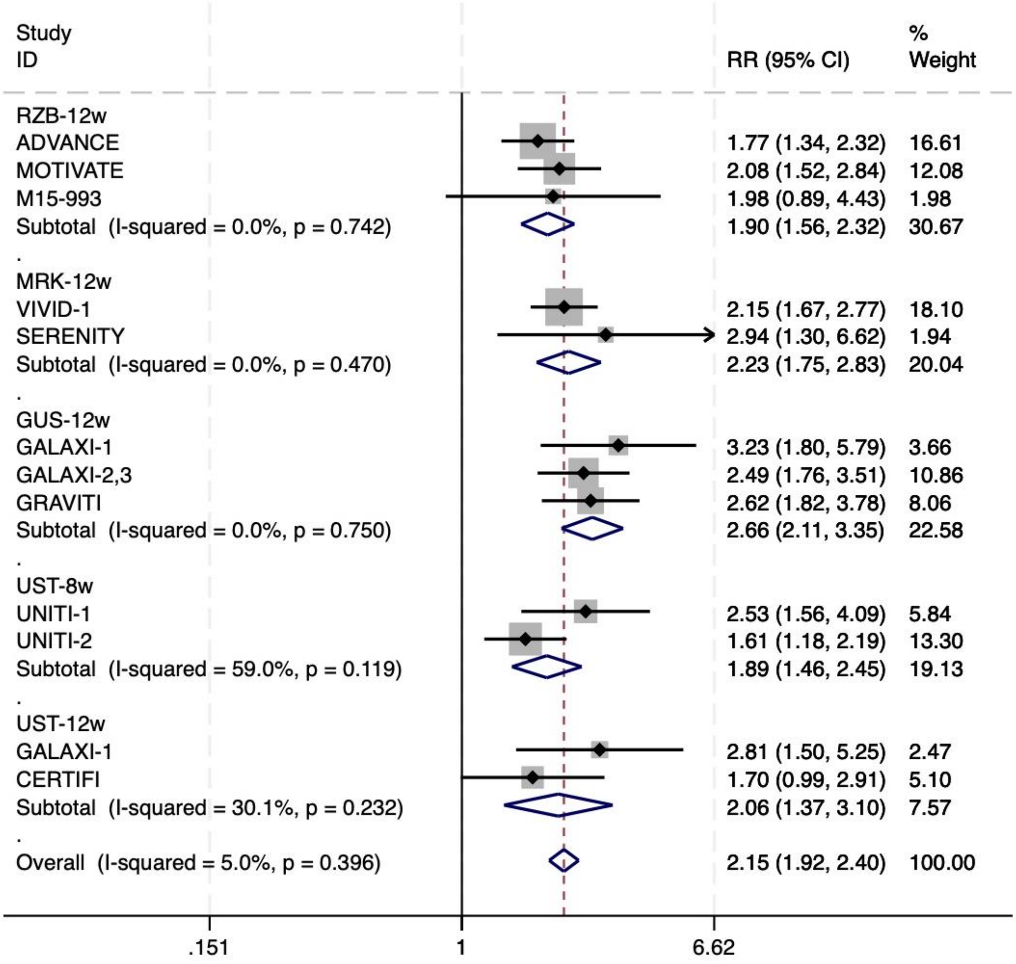

Pairwise meta-analysis of clinical remission rates at the end of induction (week 8 or week 12) for each IL-23/IL-12/23 inhibitor versus placebo in patients with moderately to severely active Crohn's disease. Studies were grouped by the same drug and same induction duration to examine the consistency of treatment effects across different time windows. Risk ratios (RR) with 95% confidence intervals (CI) were pooled using a fixed-effect (Mantel-Haenszel) model unless heterogeneity (I^2) exceeded 50%, in which case a random-effects (Mantel-Haenszel with heterogeneity) model was applied. The size of each square reflects the study weight, and horizontal lines represent the 95% CI. The diamond indicates the pooled estimate for each subgroup. PBO, placebo; RR, risk ratio; CI, confidence interval, I^2 , measure of inconsistency; M-H, Mantel-Haenszel.

Supplementary Figure S32. Forest plot of induction clinical response by drug and assessment time point: a traditional Meta-Analysis stratified by induction duration.

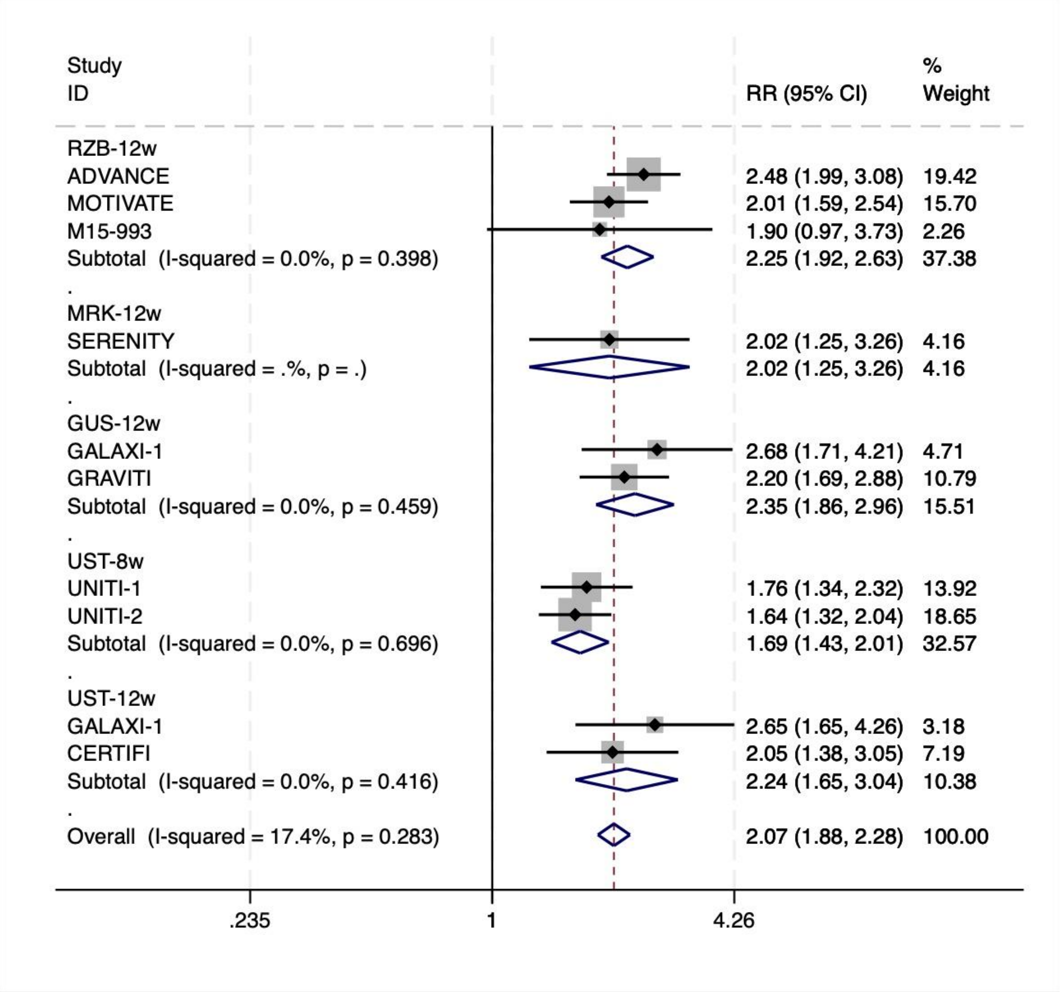

Pairwise meta-analysis of clinical response rates at the end of induction (week 8 or week 12) for each IL-23/IL-12/23 inhibitor versus placebo in patients with moderately to severely active Crohn's disease. Studies were grouped by the same drug and same induction duration to examine the consistency of treatment effects across different time windows. Risk ratios (RR) with 95% confidence intervals (CI) were pooled using a fixed-effect (Mantel-Haenszel) model unless heterogeneity (I^2) exceeded 50%, in which case a random-effects (Mantel-Haenszel with heterogeneity) model was applied. The size of each square reflects the study weight, and horizontal lines represent the 95% CI. The diamond indicates the pooled estimate for each subgroup. PBO, placebo; RR, risk ratio; CI, confidence interval, I^2 , measure of inconsistency; M-H, Mantel-Haenszel.

Supplementary Figure S33. Forest plot of induction endoscopic remission by drug and assessment time point: a traditional Meta-Analysis stratified by induction duration.

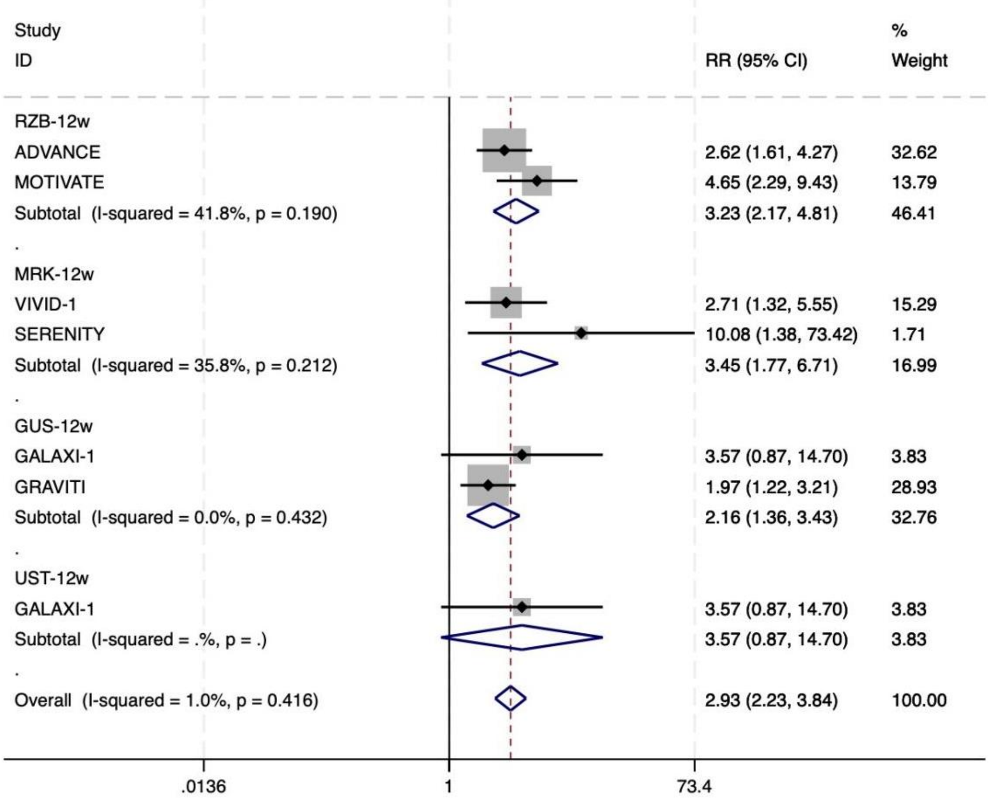

Pairwise meta-analysis of endoscopic remission rates at the end of induction for each IL-23/IL-12/23 inhibitor versus placebo in patients with moderately to severely active Crohn's disease. Studies were grouped by the same drug and same induction duration to examine the consistency of treatment effects across different time windows. Risk ratios (RR) with 95% confidence intervals (CI) were pooled using a fixed-effect (Mantel-Haenszel) model unless heterogeneity (I^2) exceeded 50%, in which case a random-effects (Mantel-Haenszel with heterogeneity) model was applied. The size of each square reflects the study weight, and horizontal lines represent the 95% CI. The diamond indicates the pooled estimate for each subgroup. PBO, placebo; RR, risk ratio; CI, confidence interval, I^2 , measure of inconsistency; M-H, Mantel-Haenszel.

Supplementary Figure S34. Forest plot of induction endoscopic response by drug and assessment time point: a traditional Meta-Analysis stratified by induction duration.

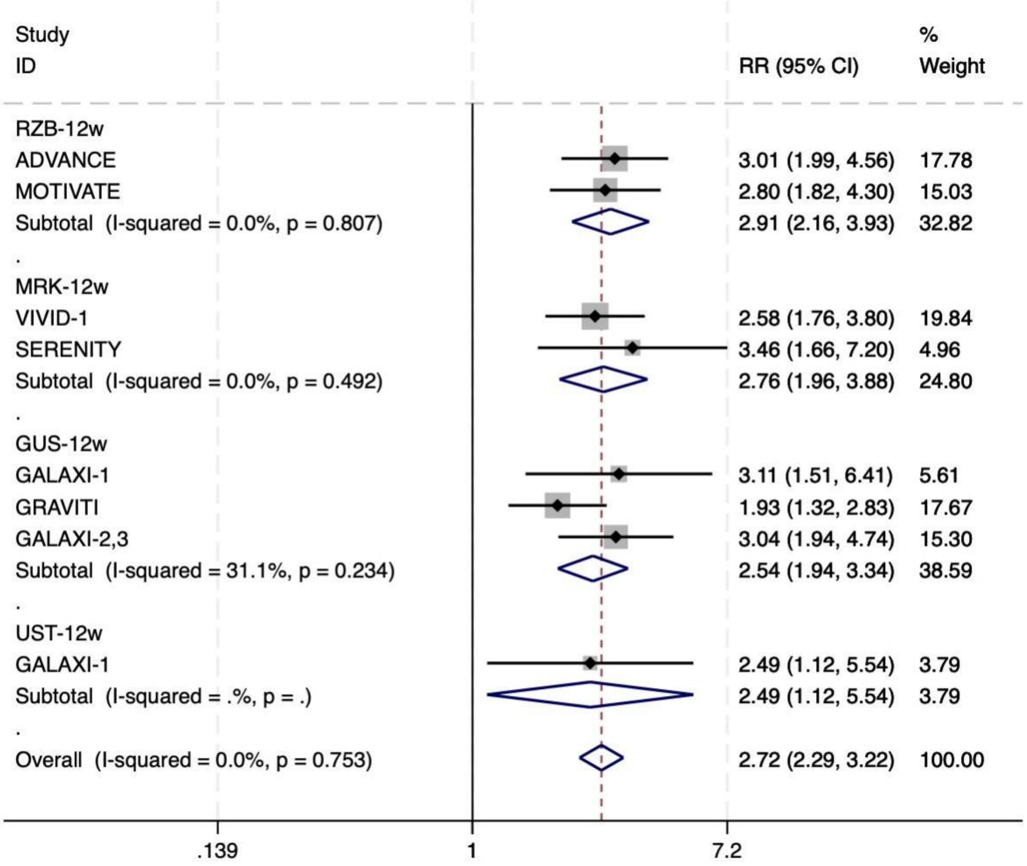

Pairwise meta-analysis of endoscopic response rates at the end of induction for each IL-23/IL-12/23 inhibitor versus placebo in patients with moderately to severely active Crohn's disease. Studies were grouped by the same drug and same induction duration to examine the consistency of treatment effects across different time windows. Risk ratios (RR) with 95% confidence intervals (CI) were pooled using a fixed-effect (Mantel-Haenszel) model unless heterogeneity (I^2) exceeded 50%, in which case a random-effects (Mantel-Haenszel with heterogeneity) model was applied. The size of each square reflects the study weight, and horizontal lines represent the 95% CI. The diamond indicates the pooled estimate for each subgroup. PBO, placebo; RR, risk ratio; CI, confidence interval, I^2 , measure of inconsistency; M-H, Mantel-Haenszel.

Supplementary Figure S35. Risk of bias assessment.

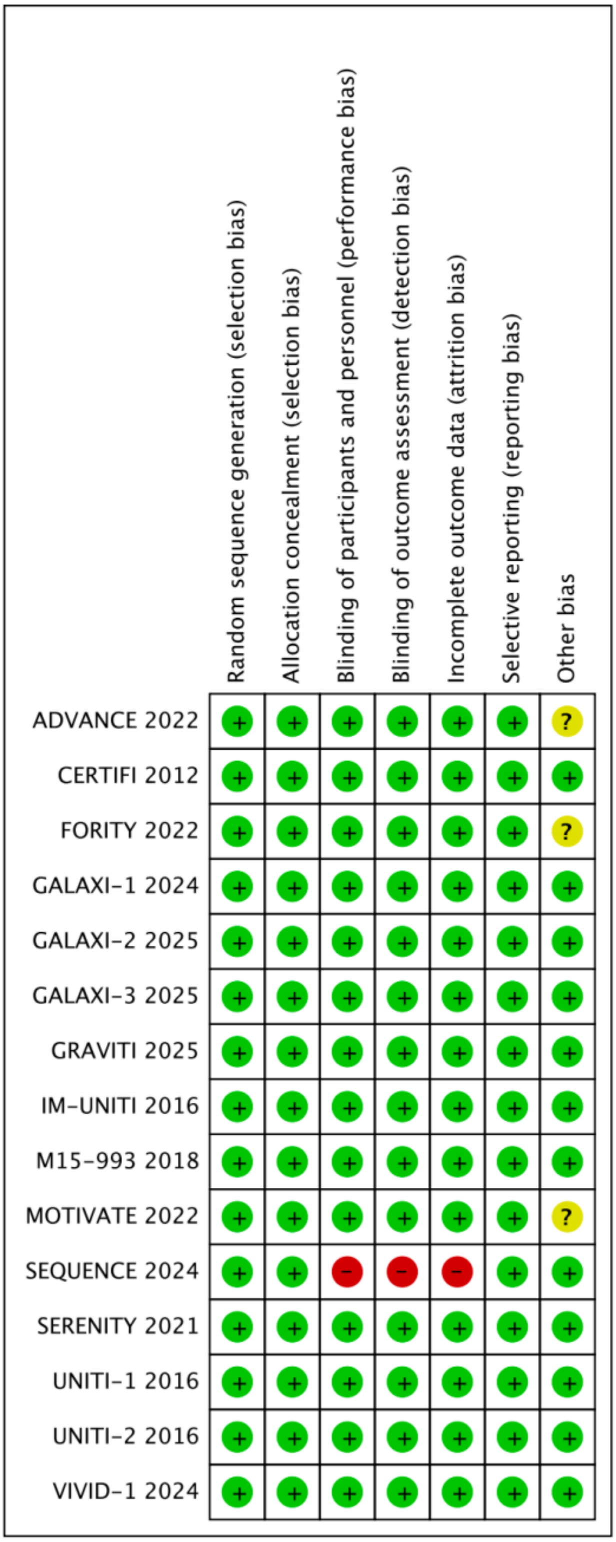

(a) Risk of bias graph

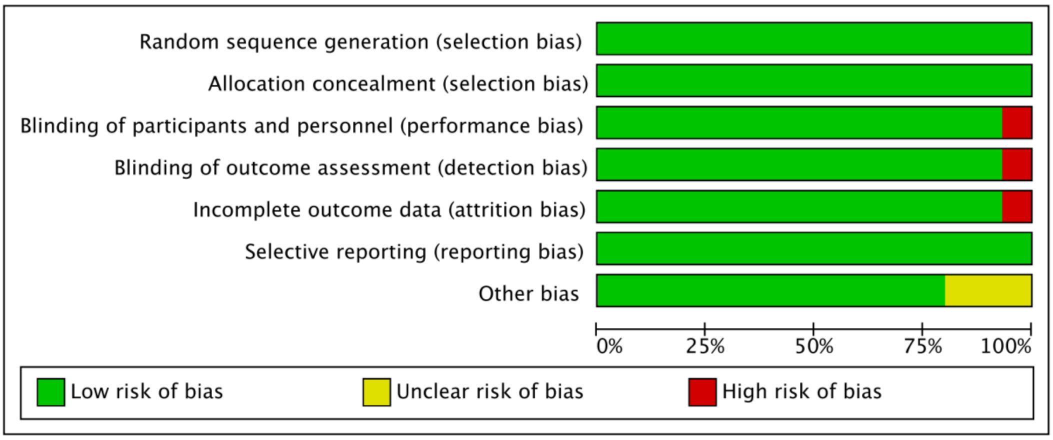

(b) Risk of bias summary

(a) Risk of bias graph. Overview of the methodological quality for each included randomized controlled trial across seven bias domains. Rows represent individual studies; columns represent bias domains. Symbols indicate the judged risk: green (+) for low risk, yellow (?) for unclear risk, and red (–) for high risk. (b) Risk of bias summary. Aggregate presentation of risk of bias judgments across all studies, expressed as percentages per domain. Each stacked bar sums to 100%. Colors represent green = low risk, yellow = unclear risk, red = high risk.

Supplementary Figure S36. SUCRA cumulative rank curves of Sensitivity analysis for inducing the clinical remission among all participants, excluding ADVANCE.

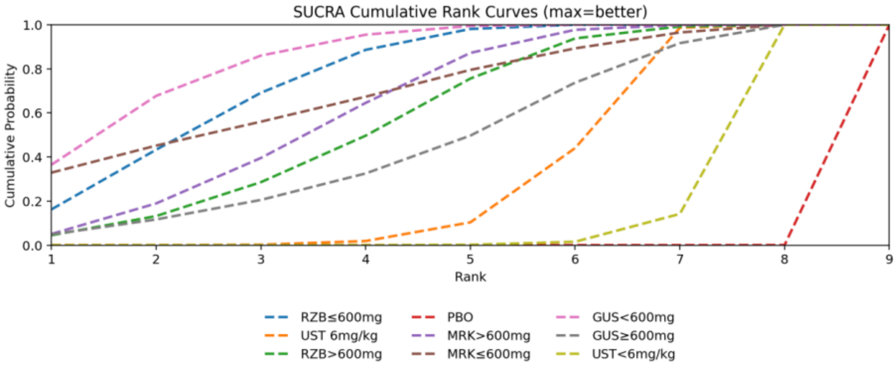

The area under the curve (AUC) for the SUCRA ranking probabilities represents the overall relative ranking of each treatment. A higher AUC indicates a treatment that is consistently ranked higher across all probability thresholds. PBO, placebo, RZB, Risankizumab, MRK, Mirikizumab, GUS, Guselkumab, UST, Ustekinumab.

Supplementary Figure S37. P-best histograms of Sensitivity analysis for inducing the clinical remission among all participants, excluding ADVANCE.

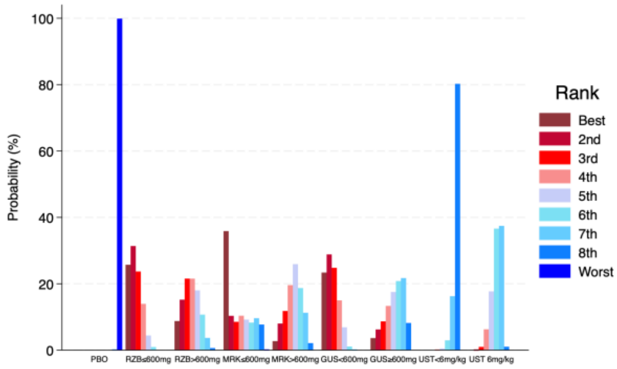

The histograms depict the probability (y-axis, expressed as a percentage) that each listed IL-23 or IL-12/23 inhibitor is ranked at a specific efficacy position (x-axis) for achieving clinical remission after induction treatment. Ranks range from best (most effective) to worst (least effective). Each bar cluster corresponds to one treatment regimen, with the bar height at a given rank representing the estimated probability for that rank.PBO, placebo, RZB, Risankizumab, MRK, Mirikizumab, GUS, Guselkumab, UST, Ustekinumab.

Supplementary Figure S38. SUCRA cumulative rank curves of Sensitivity analysis for inducing the clinical response among all participants, excluding ADVANCE.

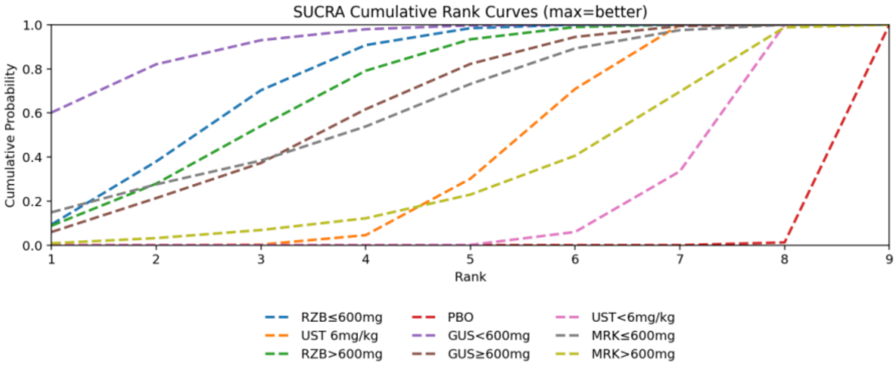

The area under the curve (AUC) for the SUCRA ranking probabilities represents the overall relative ranking of each treatment. A higher AUC indicates a treatment that is consistently ranked higher across all probability thresholds. PBO, placebo, RZB, Risankizumab, MRK, Mirikizumab, GUS, Guselkumab, UST, Ustekinumab.

Supplementary Figure S39. P-best histograms of Sensitivity analysis for inducing the clinical response among all participants, excluding ADVANCE.

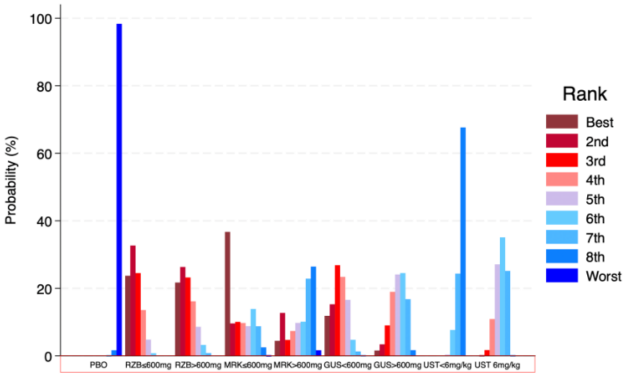

The histograms depict the probability (y-axis, expressed as a percentage) that each listed IL-23 or IL-12/23 inhibitor is ranked at a specific efficacy position (x-axis) for achieving clinical response after induciton treatment. Ranks range from best (most effective) to worst (least effective). Each bar cluster corresponds to one treatment regimen, with the bar height at a given rank representing the estimated probability for that rank.PBO, placebo, RZB, Risankizumab, MRK, Mirikizumab, GUS, Guselkumab, UST, Ustekinumab.

Supplementary Figure S40. Network plot for clinical and endoscopic outcomes among previous bio-failure participants for induction phase.

Induction

(a) clinical remission

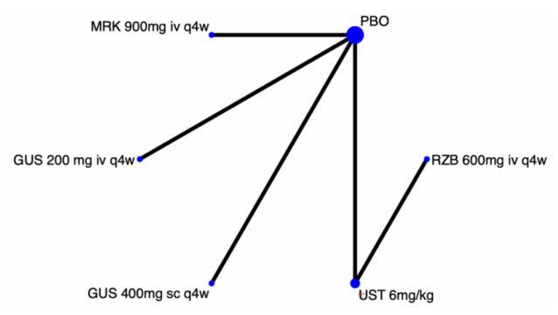

(a) clinical response

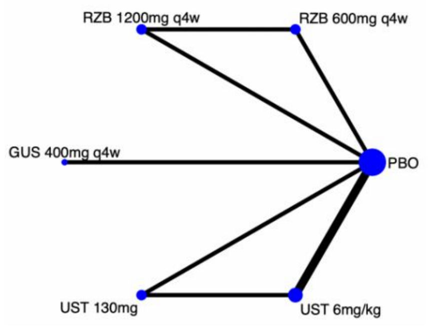

(b) endoscopic remission

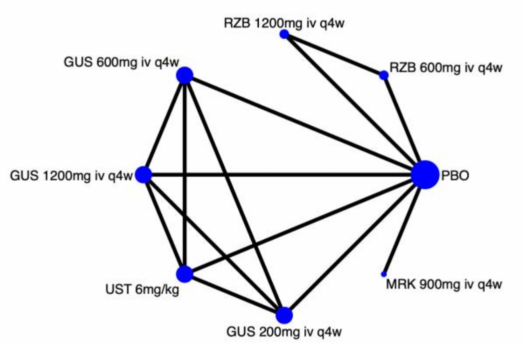

(b) endoscopic response

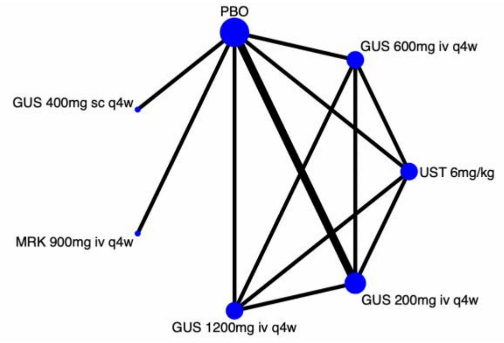

The size of each circle (node) reflects the number of participants assigned to that intervention, while the thickness of the lines (connections) indicates how many studies have directly compared the linked interventions. PBO, placebo, RZB, Risankizumab, MRK, Mirikizumab, GUS, Guselkumab, UST, Ustekinumab, q4w, every 4 weeks, q8w, every 8 weeks

Supplementary Figure S41. League table of interventions' efficacy on inducing and maintaining clinical outcomes with placebo as reference among previous bio-naive participants.

(a) clinical remission-induction

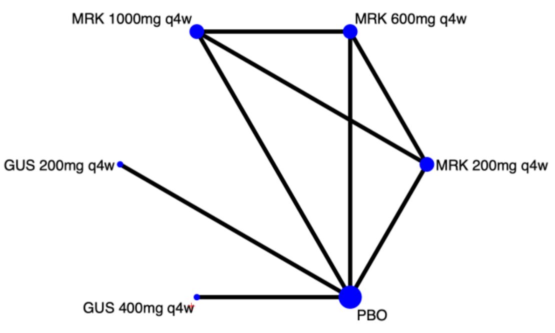

(b) clinical response-induction

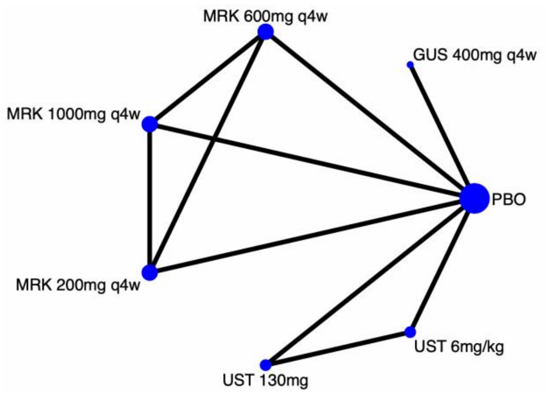

(c) endoscopic response-induction

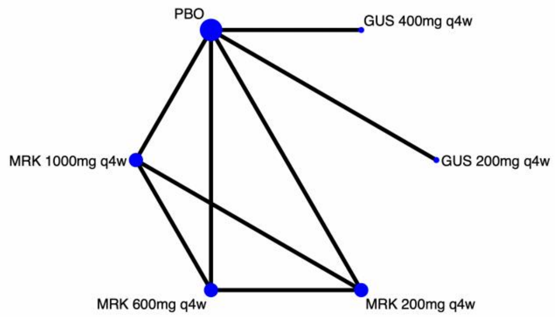

(d) clinical remission-maintenance

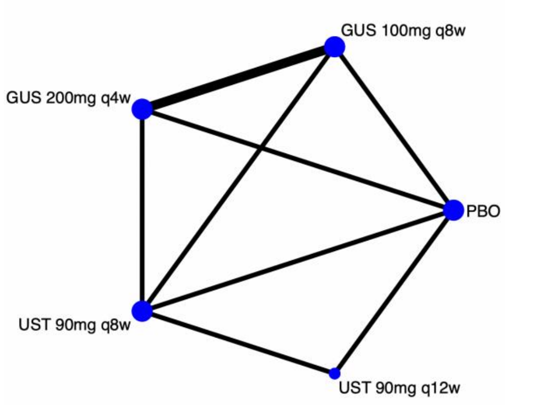

The size of each circle (node) reflects the number of participants assigned to that intervention, while the thickness of the lines (connections) indicates how many studies have directly compared the linked interventions. PBO, placebo, RZB, Risankizumab, MRK, Mirikizumab, GUS, Guselkumab, UST, Ustekinumab, q4w, every 4 weeks, q8w, every 8 weeks, q12w, every 12 weeks.

Supplementary Figure S42. League table of interventions' efficacy on inducing clinical outcomes with placebo as reference among previous bio-failure participants.

(a) clinical remission

PBO					
4.26 (2.42,7.50)	RZB 600mg IV q4w				
1.44 (0.98,2.11)	0.34 (0.17,0.67)	MRK 900mg IV q4w			
2.39 (1.49,3.83)	0.56 (0.27,1.17)	1.66 (0.91,3.05)	GUS 200 mg IV q4w		
3.54 (1.92,6.55)	0.83 (0.36,1.92)	2.46 (1.20,5.08)	1.48 (0.68,3.21)	GUS 400mg sc q4w	
2.87 (1.73,4.75)	0.67 (0.52,0.87)	1.99 (1.06,3.75)	1.20 (0.60,2.39)	0.81 (0.36,1.79)	UST 6mg/kg

(b) clinical response

PBO					
1.72 (1.27,2.32)	RZB 600mg q4w				
1.82 (1.35,2.45)	1.06 (0.90,1.24)	RZB 1200mg q4w			
2.75 (1.77,4.27)	1.60 (0.94,2.73)	1.51 (0.89,2.57)	GUS 400mg q4w		
1.14 (0.62,2.09)	0.66 (0.34,1.31)	0.63 (0.32,1.23)	0.42 (0.20,0.88)	UST 130mg	
1.13 (0.66,1.92)	0.66 (0.36,1.21)	0.62 (0.34,1.14)	0.41 (0.21,0.82)	0.99 (0.52,1.89)	UST 6mg/kg

(c) endoscopic remission

PBO							
3.58 (1.45,8.84)	RZB 600mg iv q4w						
3.02 (1.21,7.53)	0.84 (0.54,1.31)	RZB 1200mg iv q4w					
3.45 (0.82,14.50)	0.96 (0.18,5.25)	1.14 (0.21,6.26)	MRK 900mg iv q4w				
1.92 (0.39,9.48)	0.54 (0.09,3.36)	0.63 (0.10,4.00)	0.56 (0.06,4.76)	GUS 200mg iv q4w			
0.46 (0.04,4.74)	0.13 (0.01,1.57)	0.15 (0.01,1.86)	0.13 (0.01,2.06)	0.24 (0.03,2.00)	GUS 600mg iv q4w		
1.28 (0.23,7.00)	0.36 (0.05,2.45)	0.42 (0.06,2.91)	0.37 (0.04,3.43)	0.67 (0.17,2.68)	2.78 (0.31,24.99)	GUS 1200mg iv q4w	
0.44 (0.04,4.56)	0.12 (0.01,1.51)	0.15 (0.01,1.79)	0.13 (0.01,1.98)	0.23 (0.03,1.92)	0.96 (0.06,14.55)	0.35 (0.04,3.12)	UST 6mg/kg

(d) endoscopic response

PBO						
3.24 (1.62,6.46)	MRK 900mg iv q4w					
3.72 (1.84,7.50)	1.15 (0.43,3.08)	GUS 200 mg iv q4w				
1.96 (1.02,3.77)	0.61 (0.23,1.57)	0.53 (0.20,1.38)	GUS 400mg sc q4w			
3.28 (1.29,8.35)	1.01 (0.32,3.24)	0.88 (0.41,1.92)	1.67 (0.54,5.22)	GUS 600mg iv q4w		
2.66 (1.00,7.06)	0.82 (0.25,2.72)	0.72 (0.31,1.63)	1.35 (0.42,4.38)	0.81 (0.34,1.91)	GUS 1200mg iv q4w	
1.97 (0.67,5.81)	0.61 (0.17,2.20)	0.53 (0.21,1.37)	1.00 (0.28,3.55)	0.60 (0.23,1.59)	0.74 (0.27,2.04)	UST 6mg/kg

Results were expressed as RR With 95% Confidence Intervals. Comparisons after combining the network meta-analysis of direct and indirect effects, row versus column, should be read from left to right. PBO, placebo, RZB, Risankizumab, MRK, Mirikizumab, GUS, Guselkumab, UST, Ustekinumab, q4w, every 4 weeks.

Supplementary Figure S43. League table of interventions' efficacy on inducing and maintaining clinical outcomes with placebo as reference among previous bio-naive participants.

(a) clinical remission-induction

PBO					
1.75 (0.63,4.84)	MRK 200mg q4w				
1.94 (0.74,5.09)	1.11 (0.45,2.70)	MRK 600mg q4w			
1.85 (0.76,4.47)	1.06 (0.47,2.36)	0.95 (0.46,1.99)	MRK 1000mg q4w		
3.30 (1.85,5.88)	1.88 (0.58,6.07)	1.70 (0.55,5.24)	1.78 (0.62,5.13)	GUS 200mg q4w	
1.98 (1.21,3.24)	1.13 (0.37,3.50)	1.02 (0.35,3.02)	1.07 (0.39,2.95)	0.60 (0.28,1.29)	GUS 400mg q4w

(b) clinical response-induction

PBO						
1.80 (1.25,2.59)	GUS 400mg q4w					
11.85 (0.66,211.62)	6.57 (0.36,120.06)	MRK 200mg q4w				
14.14 (0.82,243.00)	7.84 (0.45,137.91)	1.19 (0.37,3.87)	MRK 600mg q4w			
16.08 (0.99,260.85)	8.92 (0.54,148.11)	1.36 (0.49,3.80)	1.14 (0.46,2.84)	MRK 1000mg q4w		
1.66 (1.25,2.20)	0.92 (0.58,1.46)	0.14 (0.01,2.53)	0.12 (0.01,2.04)	0.10 (0.01,1.69)	UST 130mg	
1.73 (1.30,2.29)	0.96 (0.60,1.52)	0.15 (0.01,2.64)	0.12 (0.01,2.13)	0.11 (0.01,1.77)	1.04 (0.85,1.28)	UST 6mg/kg

(c) endoscopic response-induction

PBO					
2.80 (1.61,4.85)	GUS 200mg q4w				
1.81 (1.13,2.92)	0.65 (0.31,1.34)	GUS 400mg q4w			
4.37 (1.00,19.19)	1.56 (0.32,7.58)	2.41 (0.51,11.40)	MRK 200mg q4w		
4.85 (1.14,20.52)	1.73 (0.37,8.12)	2.67 (0.58,12.21)	1.11 (0.45,2.70)	MRK 600mg q4w	
4.20 (1.03,17.08)	1.50 (0.33,6.78)	2.32 (0.53,10.19)	0.96 (0.42,2.19)	0.87 (0.41,1.85)	MRK 1000mg q4w

(d) clinical remission-maintenance

PBO				
1.65 (1.22,2.23)	GUS 100mg q8w			
1.74 (1.29,2.34)	1.05 (0.92,1.20)	GUS 200mg q4w		
1.65 (1.24,2.20)	1.00 (0.87,1.15)	0.95 (0.83,1.09)	UST 90mg q8w	
1.33 (0.95,1.88)	0.81 (0.58,1.12)	0.77 (0.55,1.06)	0.81 (0.60,1.09)	UST 90mg q12w

Results were expressed as RR with 95% Confidence Intervals. Comparisons after combining the network meta-analysis of direct and indirect effects, row versus column, should be read from left to right. PBO, placebo, RZB, Risankizumab, MRK, Mirikizumab, GUS, Guselkumab, UST, Ustekinumab, q4w, every 4 weeks, q8w, every 8 weeks, q12w, every 12 weeks.

Supplementary Figure S44. SUCRA cumulative rank curves of sensitivity analysis for maintaining the clinical remission among all participants, excluding SEQUENCE and CERTIFI.

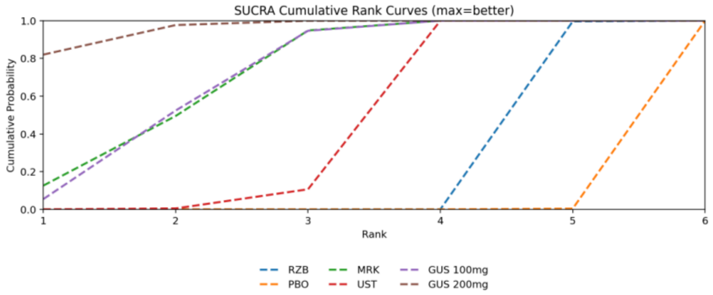

The area under the curve (AUC) for the SUCRA ranking probabilities represents the overall relative ranking of each treatment. A higher AUC indicates a treatment that is consistently ranked higher across all probability thresholds. PBO, placebo, RZB, Risankizumab, MRK, Mirikizumab, GUS, Guselkumab, UST, Ustekinumab.

Supplementary Figure S45.P-best histograms of sensitivity analysis for maintaining the clinical remission among all participants, excluding SEQUENCE and CERTIFI.

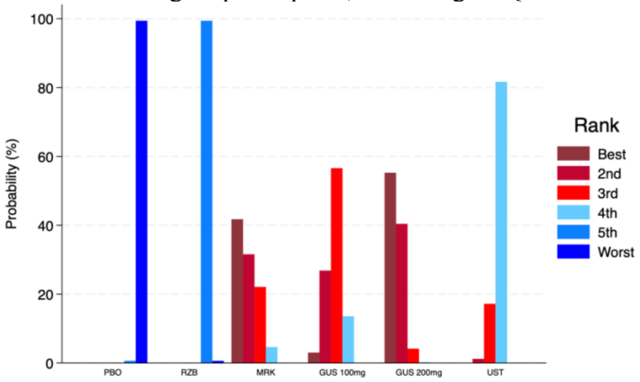

The histograms depict the probability (y-axis, expressed as a percentage) that each listed IL-23 or IL-12/23 inhibitor is ranked at a specific efficacy position (x-axis) for achieving clinical remission after maintenance treatment. Ranks range from best (most effective) to worst (least effective). Each bar cluster corresponds to one treatment regimen, with the bar height at a given rank representing the estimated probability for that rank.PBO, placebo, RZB, Risankizumab, MRK, Mirikizumab, GUS, Guselkumab, UST, Ustekinumab.

Supplementary Figure S46. SUCRA cumulative rank curves of sensitivity analysis for maintaining the clinical response among all participants, excluding CERTIFI.

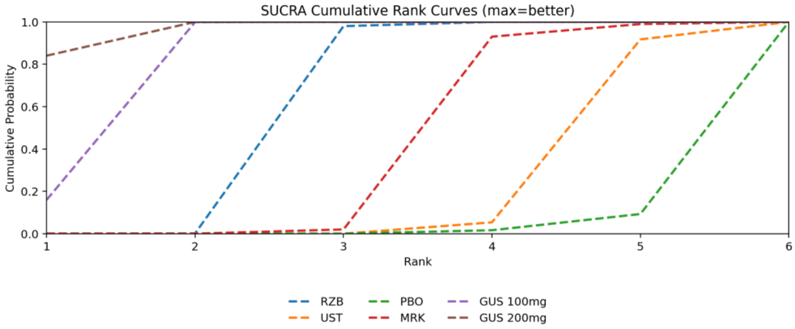

The area under the curve (AUC) for the SUCRA ranking probabilities represents the overall relative ranking of each treatment. A higher AUC indicates a treatment that is consistently ranked higher across all probability thresholds. PBO, placebo, RZB, Risankizumab, MRK, Mirikizumab, GUS, Guselkumab, UST, Ustekinumab.

Supplementary Figure S47.P-best histograms of sensitivity analysis for maintaining the clinical response among all participants, excluding CERTIFI.

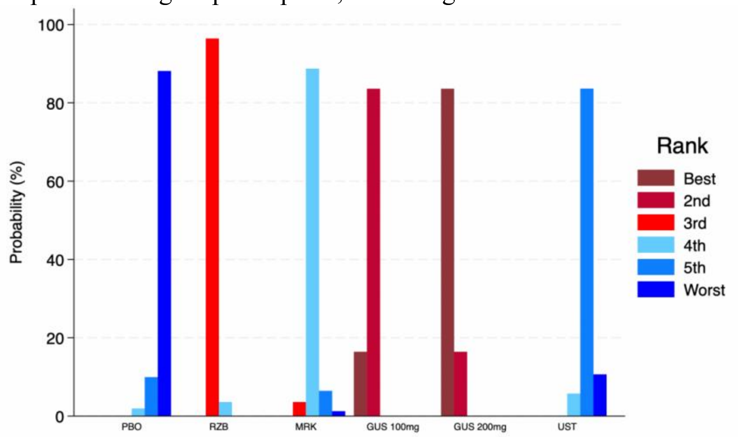

The histograms depict the probability (y-axis, expressed as a percentage) that each listed IL-23 or IL-12/23 inhibitor is ranked at a specific efficacy position (x-axis) for achieving clinical response after maintenance treatment. Ranks range from best (most effective) to worst (least effective). Each bar cluster corresponds to one treatment regimen, with the bar height at a given rank representing the estimated probability for that rank.PBO, placebo, RZB, Risankizumab, MRK, Mirikizumab, GUS, Guselkumab, UST, Ustekinumab.

Supplementary Figure S48. Network plot for clinical and endoscopic outcomes among previous bio-failure participants for maintenance phase.

Maintenance

(a) clinical remission

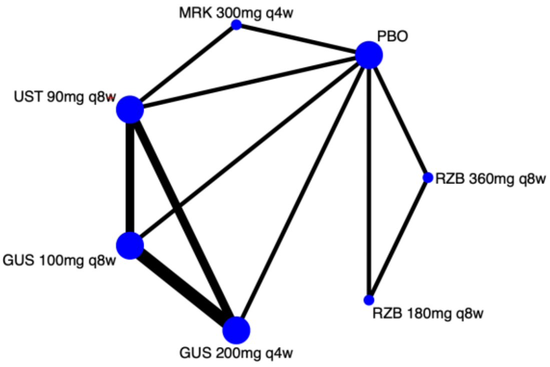

(b) endoscopic remission

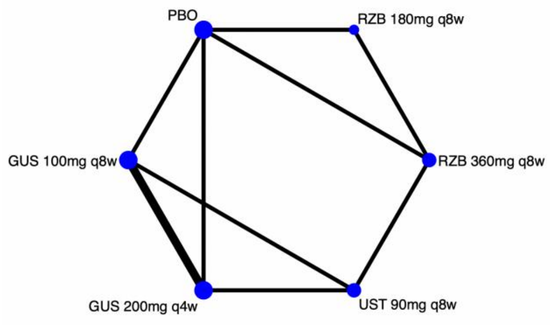

(c) endoscopic response

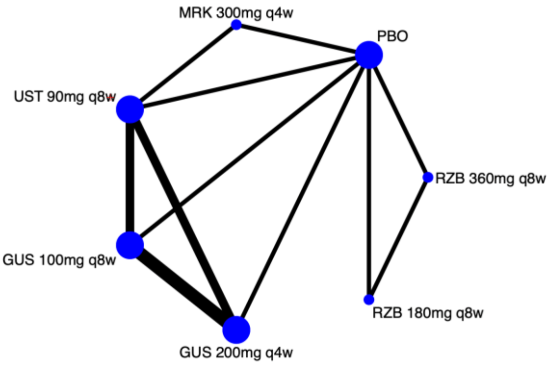

The size of each circle (node) reflects the number of participants assigned to that intervention, while the thickness of the lines (connections) indicates how many studies have directly compared the linked interventions. PBO, placebo, RZB, Risankizumab, MRK, Mirikizumab, GUS, Guselkumab, UST, Ustekinumab, q4w, every 4 weeks, q8w, every 8 weeks

Supplementary Figure S49. League table of interventions' efficacy on maintaining clinical outcomes with placebo as reference among previous bio-failure participants.
(a) clinical remission

PBO						
1.39 (1.02,1.89)	RZB 180mg q8w					
1.37 (1.00,1.88)	0.99 (0.75,1.30)	RZB 360mg q8w				
4.66 (2.92,7.45)	3.35 (1.91,5.86)	3.39 (1.93,5.96)	MRK 300mg q4w			
4.64 (2.85,7.57)	3.34 (1.87,5.94)	3.38 (1.89,6.04)	1.00 (0.75,1.32)	GUS 100mg q8w		
4.80 (2.95,7.81)	3.45 (1.94,6.13)	3.49 (1.96,6.23)	1.03 (0.78,1.36)	1.03 (0.90,1.19)	GUS 200mg q4w	
3.86 (2.40,6.19)	2.77 (1.58,4.87)	2.81 (1.59,4.95)	0.83 (0.66,1.04)	0.83 (0.70,0.99)	0.80 (0.68,0.95)	UST 90mg q8w

(b) endoscopic remission

PBO					
2.56 (0.81,8.11)	RZB 180mg q8w				
4.76 (1.46,15.56)	1.86 (0.63,5.49)	RZB 360mg q8w			
4.32 (0.50,37.14)	1.69 (0.22,13.06)	0.91 (0.18,4.58)	GUS 100mg q8w		
5.78 (0.63,53.22)	2.26 (0.27,18.58)	1.21 (0.23,6.51)	1.34 (0.62,2.88)	GUS 200mg q4w	
3.03 (0.53,17.47)	1.19 (0.23,6.12)	0.64 (0.21,1.97)	0.70 (0.25,1.95)	0.53 (0.18,1.52)	UST 90mg q8w

(c) endoscopic response

PBO						
2.00 (1.32,3.03)	RZB 180mg q8w					
2.17 (1.44,3.28)	1.08 (0.79,1.48)	RZB 360mg q8w				
8.17 (3.83,17.44)	4.08 (1.72,9.68)	3.77 (1.59,8.93)	MRK 300mg q4w			
10.13 (4.51,22.77)	5.06 (2.04,12.56)	4.67 (1.88,11.58)	1.24 (0.86,1.78)	GUS 100mg q8w		
11.71 (5.23,26.25)	5.85 (2.36,14.49)	5.40 (2.18,13.36)	1.43 (1.01,2.04)	1.16 (0.95,1.41)	GUS 200mg q4w	
7.28 (3.37,15.72)	3.63 (1.51,8.71)	3.35 (1.40,8.03)	0.89 (0.70,1.13)	0.72 (0.55,0.94)	0.62 (0.48,0.81)	UST 90mg q8w

Results were expressed as RR with 95% Confidence Intervals. Comparisons after combining the network meta-analysis of direct and indirect effects, row versus column, should be read from left to right. Statistically significant values are highlighted in green. PBO, placebo, RZB, Risankizumab, MRK, Mirikizumab, GUS, Guselkumab, UST, Ustekinumab, q4w, every 4 weeks, q8w, every 8 weeks.

Supplementary Figure S50. SUCRA cumulative rank curves of Sensitivity analysis for maintaining the endoscopic remission among all participants, excluding FORTIFY.

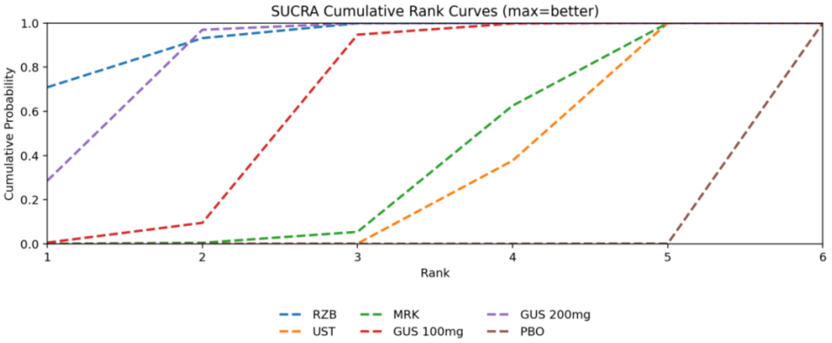

The area under the curve (AUC) for the SUCRA ranking probabilities represents the overall relative ranking of each treatment. A higher AUC indicates a treatment that is consistently ranked higher across all probability thresholds. PBO, placebo, RZB, Risankizumab, MRK, Mirikizumab, GUS, Guselkumab, UST, Ustekinumab.

Supplementary Figure S51.P-best histograms of Sensitivity analysis for maintaining the endoscopic remission among all participants, excluding FORTIFY.

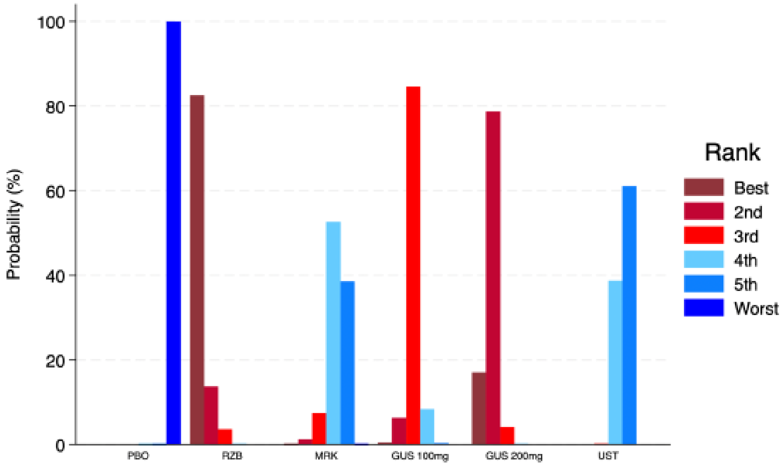

The histograms depict the probability (y-axis, expressed as a percentage) that each listed IL-23 or IL-12/23 inhibitor is ranked at a specific efficacy position (x-axis) for achieving endoscopic remission after maintenance treatment. Ranks range from best (most effective) to worst (least effective). Each bar cluster corresponds to one treatment regimen, with the bar height at a given rank representing the estimated probability for that rank.PBO, placebo, RZB, Risankizumab, MRK, Mirikizumab, GUS, Guselkumab, UST, Ustekinumab.

Supplementary Figure S52. SUCRA cumulative rank curves of sensitivity analysis for maintaining the endoscopic response among all participants, excluding FORTIFY.

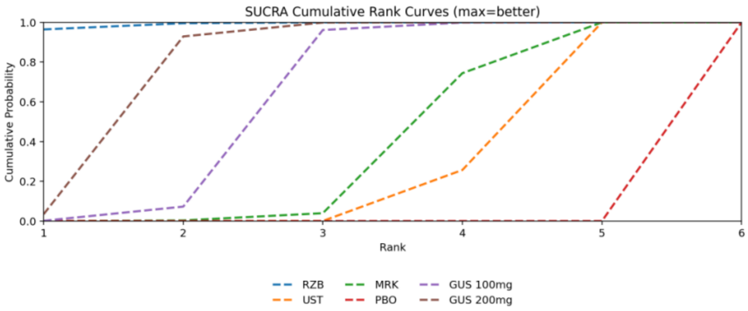

The area under the curve (AUC) for the SUCRA ranking probabilities represents the overall relative ranking of each treatment. A higher AUC indicates a treatment that is consistently ranked higher across all probability thresholds. PBO, placebo, RZB, Risankizumab, MRK, Mirikizumab, GUS, Guselkumab, UST, Ustekinumab.

Supplementary Figure S53. P-best histograms of sensitivity analysis for maintaining the endoscopic response among all participants, excluding FORTIFY.

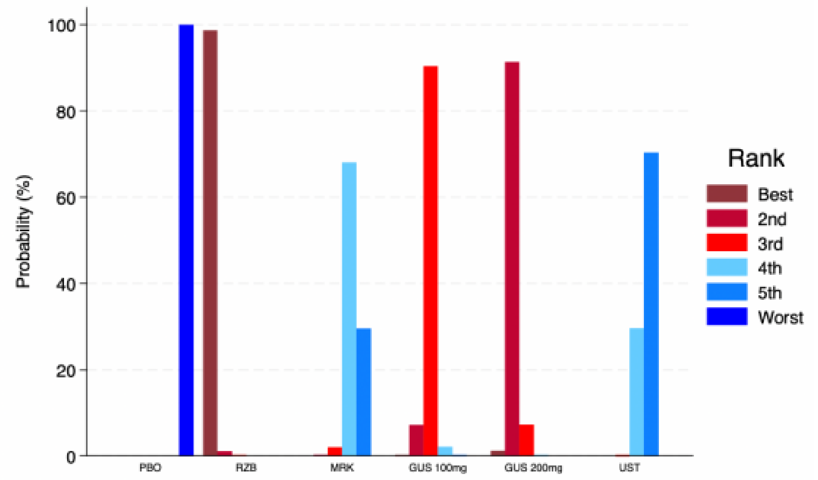

The histograms depict the probability (y-axis, expressed as a percentage) that each listed IL-23 or IL-12/23 inhibitor is ranked at a specific efficacy position (x-axis) for achieving endoscopic response after maintenance treatment. Ranks range from best (most effective) to worst (least effective). Each bar cluster corresponds to one treatment regimen, with the bar height at a given rank representing the estimated probability for that rank.PBO, placebo, RZB, Risankizumab, MRK, Mirikizumab, GUS, Guselkumab, UST, Ustekinumab.

Supplementary Figure S54. SUCRA cumulative rank curves of different IL-23 and IL-12/23 agents for different efficacy on clinical and endoscopic outcomes during induction phase.

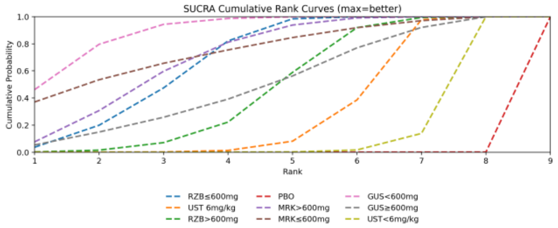

(a) clinical remission

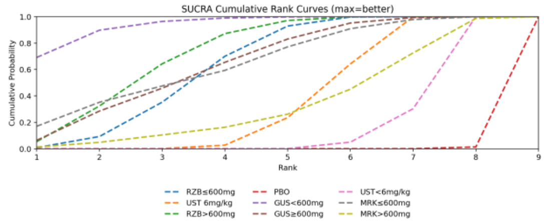

(b) clinical response

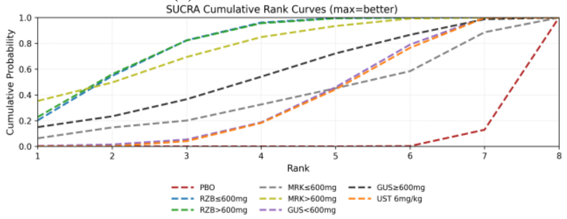

(b) endoscopic remission

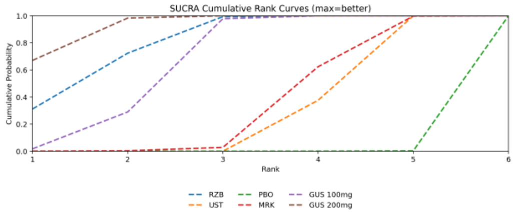

(d) endoscopic response

(a)clinical remission; (b)clinical response; (c) endoscopic remission; (d) endoscopic response. The area under the curve (AUC) for the SUCRA ranking probabilities represents the overall relative ranking of each treatment. A higher AUC indicates a treatment that is consistently ranked higher across all probability thresholds. PBO, placebo, RZB, Risankizumab, MRK, Mirikizumab, GUS, Guselkumab, UST, Ustekinumab.

Supplementary Figure S55. SUCRA cumulative rank curves of different IL-23 and IL-12/23 agents for different efficacy on clinical and endoscopic outcomes during maintenance phase.

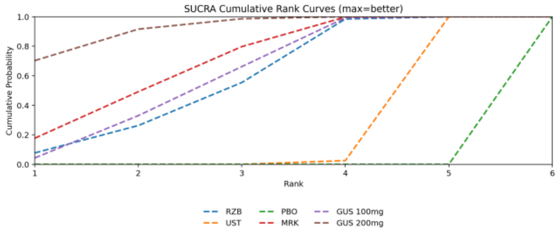

(a) clincial remission

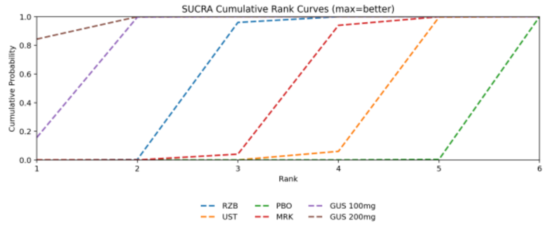

(b) clinical response

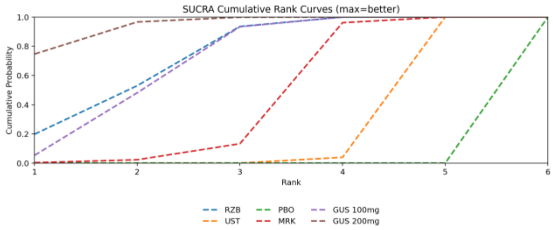

(c) endoscopic remission

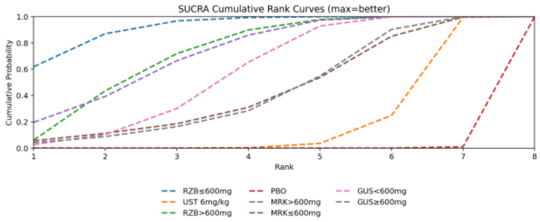

(d) endoscopic response

(a)clinical remission; (b)clinical response; (c) endoscopic remission; (d) endoscopic response. The area under the curve (AUC) for the SUCRA ranking probabilities represents the overall relative ranking of each treatment. A higher AUC indicates a treatment that is consistently ranked higher across all probability thresholds. PBO, placebo, RZB, Risankizumab, MRK, Mirikizumab, GUS, Guselkumab, UST, Ustekinumab.

Supplementary Figure S56. P-best histograms of different IL-23 and IL-12/23 agents for different efficacy on clinical and endoscopic outcomes during induction phase.

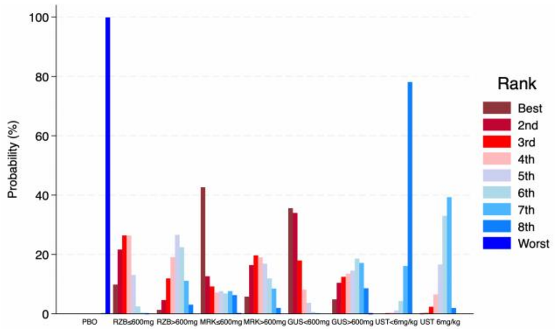

(a) clinical remission

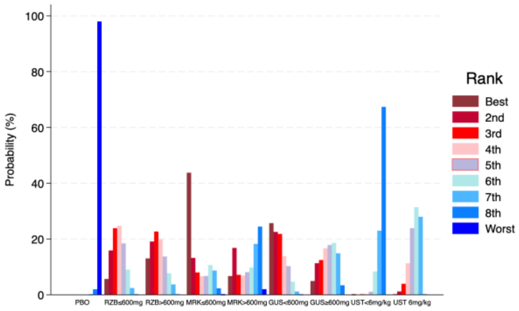

(b) clinical response

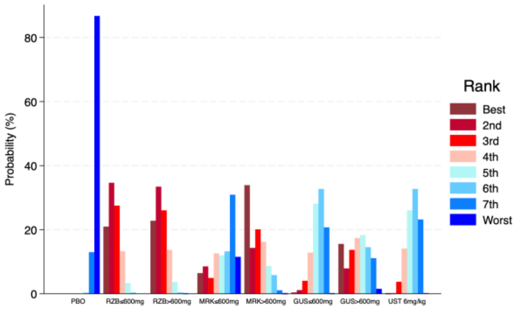

(c) endoscopic remission

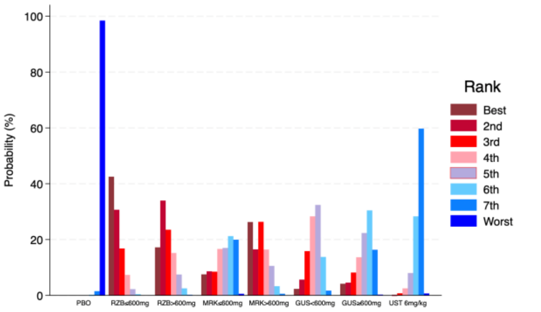

(d) endoscopic response

The histograms depict the probability (y-axis, expressed as a percentage) that each listed IL-23 or IL-12/23 inhibitor is ranked at a specific efficacy position (x-axis) for achieving (a) clinical remission, (b) clinical response, (c) endoscopic remission, and (d) endoscopic response after induction treatment. Ranks range from best (most effective) to worst (least effective). Each bar cluster corresponds to one treatment regimen, with the bar height at a given rank representing the estimated probability for that rank.PBO, placebo, RZB, Risankizumab, MRK, Mirikizumab, GUS, Guselkumab, UST, Ustekinumab.

Supplementary Figure S57. P-best histograms of different IL-23 and IL-12/23 agents for different efficacy on clinical and endoscopic outcomes during maintenance phase.

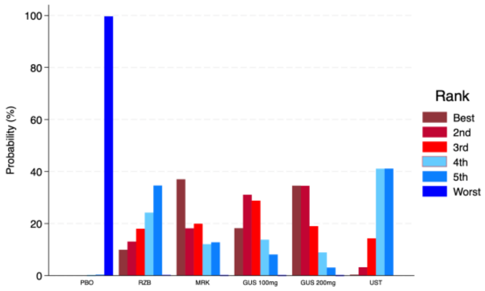

(a) clincial remission

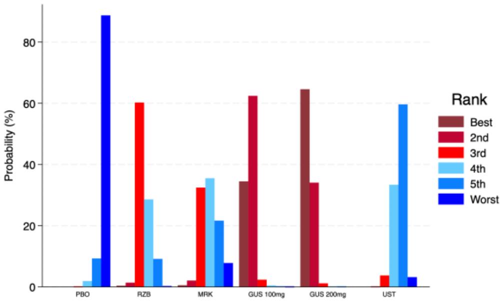

(b) clinical response

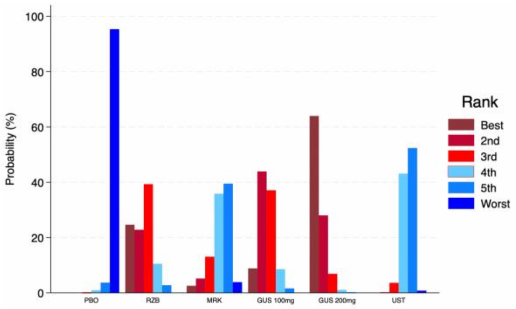

(c) endoscopic remission

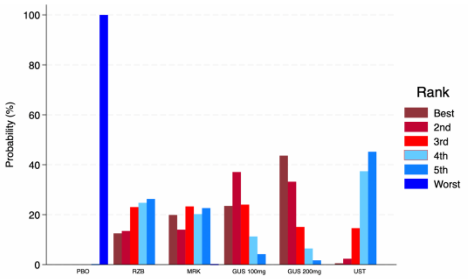

(d) endoscopic response

The histograms depict the probability (y-axis, expressed as a percentage) that each listed IL-23 or IL-12/23 inhibitor is ranked at a specific efficacy position (x-axis) for achieving (a) clinical remission, (b) clinical response, (c) endoscopic remission, and (d) endoscopic response after maintenance treatment. Ranks range from best (most effective) to worst (least effective). Each bar cluster corresponds to one treatment regimen, with the bar height at a given rank representing the estimated probability for that rank.PBO, placebo, RZB, Risankizumab, MRK, Mirikizumab, GUS, Guselkumab, UST, Ustekinumab.

Supplementary Figure S58. Funnel chart of clinical and endoscopic outcomes during induction.

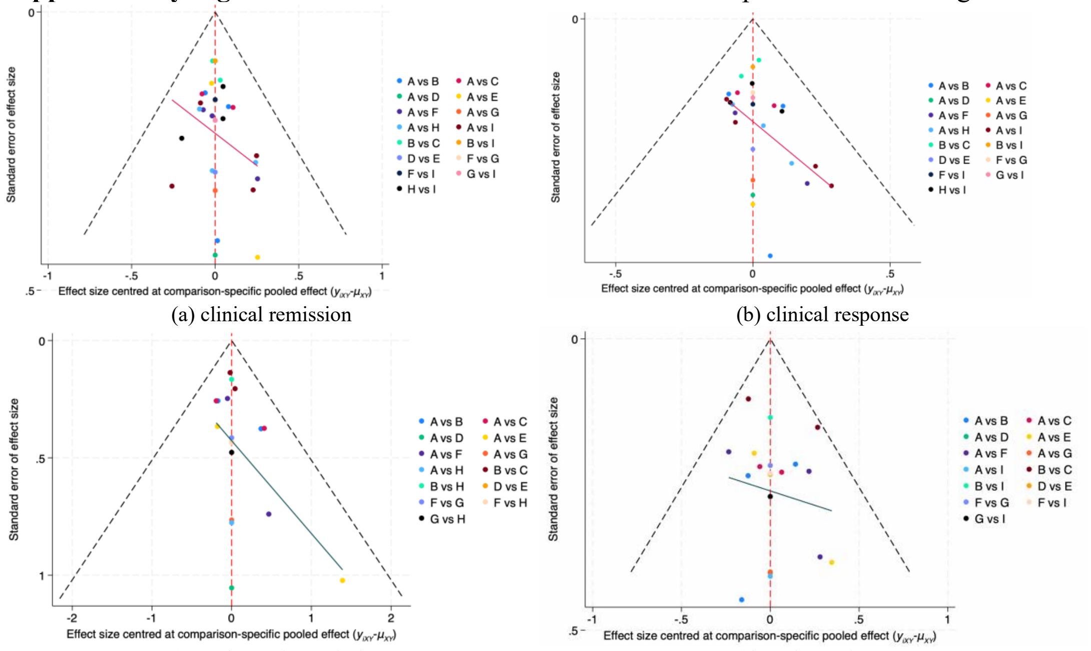

(a) clinical remission

(b) clinical response

(c) endoscopic remission

(d) endoscopic response

(a) Clinical remission. (b) Clinical response. (c) Endoscopic remission. (d) Endoscopic response. Each plot displays the effect size against its standard error. The dashed funnel lines represent the expected 95% confidence intervals around the pooled effect under the assumption of no heterogeneity. Individual points correspond to direct treatment comparisons (coded A–I as defined below). Asymmetry or points lying outside the funnel may indicate the presence of small-study effects or heterogeneity. Not all comparisons are available for every outcome. A=PBO, B=RZB≤600mg, C=RZB>600mg, D=MRK≤600mg, E=MRK>600mg, F=GUS<600mg, G=GUS≥600mg, H=UST<6mg/kg, I=UST 6mg/kg. PBO, placebo, RZB, Risankizumab, MRK, Mirikizumab, GUS, Guselkumab, UST, Ustekinumab.

Supplementary Figure S59. Funnel chart of clinical and endoscopic outcomes during maintenance.

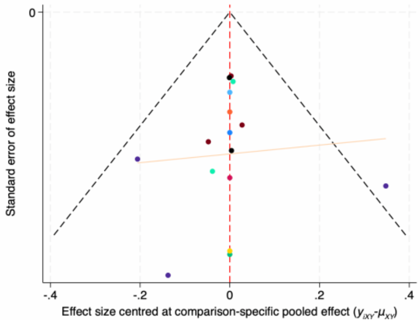

(a) clinical remission

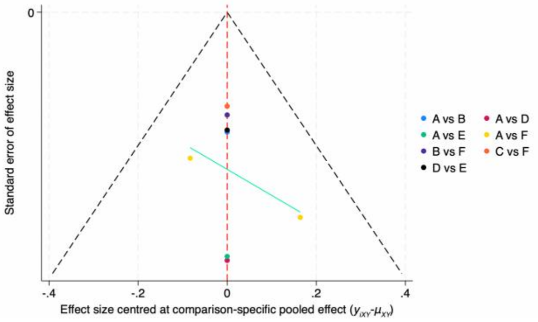

(b) clinical response

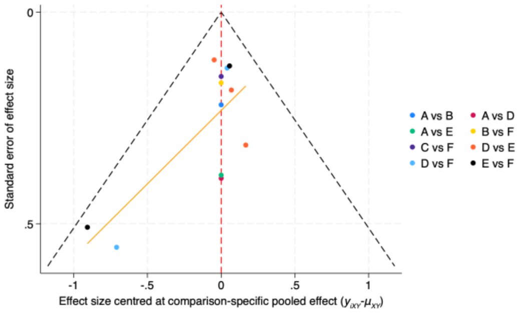

(c) endoscopic remission

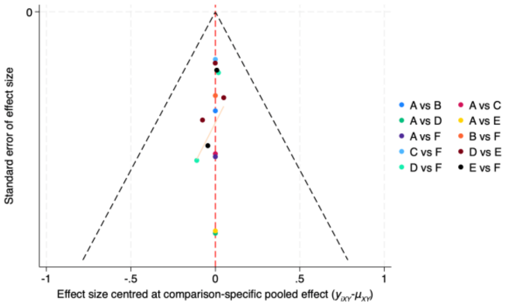

(d) endoscopic response

(a) Clinical remission. (b) Clinical response. (c) Endoscopic remission. (d) Endoscopic response. Each plot displays the effect size against its standard error. The dashed funnel lines represent the expected 95% confidence intervals around the pooled effect under the assumption of no heterogeneity. Individual points correspond to direct treatment comparisons (coded A–F as defined below). Asymmetry or points lying outside the funnel may indicate the presence of small-study effects or heterogeneity. A=PBO, B=RZB, C=MRK, D=GUS 100mg, E=GUS 200mg, F=UST. PBO, placebo, RZB, Risankizumab, MRK, Mirikizumab, GUS, Guselkumab, UST, Ustekinumab.

Supplementary Figure S60. League table of sensitivity analysis for comparative efficacy of different IL-23 and IL-12/23 agents on clinical remission and clinical response during induction phases, ADVANCE was excluded after sensitivity analysis.

	PBO	RZB≤600mg	RZB>600mg	MRK≤600mg	MRK>600mg	GUS<600mg	GUS≥600mg	UST<6mg/kg	UST 6mg/kg
PBO	PBO	2.29 (1.97,2.67)	2.26 (1.86,2.75)	2.23 (1.35,3.69)	1.80 (1.06,3.05)	2.16 (1.80,2.61)	1.94 (1.57,2.41)	1.55 (1.33,1.80)	1.88 (1.65,2.13)
RZB≤600mg	2.59 (2.12,3.18)	RZB≤600mg	0.99 (0.84,1.15)	0.98 (0.58,1.65)	0.79 (0.45,1.36)	0.94 (0.76,1.17)	0.85 (0.67,1.07)	0.67 (0.57,0.80)	0.82 (0.72,0.92)
RZB>600mg	2.37 (1.81,3.11)	0.91 (0.73,1.15)	RZB>600mg	0.99 (0.58,1.69)	0.80 (0.45,1.40)	0.96 (0.74,1.23)	0.86 (0.66,1.12)	0.68 (0.55,0.85)	0.83 (0.69,1.00)
MRK≤600mg	2.50 (1.43,4.39)	0.96 (0.53,1.75)	1.05 (0.56,1.97)	MRK≤600mg	0.81 (0.56,1.17)	0.97 (0.57,1.65)	0.87 (0.50,1.50)	0.69 (0.41,1.17)	0.84 (0.50,1.41)
MRK>600mg	2.20 (1.73,2.80)	0.85 (0.62,1.16)	0.93 (0.64,1.33)	0.88 (0.52,1.50)	MRK>600mg	1.20 (0.69,2.10)	1.08 (0.61,1.91)	0.86 (0.50,1.49)	1.04 (0.61,1.79)
GUS<600mg	2.57 (2.11,3.13)	0.99 (0.77,1.27)	1.08 (0.79,1.49)	1.03 (0.57,1.87)	1.17 (0.85,1.60)	GUS<600mg	0.90 (0.74,1.09)	0.71 (0.58,0.89)	0.87 (0.72,1.05)
GUS≥600mg	2.09 (1.49,2.95)	0.81 (0.56,1.16)	0.88 (0.58,1.34)	0.84 (0.43,1.62)	0.95 (0.63,1.45)	0.82 (0.60,1.11)	GUS≥600mg	0.80 (0.63,1.00)	0.96 (0.79,1.18)
UST<6mg/kg	1.62 (1.30,2.02)	0.62 (0.49,0.79)	0.68 (0.50,0.93)	0.65 (0.35,1.19)	0.74 (0.53,1.02)	0.63 (0.48,0.83)	0.77 (0.53,1.13)	UST<6mg/kg	1.21 (1.07,1.37)
UST 6mg/kg	1.98 (1.66,2.37)	0.76 (0.65,0.89)	0.84 (0.64,1.09)	0.79 (0.44,1.43)	0.90 (0.67,1.22)	0.77 (0.62,0.96)	0.95 (0.67,1.33)	1.23 (1.02,1.48)	UST 6mg/kg

Comparisons should be read from left to right. The risk ratio (RR) shown in each cell corresponds to the comparison between the treatment defined in the row and the treatment defined in the column. An RR greater than 1 indicates a favorable outcome for the row-defining treatment when assessing clinical remission and the column-defining treatment when assessing clinical response. Statistically significant values are highlighted in green. Results were expressed as RR with 95% Confidence Intervals.

Supplementary Figure S61. League table of sensitivity analysis for comparative efficacy of different IL-23 and IL-12/23 agents on clinical remission and clinical response during maintenance phases. SEQUENCE and CERTIFI were excluded after sensitivity analysis for clinical remission in maintenance phase; CERTIFI was excluded after sensitivity analysis for clinical response in maintenance phase.

	PBO	RZB	MRK	GUS 100mg	GUS 200mg	UST
PBO	PBO	1.48 (1.27,1.71)	1.23 (0.99,1.52)	3.05 (2.10,4.43)	3.34 (2.31,4.81)	1.10 (0.94,1.29)
RZB	1.32 (1.07,1.63)	RZB	0.83 (0.69,1.01)	2.07 (1.38,3.09)	2.26 (1.52,3.36)	0.75 (0.65,0.85)
MRK	2.71 (2.16,3.40)	2.05 (1.50,2.80)	MRK	2.48 (1.61,3.81)	2.71 (1.77,4.15)	0.90 (0.78,1.03)
GUS 100mg	2.57 (2.04,3.24)	1.94 (1.42,2.66)	0.95 (0.80,1.13)	GUS 100mg	1.09 (0.92,1.30)	0.36 (0.24,0.54)
GUS 200mg	2.75 (2.19,3.47)	2.08 (1.52,2.85)	1.02 (0.86,1.20)	1.07 (0.98,1.17)	GUS 200mg	0.33 (0.22,0.49)
UST	2.42 (1.94,3.01)	1.83 (1.35,2.48)	0.89 (0.78,1.02)	0.94 (0.85,1.05)	0.88 (0.79,0.97)	UST

Comparisons should be read from left to right. The risk ratio (RR) shown in each cell corresponds to the comparison between the treatment defined in the row and the treatment defined in the column. An RR greater than 1 indicates a favorable outcome for the row-defining treatment when assessing clinical remission and the column-defining treatment when assessing clinical response. Statistically significant values are highlighted in green. Results were expressed as RR with 95% Confidence Intervals.

Supplementary Figure S62. League table of sensitivity analysis comparative efficacy of different IL-23 and IL-12/23 agents on endoscopic remission and endoscopic response during maintenance phase, FORTIFY was excluded after sensitivity analysis.

	PBO	RZB	MRK	GUS 100mg	GUS 200mg	UST
PBO	PBO	10.55 (6.62,16.80)	5.34 (3.64,7.84)	6.68 (4.47,9.98)	7.36 (4.94,10.96)	5.12 (3.49,7.51)
RZB	7.57 (3.27,17.52)	RZB	0.51 (0.37,0.69)	0.63 (0.46,0.87)	0.70 (0.51,0.95)	0.49 (0.37,0.63)
MRK	4.05 (1.77,9.28)	0.54 (0.34,0.83)	MRK	1.25 (1.00,1.56)	1.38 (1.11,1.71)	0.96 (0.83,1.11)
GUS 100mg	5.28 (2.50,11.14)	0.70 (0.46,1.05)	1.30 (0.89,1.91)	GUS 100mg	1.10 (0.97,1.25)	0.77 (0.65,0.91)
GUS 200mg	6.23 (2.96,13.10)	0.82 (0.55,1.23)	1.54 (1.05,2.25)	1.18 (0.99,1.41)	GUS 200mg	0.70 (0.59,0.82)
UST	3.87 (1.79,8.37)	0.51 (0.37,0.71)	0.95 (0.71,1.28)	0.73 (0.57,0.94)	0.62 (0.49,0.79)	UST

Comparisons should be read from left to right. The risk ratio (RR) shown in each cell corresponds to the comparison between the treatment defined in the row and the treatment defined in the column. An RR greater than 1 indicates a favorable outcome for the row-defining treatment when assessing endoscopic remission and the column-defining treatment when assessing endoscopic response. Statistically significant values are highlighted in green. Results were expressed as RR with 95% Confidence Intervals.

REFERENCE

1.Peyrin-Biroulet, L.;Chapman, J. C.;Colombel, J. F.;Caprioli, F.;D'Haens, G.;Ferrante, M.;Schreiber, S.;Atreya, R.;Danese, S.;Lindsay, J. O.*, et al.* Risankizumab versus ustekinumab for moderate-to-severe crohn's disease. N Engl J Med 2024, 391, 213-223.
2.Ferrante, M.;Panaccione, R.;Baert, F.;Bossuyt, P.;Colombel, J. F.;Danese, S.;Dubinsky, M.;Feagan, B. G.;Hisamatsu, T.;Lim, A.*, et al.* Risankizumab as maintenance therapy for moderately to severely active crohn's disease: Results from the multicentre, randomised, double-blind, placebo-controlled, withdrawal phase 3 fortify maintenance trial. Lancet 2022, 399, 2031-2046.
3.D'Haens, G.;Panaccione, R.;Baert, F.;Bossuyt, P.;Colombel, J. F.;Danese, S.;Dubinsky, M.;Feagan, B. G.;Hisamatsu, T.;Lim, A.*, et al.* Risankizumab as induction therapy for crohn's disease: Results from the phase 3 advance and motivate induction trials. Lancet 2022, 399, 2015-2030.
4.Feagan, B. G.;Sandborn, W. J.;D'Haens, G.;Panés, J.;Kaser, A.;Ferrante, M.;Louis, E.;Franchimont, D.;Dewit, O.;Seidler, U.*, et al.* Induction therapy with the selective interleukin-23 inhibitor risankizumab in patients with moderate-to-severe crohn's disease: A randomised, double-blind, placebo-controlled phase 2 study. Lancet 2017, 389, 1699-1709.
5.Ferrante, M.;D'Haens, G.;Jairath, V.;Danese, S.;Chen, M.;Ghosh, S.;Hisamatsu, T.;Kierkus, J.;Siegmund, B.;Bragg, S. M.*, et al.* Efficacy and safety of mirikizumab in patients with moderately-to-severely active crohn's disease: A phase 3, multicentre, randomised, double-blind, placebo-controlled and active-controlled, treat-through study. Lancet 2024, 404, 2423-2436.
6.Sands, B. E.;Peyrin-Biroulet, L.;Kierkus, J.;Higgins, P. D. R.;Fischer, M.;Jairath, V.;Hirai, F.;D'Haens, G.;Belin, R. M.;Miller, D.*, et al.* Efficacy and safety of mirikizumab in a randomized phase 2 study of patients with crohn's disease. Gastroenterology 2022, 162, 495-508.
7.Danese, S.;Panaccione, R.;Feagan, B. G.;Afzali, A.;Rubin, D. T.;Sands, B. E.;Reinisch, W.;Panés, J.;Sahoo, A.;Terry, N. A.*, et al.* Efficacy and safety of 48 weeks of guselkumab for patients with crohn's disease: Maintenance results from the phase 2, randomised, double-blind galaxi-1 trial. Lancet Gastroenterol Hepatol 2024, 9, 133-146.
8.Panaccione, R.;Feagan, B. G.;Afzali, A.;Rubin, D. T.;Reinisch, W.;Panés, J.;Danese, S.;Hisamatsu, T.;Terry, N. A.;Salese, L.*, et al.* Efficacy and safety of intravenous induction and subcutaneous maintenance therapy with guselkumab for patients with crohn's disease (galaxi-2 and galaxi-3): 48-week results from two phase 3, randomised, placebo and active comparator-controlled, double-blind, triple-dummy trials. Lancet 2025, 406, 358-375.
9.Hart, A.;Panaccione, R.;Steinwurz, F.;Danese, S.;Hisamatsu, T.;Cao, Q.;Ritter, T.;Seidler, U.;Olurinde, M.;Vetter, M. L.*, et al.* Efficacy and safety of guselkumab subcutaneous induction and maintenance in participants with moderately to severely active crohn's disease: Results from the phase 3 graviti study. Gastroenterology 2025, 169, 308-325.
10.Feagan, B. G.;Sandborn, W. J.;Gasink, C.;Jacobstein, D.;Lang, Y.;Friedman, J. R.;Blank, M. A.;Johanns, J.;Gao, L. L.;Miao, Y.*, et al.* Ustekinumab as induction and maintenance therapy for crohn's disease. N Engl J Med 2016, 375, 1946-1960.
11.Sandborn, W. J.;Gasink, C.;Gao, L. L.;Blank, M. A.;Johanns, J.;Guzzo, C.;Sands, B. E.;Hanauer, S. B.;Targan, S.;Rutgeerts, P.*, et al.* Ustekinumab induction and maintenance therapy in refractory crohn's disease. N Engl J Med 2012, 367, 1519-1528.
12.Dziegielewski, C.;Yuan, Y.;Ma, C.;Boland, B. S.;Chang, J. T.;Syal, G.;Vuyyuru, S. K.;Peyrin-Biroulet, L.;Jairath, V.;Singh, S. Il-23p19 antagonists vs ustekinumab for treatment of crohn's disease: A meta-analysis of randomized controlled trials. Am J Gastroenterol 2025, 120, 2260-2267.
13.Strober, B.;Coates, L. C.;Lebwohl, M. G.;Deodhar, A.;Leibowitz, E.;Rowland, K.;Kollmeier, A. P.;Miller, M.;Wang, Y.;Li, S.*, et al.* Long-term safety of guselkumab in patients with psoriatic disease: An integrated analysis of eleven phase ii/iii clinical studies in psoriasis and psoriatic arthritis. Drug Saf 2024, 47, 39-57.
14.Administration, U. S. F. a. D. Integrated review for bla 761262 and bla 761105/s-016: Skyrizi (risankizumab-rzaa). U.S. Food and Drug Administration: Silver Spring, MD, 2022.Available online: https://www.accessdata.fda.gov/drugsatfda_docs/nda/2022/761262Orig1s000TOC.cfm (accessed on 3 March 2026).
15.Chua, L.;Otani, Y.;Lin, Z.;Friedrich, S.;Durand, F.;Zhang, X. C. Mirikizumab pharmacokinetics and exposure-response in patients with moderately-to-severely active crohn's disease: Results from two randomized studies. Clin Transl Sci 2025, 18, e70320.
16.Administration, U. S. F. a. D. Tremfya (guselkumab) injection: Full prescribing information. 2025.59.Available online: https://dailymed.nlm.nih.gov/dailymed/drugInfo.cfm?setid=1e6dc9ae-1c4c-42d9-87aa-c315ecc51b56 (accessed on 3 March 2026).

17.Administration, U. S. F. a. D. Stelara (ustekinumab) fda prescribing information. 2025.Available online: https://dailymed.nlm.nih.gov/dailymed/drugInfo.cfm?setid=eeaf22f6-fe1c-4ef7-9105-7b8d01e56eff (accessed on 3 March 2026).
